# Plant polysaccharides in neuroprotection: mechanistic insights across central nervous system disorders

**DOI:** 10.3389/fphar.2025.1727705

**Published:** 2026-02-11

**Authors:** Qiyue Yu, Hui Chen, Zhuofeng Qu, Jialin Qu, Yan Wang, Lina Liang, Shouyu Hu

**Affiliations:** 1 The First Affiliated Hospital, Dalian Medical University, Dalian, China; 2 Institute (College) of Integrative Medicine, Dalian Medical University, Dalian, China; 3 The Second Affiliated Hospital, Dalian Medical University, Dalian, China

**Keywords:** apoptosis, gut microbiota, neuroinflammation, neuron autophagy, neuroprotective mechanism, oxidative stress, plant polysaccharides

## Abstract

Central nervous system (CNS) disorders pose a major global health burden, and the limited efficacy and adverse effects of current pharmacotherapies underscore the need for more effective therapeutic strategies. Plant polysaccharides, as key bioactive metabolites of medicinal plants, have attracted increasing attention due to their broad biological activities and favorable safety profiles. In this review, we comprehensively summarize recent advances in understanding their neuroprotective effects, emphasizing mechanistic pathways that include the suppression of neuroinflammation, mitigation of oxidative stress, inhibition of neuronal apoptosis, modulation of autophagy, and maintenance of gut microbiota homeostasis. These mechanisms are examined across diverse preclinical models, with particular attention to model selection, dosing parameters, and experimental rigor to ensure reliable interpretation. Furthermore, we critically discuss major challenges hindering clinical translation, including structural heterogeneity, limited bioavailability, and restricted CNS penetration, while highlighting emerging solutions such as advanced delivery systems and targeted chemical modification. Overall, this review delineates the multi-pathway neuroprotective actions of plant polysaccharides and offers strategic direction for future mechanistic studies and translational development.

## 1 Introduction

Central nervous system (CNS) disorders such as stroke, Parkinson’s disease (PD), Alzheimer’s disease (AD), Huntington’s disease, constitute a major contributor to the global burden of disease, ranking as the primary contributor of disability and the second major driver of mortality worldwide. To address this critical public health challenge, the development and implementation of effective prevention, treatment, and rehabilitation strategies are warranted (Feigin et al., 2020; GBD, 2021 Nervous System Disorders Collaborators, 2024). Despite significant advancements achieved in therapeutic strategies in recent years, the efficacy of treatment remains limited due to the intricate anatomical architecture and functional mechanisms of CNS (Huang et al., 2023). For instance, the treatment of acute stroke predominantly depends on vascular recanalization, such as thrombolysis and endovascular therapy, to restore cerebral perfusion. However, the efficacy of these interventions is constrained by narrow temporal windows, which significantly limit patient eligibility for treatment (Haupt et al., 2023). In the realm of neurodegenerative diseases, clinical trials investigating novel agents such as deferiprone for PD, have yielded suboptimal outcomes. In particular, in participants with early-stage PD who had never been treated with levodopa and who had no planned use of dopaminergic therapies, deferiprone demonstrated inferior efficacy with lower scores on standardized measures of symptoms of PD compared to placebo (Devos et al., 2022). Moreover, current neuroprotective pharmacological interventions primarily involve free radical scavengers (e.g., edaravone) (Xu J. et al., 2021), anti-inflammatory agents (e.g., statins) (Muneeb et al., 2022), and anti-apoptotic compounds (e.g., minocycline) (Sanchez Mejia et al., 2001). Nonetheless, these therapies remain suboptimal, due to the emergence of adverse side effects and the development of drug resistance, limiting their long-term efficacy (Ma et al., 2019). In response to these limitations, the development of novel, efficient, low-toxicity, and long-acting neuroprotective agents has become a focal point of critical research.

Natural products represent an indispensable reservoir for novel drug discovery and play a pivotal role in promoting human health. Accumulating evidence indicates that bioactive metabolites derived from plants, fungi, and other natural sources exert a broad spectrum of pharmacological effects, including potent antioxidant, anti-inflammatory, antimicrobial, and neuroprotective activities, which collectively contribute to the preservation of neural homeostasis and the attenuation of neurodegenerative processes (Azhar et al., 2019; Daglia et al., 2014; Hamedi et al., 2020; Selamoglu et al., 2013; Sevindik et al., 2021; Sureda et al., 2023; Vasile et al., 2024).

Among these natural bioactives, plant polysaccharides have attracted growing attention as key functional metabolites of botanical drugs. As biological macromolecules, plant polysaccharides consist of monosaccharides and uronic acids linked by glycosidic bonds, with molecular weights ranging from thousands to tens of millions of Daltons (Ma et al., 2019; Ullah et al., 2019; Su et al., 2017; Hu et al., 2015). Although there are some differences in the composition of polysaccharides in different plants, the main monosaccharides are rhamnose, glucose, arabinose, and galacturonic acid (Tao et al., 2018; Zeng et al., 2020; Pan et al., 2022; Cui et al., 2019). Structurally, they are characterized by a backbone rich in 1→4 and 1→6 glycosidic linkages, as well as branched side chains (Hu et al., 2015; Zhu et al., 2022).

**FIGURE 1:**
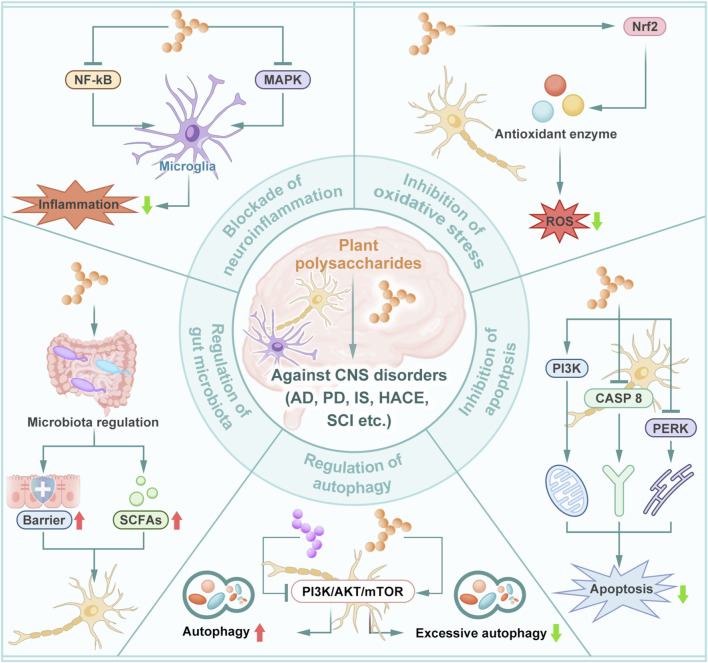
Neuroprotective mechanisms of plant polysaccharides. Plant polysaccharides exert a protective effect on the central nervous system by regulating neuroinflammation, oxidative stress, neuronal apoptosis, autophagy and gut microbiota.

Due to their unique structural characteristics, plant polysaccharides exhibit diverse biological activities, including antitumor (Meng et al., 2024), antimicrobial (Li et al., 2023), immunomodulatory (Zhang X. et al., 2021), and neuroprotective effects (Fu et al., 2025). Preclinical studies have demonstrated that plant polysaccharides can alleviate neurological deficits in various conditions, such as cerebral ischemia-reperfusion injury (Yu et al., 2018), neurodegenerative diseases (e.g., AD and PD) (Wang S. Y. et al., 2024; Mu et al., 2021; Fang et al., 2016), and toxin-induced neurotoxicity (Gao et al., 2014; Cheng et al., 2022; Xu L. et al., 2024). The neuroprotective mechanisms of plant polysaccharides involve blockade of neuroinflammation (Zhong et al., 2020), inhibition of oxidative stress and apoptosis (Zhu et al., 2022; Guo et al., 2019), regulation of autophagy (Tan et al., 2020), regulation of gut microbiota (Sun et al., 2022), and other mechanisms, as shown by Figure 1. Although numerous studies have investigated the role of plant polysaccharides in CNS disorders, further in-depth analysis and summary of the existing studies are essential to facilitate the development and clinical application of novel therapeutic agents. In this review, we summarize the latest advancements in research on the neuroprotective effects of plant polysaccharides and analyze the underlying mechanisms and pathways involved. Furthermore, the structure-activity relationships of plant polysaccharides and the applications in drug delivery systems are incorporated. The objective is to provide a foundation for the development and clinical implementation of therapeutic agents based on plant polysaccharides for CNS disorders.

## 2 Methods

In this review, a comprehensive and systematic literature search was performed in PubMed and Web of Science between January, 2014, and September, 2025. The search strategy employed relevant keywords and MeSH terms, applied individually or in Boolean combinations (AND/OR), including: “plant polysaccharides,” “neuron,” “nervous system disorders,” “Alzheimer’s disease,” “Parkinson’s disease,” “ischemic stroke,” “ischemia-reperfusion injury,” “spinal cord injury,” “brain tumors,” “optic nerve injury,” “neuroprotection,” “neuroinflammation,” “oxidative stress,” “apoptosis,” “autophagy,” “pyroptosis,” “ferroptosis,” “NF-κB,” “MAPK,” “Nrf2,” “PI3K/Akt,” “mTOR,” “M1/M2 macrophage polarization,” “gut microbiota,” and “drug delivery.”

Studies were selected according to the following criteria: After title and abstract screening, irrelevant studies were excluded manually. *In vitro* mechanistic studies: Articles exploring the cellular and molecular mechanisms of plant polysaccharides in the CNS, particularly their regulatory effects on key signaling pathways (e.g., NF-κB, MAPK, Nrf2, PI3K/Akt, and mTOR). *In vivo* preclinical studies: Experimental research using animal models of CNS disorders (e.g., Alzheimer’s disease, Parkinson’s disease, ischemic stroke, ischemia-reperfusion injury, spinal cord injury, brain tumors, and optic nerve injury), with a focus on therapeutic efficacy and translational potential.

To ensure the relevance and quality of the included studies, the following exclusion criteria were applied: Non-original research articles: Reviews, meta-analyses, editorials, conference abstracts, patents, and book chapters were excluded to base our analysis on primary data. Studies on polysaccharides of non-plant origin: Research investigating polysaccharides derived from animals, fungi (e.g., mushroom), algae, or bacteria was excluded to maintain focus on plant-derived metabolites. Studies not relevant to the CNS: Research focusing solely on the effects of plant polysaccharides on peripheral systems without any investigation of neurological outcomes or mechanisms in CNS. Publications not in English: Non-English articles were excluded due to limitations in resources for accurate translation and assessment.

To ensure comprehensiveness, studies covering a broad range of plant sources were considered, with preference given to those demonstrating robust experimental design and clear mechanistic insights. A total of 72 studies met all criteria. The details of including literature were listed at Tables 1–3. This review was prepared adhering to the principles outlined in the Four Pillars of Best Practice for Ethnopharmacology Research.

**TABLE 1:** The anti-neuroinflammatory effects of different plant polysaccharides.

Name	Polysaccharides source	Average Mw	Monosaccharide compositions	Dose	Experimental model	Involved mechanism	Ref
PTP70-2	*Polygala tenuifolia*	No mention	No mention	3,6,12 μmol/L	BV2 cell and primary cortical neuron of SD rats	Inactivates the TLR4 signaling pathway, suppressed the activation of NF-κB, repressed neuroinflammation and downregulated TNF-α, IL-6, IL-1β, COX-2 and iNOS	Chen H. et al. (2022)
ATP50-3	*Acorus tatarinowii*	87.3 kDa	Ara, Rha, Gal, Glc, Xyl, Man	2.5,5.0, 10 μM	BV2 cell	Inhibits neuroinflammation via inactivation of TLR4-mediated MyD88/NF-κB and PI3K/Akt signaling pathways	Zhong et al. (2020)
APS	*Astragalus membranaceus*	No mention	No mention	Acute study: deionized water and 200, 300, 400, 500, 600, or 800 mg/kg Chronic study: 400 mg/kg	SPF C57BL/6 mice	Affects the occurrence of epilepsy by targeting the TLR4/NF-κB signaling pathway, reducing the release of pro-inflammatory mediators, and alleviating cognitive impairment by hindering neuroinflammatory processes	Lu et al. (2024)
AMP	*Aronia melanocarpa fruit*	No mention	Ara, Gal, Glc, Xyl, Man, Gal-UA	100, 200 mg/kg	Aging mice induced by d-galactose (D-Gal)	Blocked the phosphorylation of IκB-α, an upstream regulatory factor, significantly inhibited the high expression of NF-κB and reduce D-Gal-induced inflammation	Zhao Y. et al. (2021)
SBP	*Hippophae rhamnoides L.*	58.78 kDa	Ara, Glc, Gal, Rha, Xyl, Fuc, GalA	0.1 %w/w	High-fat diet induced C57 mice	Inhibited the NF-κB signaling pathway and prevented microglial activation to suppress neuroinflammation by improving intestinal inflammation, resisting LPS invasion into the bloodstream	Lan et al. (2023)
PAP	*Potentilla anserina L*	No mention	No mention	BALB/C mice: 150, 300, 500 mg/kg Wistar rats: 100, 200, 400 mg/kg	Hypoxia and antiacute hypoxia BALB/C mice acute hypobaric hypoxia-induced Wistar rats	Blocked NF-κB and HIF-1α signaling, inhibited proinflammatory cytokine such as IL-1β, IL-6, TNF-α, and ameliorated acute hypobaric hypoxia-induced brain injury	Shi et al. (2020)
SNP2-A	*scrophularia ningpoensis*	24.0 kDa	Man, Rha, Glu A, Gal A, Glu, Gal, Xyl, Ara	10, 20 and 40 mg/kg	Cerebral ischemia/reperfusin injury (CIRI) SD rats	Ameliorated the reductive ERK protein expression and the elevated JNK and p38 protein expression stimulated by CIRI	Ma et al. (2019)
LBP	*Lycium barbarum*	No mention	No mention	10, 20 and 40 mg/kg	Mouse model of middle cerebral artery occlusion (MCAO) mice	Inhibited the expression and activation of p38-MAPK and NF-κB in the hippocampus, decreased the expression of TNF-α and IL-1β in activated glial cells, decreased the activation of astrocytes and microglia	Zhao et al. (2017)
SCP	*Schisandra Chinensis Fructus*	No mention	Man, Rha, GlcA, Glc, Gal, Ara	260 mg/kg	AD mice	Decreased Aβ deposition, activated the NF-κB/MAPK pathway, controlled phosphorylation of p-38, JNK, ERK, reduced nuclear translocation of NF-κB to relieve the release of proinflammatory cytokines including IL-1β,1L-6 and TNF-α, improve astrocyte and microglia activation	Xu et al. (2019)
APS	*Astragalus membranaceus*	No mention	Glc, Gal, Ara, Xyl, Man, GalA, Rha	200 mg/kg	C57BL/6 mice	Reduced the phosphorylation of key kinases triggered by LPS stimulation, including JNK1/2, ERK1/2, p38, IκB and p65, indicating that it could effectively inhibit the activation of MAPK and NF-κB signaling pathways and suppress the expression of pro-inflammatory cytokines IL-1β, TNF-α and IL-6	Liu D. et al. (2025)
APS	*Astragalus membranaceus*	No mention	No mention	Cells: 100 mg/L Animals: 22.5 and 45 mg/kg	HAPI cells and MCAO SD rat model	Promoted the degradation of ATP in microglia and inhibiting the expression of P2X7R	Jia et al. (2022)
SCP2-1	*Schisandra Chinensis Fructus*	No mention	Rha, Fuc, Ara, Xyl, Man, Glc, Gal	Cells: 0, 6.25, 12.5, 25, 50 μg/mL Animals: 28 and 56 mg/kg	BV2 cell and mice	Increased lipoprotein receptor-related protein-1 (LRP-1), which binds and suppresses the activatied NF-κB and MAPK pathways, particularly the JNK target, which reversed LPS-induced polarization of M1/M2 microglia, thereby reducing nerve inflammation	Xu et al. (2020)

**TABLE 2:** The inhibitory effects of different plant polysaccharides on oxidative stress.

Name	Polysaccharides source	Average Mw	Monosaccharide compositions	Dose	Experimental model	Involved mechanism	Ref
ARP-1	*Asteris Radix et Rhizoma*	214 kDa	Fuc, Ara, Gal, Glc, Man	0.2, 1.0 and 2.0 mg/mL	PC12 cell	Scavenged and reduced ABTS, hydroxy and DPPH radicals and decreased the production of ROS and MDA, and improved the activities of SOD.	Zhu et al. (2022)
EbPS-A1	*Epimedium*	No mention	Man, Fuc, GalA, Gal, Glc, Rha, Ara	Chemosensory behavior assay: 1–2 mg/mL (3 days) PolyQ aggregation assay: EbPS-A1 2 mg/mL Determination of body bends: 2 mg/mL Lifespan assay: EbPS-A1 2 mg/mL Paraquat resistance assay: EbPS-A1 2 mg/mL (24 h) Free radical scavenging: EbPS-A1 >8 mg/mL and 0.5–8 mg/mL ROS/enzymes/MDA analysis: 2 mg/mL (3 days	*C. elegans*	Scavenged hydroxyl radical, DPPH, and other free radicals, thus reducing ROS levels, increased SOD activity and reduced MDA content	Xiang et al. (2017)
PGP1	*Platycodon grandiflorum*	5.9 kDa	Glc, Gal, Man	50, 100 and 200 μg/mL	PC12 cell	Increased SOD activity in PC12 cells, weaken the formation of intracellular ROS, reduce MDA levels, LDH release and lipid peroxidation generation	Sheng et al. (2017)
LICP009-3F-1a	*Lycium barbarum*	10.78 kDa	Ara, Gal, Glc, Xyl, Man	10, 50, 100, 200, and 500 μg/mL	PC12 cell	Inhibited CoCl2-induced oxidative stress in PC12 cells under hypoxic injury by activating the SOD, CAT, and GPx genes in PC12 cells, which enhanced the activities of antioxidant enzymes, such as SOD, CAT, and GPx, thereby effectively reducing ROS levels	Li Y. et al. (2025)
CDP	*Cistanche deserticola*	No mention	No mention	0.05, 0.5 and 5 μg/mL	PC12 cell	Decreased intracellular ROS levels, increased GSH-Px, CAT and total antioxidant capacity levels, and regulated the DJ-1 pathway	Liu et al. (2018)
PSPE	*Polygonatum sibiricum*	252.5 kDa and 1277 Da	Ara, Gal, Glc, Man, GalA	Body bends and head thrashes: 0, 2, 10, 20, 100, 200, and 400 μg/mL Other experiments: 0, 20, 100, and 200 μg/mL	*C. elegans*	Reduces the ROS levels, increases the SOD and CAT activities, and enhances the mRNA levels of cyp35A2, sod-1, sod-3, ctl-2, ctl-3, mtl-1, and mtl-2 genes	Zhang X. et al. (2022)
CPP	*Cyclocarya paliurus*	84,061.23 Da–320.36 Da	No mention	13.75 μg/mL	*C. elegans*	Modulated oxidative stress responses in *C. elegans* by reducing ROS and peroxidation products (MDA, NEFA, GSSG) while enhancing antioxidant enzymes (SOD, CAT, GSH-Px) and GSH levels	Lin et al. (2020)
PAP	*Potentilla anserina L*	No mention	No mention	BALB/C mice: 150, 300, 500 mg/kg Wistar rats: 100, 200, 400 mg/kg	Hypoxia and antiacute hypoxia BALB/C mice acute hypobaric hypoxia-induced Wistar rats	Reduced the levels of MDA and nitric oxide, enhanced the activity of SOD and GSH levels, and reduced brain water content	Shi et al. (2020)
APS	*Astragalus membranaceus*	No mention	No mention	200 mg/kg	SD rats	Increased the contents of CAT and SOD in the brain of SD rats and reduced the content of MDA, reduced the degree of blood viscosity	Sun et al. (2020)
MCP	*Momordica charantia*	No mention	No mention	100, 300 mg/kg	Aging mice induced by d-galactose (D-Gal)	Facilitated the nuclear translocation of Nrf2 and β-catenin, increased antioxidant capacity by reducing MDA and increasing SOD and GSH levels	Yue et al. (2023)
HRPI	*Hippophae rhamnoides L.*	19.138 kDa	Man, Rha, Glc, Gal, and Ara	13 mg/kg·d,26 mg/kg·d,52 mg/kg·d1	AD mice	Suppressed the expression levels of Keap1 and increased those of Nrf2 and antioxidant enzymes SOD and GSH-Px	Zhao H. et al. (2023)
LBP	*Lycium barbarum*	22.355 kDa	Rha, Ara, Gal, Glc, Man, Rib, GlcA	Cells: 7.81, 15.62, 31.25, 62.5, 125, 250 and 500 μg/mL Animals: 50, 100, 200 mg/kg	HT-22 cell and C57BL/6 mice	Reversed the decreases the mRNA and protein expression of Nrf2, NQP1, and HO-1, and increases the mRNA and protein expression of Keap1, elevated the activities of SOD and GSH-Px, and significantly reduced in MDA	Yang et al. (2023)
PSP	*Polygonatum cyrtonema*	6 kD to 14 kD	6 kinds of sugars and uronic acids	200, 400 and 800 mg/kg	C57BL/6 mice	Increased the expression levels of Nrf2 and HO-1 and hippocampal SOD activity and decreased hippocampal MDA content	Xie et al. (2024)

**TABLE 3:** The inhibitory effects of different plant polysaccharides on apoptosis.

Name	Polysaccharides source	Average Mw	Monosaccharide compositions	Dose	Experimental model	Involved mechanism	Ref
CAP	*Chinese angelica*	No mention	No mention	10, 100, 200 μg/mL	LPS-induced PC12 cell	Downregulated COX-1, thereby activating the PI3K/AKT signaling pathway, increasing Bcl-2 levels, decreasing Bax, cleaved-CASP3 and cleaved-CASP9, and reducing mRNA/protein expression of IL-1β, IL-6, IL-8, and TNF-α	Xie et al. (2018)
ASP	*Angelica sinensis*	No mention	No mention	10 mg/kg	CIRI rats	Activated the PI3K/AKT pathway, upregulated the expression of Bcl-2 protein in activated cells, and inhibited the expression of cleaved-CASP3 and Bax	Xu H. et al. (2021)
BRP	*Brassica rapa*	No mention	No mention	38, 75, 150 mg/kg/d	SD rats	Increased phosphorylation of PI3K and Akt, and further upregulated the expression of HIF-1α, reduced the expression of CASP3 and Bax proteins and increased the levels of Bcl-2	Zou et al. (2022)
LBP	*Lycium barbarum*	No mention	No mention	15, 30 and 60 mg/mL	Primary hippocampal neurons from C57BL/6 mice	Enhanced the expression levels of p-Akt and p-mTOR, downregulated CASP3, as well as upregulated the expression of Bcl-2/Bax and p62	Yu et al. (2018)
PAP	*Potentilla anserine L.*	No mention	No mention	Cells: 6.25 or 12.5 μmol/L Animals: 1.5 mg/kg	N2a and SH-SY5Y cell and BALB/c mice	Increased mitochondrial membrane potential, inhibited the release of CytC, and prevented the cleavage of CASP3 and PARP, as well as restrained activation of the AKT/mTOR pathway	Cheng et al. (2022)
MP	*Lepidium meyenii* Walp.	No mention	No mention	Cells: 25, 50 and 100 μg/mL Animals:75, 150, 300 mg/kg	SH-SY5Y cell and D-gal-induced ICR mice	Increased GSH-Px activity, reduced MDA levels, and attenuated the cell damage, inhibited apoptosis, relieved cell cycle arrest, and downregulated cleaved CASP3 and P53 protein expression	Zhou et al. (2022)
CCP	*Coptis chinensis*	3.96 kDa	Glu	5, 10, 25, 50, 100 and 200 μg/mL	PC12 cell	Inhibited JNK phosphorylation, mitochondrial dysfunction and CytC release, reversed the increase of Bax and lysed CASP3, and decreased the expression of Bcl-2 protein	Li et al. (2019)
CSH	*Hedysarum polybotrys* Hand-Mazz	No mention	No mention	40 μg/mL salidroside and 20 μg/mL Hedysari Radix polysaccharide	Aβ25-35-induced HT22 cell	Decreased the protein expression levels of Bax, CytC, and cleaved CASP3, and increased the levels of PKCβ, Bcl-2, and p-ERK1/2	Yang et al. (2022)
ASP	*Angelica sinensis*	No mention	No mention	50 mg/kg	SD rats	Activated the BDNF/TrkB/CREB pathway, reduced the expression of CASP3 and Bax and increased that of Bcl-2 and the ratio of Bcl-2/Bax	Du et al. (2020)
DP	*Dendrobium officinale*	No mention	Glc, Man, Gal, GalA	200 mg/kg	C57BL/6J mice	Improved the phosphorylation of AMPK and mitochondrial function, promoted DNA demethylation and changed the epigenetic state of DNA, regulated TET2 function, inhibited the expression of Bax, Bak, and the ratio of cleaved CASP3/CASP3, increased the expression of anti-apoptotic proteins, Bcl-2 and the ratio of Bcl-2/Bax	Chen et al. (2023)
PPV-6	*Basella alba*	134.14 kDa	Glc, Gal, Ara, Rha, Gal-UA, Glc-UA	250 μg/mL	Primary cortical neuron from SD rats	Downregulated SHH expression, inhibit CCR in neurons and subsequent apoptosis	Hou et al. (2024)
LBP	*Lycium barbarum*	No mention	No mention	Cells: 100 mg/L Animals: 20 mg/kg	Primary cortical neuron from Wistar rats and Wister rats	Inhibited the decrease in levels of NR2A, pAkt and pCREB proteins, and antagonized increase in expression of major proteins in the NR2B signal pathway including NR2B, nNOS, BAD, CytC and cleaved CASP3, and also reduced ROS level, calcium influx and mitochondrial permeability	Shi et al. (2017)
CYP	*Corydalis yanhusuo*	75.5 kDa	Glc	5, 10, 25, 50, 100 and 200 μg/mL	PC12 cell	Reduced the expression ratio of Bax/Bcl2 and decreased the expression of the cleaved CASP8 and cleaved CASP9 and cleaved CASP3 proteins	He et al. (2020)
CPPs	*Codonopsis pilosula*	No mention	No mention	75, 150, 300 mg/kg	APP/PS1 specific pathogen-free mice	Promoted the morphological recovery of the endoplasmic reticulum, reduced the expression levels of GRP78, ATF4 and CHOP, and decreased the ratio of p-PERK to total PERK	Cai et al. (2025)
PCP	*Polygonatum cyrtonema*	8.5 kDa	No mention	60 and 100 mg/kg	C57BL/6 mice	Reversed the upregulation of ATF6, BIP, CHOP, p-IRE1α, p-EIF2α and XBP-1s, reduced the expression levels of Bax and cleaved CASP3, and increased the expression of Bcl-2	Li Q. M. et al. (2025)

## 3 Structural determinants of polysaccharide neuroactivity

The neuroprotective and immunomodulatory activities of polysaccharides are fundamentally dictated by their structural characteristics, encompassing molecular weight (Mw), monosaccharide composition, glycosidic linkage configurations, branching complexity, and discrete chemical modifications. The subsequent subsections provide a detailed, evidence-based exposition of these structure–activity relationships to elucidate how specific structural determinants govern the biological functions and mechanistic actions of polysaccharides Supplementary Table 1.

Molecular Weight: Mw critically influences polysaccharide solubility, viscosity, tissue diffusion, and epitope exposure (Peng et al., 2023; Zhang et al., 2020). Polysaccharides with Mw in the range of 10–100 kDa generally exhibit favorable diffusion and accessibility (Wilkowska et al., 2024), enabling interactions with neural and immune receptors and partial absorption or hydrolysis into bioactive fragments. For example, *Gastrodia elata* Blume polysaccharide (∼12 kDa) attenuated proinflammatory cytokines in cerebral ischemia models (Zhang et al., 2024), while *Lycium barbarum* L. polysaccharide (∼22 kDa) reduced oxidative stress in light-induced neuronal injury (Yang et al., 2023). These findings are consistent with the notion that Mw values in this range optimize solubility, diffusion efficiency, thereby enabling neuroprotective actions. Conversely, high Mw polysaccharides (>700 kDa) often display limited permeability and exert neuroprotective effects indirectly via gut microbiota modulation, systemic metabolism, or immune regulation, as exemplified by *Dendrobium officinale* Kimura and Migo polysaccharide improving cognitive function through microbiota-derived metabolites (Curcio et al., 2020; Wolak and Throne, 2013).

Monosaccharide Composition: The immunomodulatory and neuroprotective activities of polysaccharides are profoundly influenced by their monosaccharide composition, because the identity and proportion of constituent sugars determine charge density, backbone rigidity, branching patterns and the spatial presentation of receptor-recognition motifs. Acidic residues (e.g., galacturonic acid) enhance solubility and protein interactions, whereas mannose or rhamnose residzues engage C-type lectins or pattern-recognition receptors. The high arabinose content, particularly within branched architectures such as RP01-1, may impart a distinct spatial conformation and greater hydrophilicity. These structural features could enhance interactions with neuronal surface receptors (e.g., TrkB and integrins) and facilitate activation of downstream neurotrophic signaling pathways, including BDNF/AKT/ERK/CREB (Zeng et al., 2020). Glucose-rich polysaccharides such as GEP, which contain α-glucopyranose backbones with mixed α-(1→4), α-(1→6), and β-(1→6) linkages, exhibit repetitive structural motifs that may favor recognition by pattern-recognition receptors including toll-like receptor 4 (TLR4). These interactions are thought to influence nuclear factor kappa-light-chain-enhancer of activated B cells (NF-κB)–related signaling and subsequently modulate pro-inflammatory mediator expression (Gan et al., 2024). Stoichiometric ratios influence conformational exposure, dictating epitope accessibility for immune, epithelial, or neural receptors (Ishaq et al., 2025).

Glycosidic Linkages and Backbone: α- or β-linked backbones and pyranose/furanose configurations govern rigidity, solubility, and multivalent receptor-binding potential. Polysaccharides with defined β-linked architectures, such as the β-D-Galp backbone containing →3- and →6-linkages in *Acorus tatarinowii* Schott ATP50-3 (Zhong et al., 2020), tend to adopt stable helical conformations. These structural features facilitate recognition by glial pattern recognition receptors including TLR4 and are associated with downstream modulation of NF-κB signaling and enhancement of endogenous antioxidant responses, collectively contributing to reduced neuroinflammation and oxidative stress. α-linked domains increase flexibility, exposing functional epitopes and facilitating enzymatic hydrolysis into smaller bioactive fragments (Rumon et al., 2024). Mixed α/β linkages enable simultaneous engagement of multiple signaling pathways, broadening neuroprotective scope (Ikeshima-Kataoka et al., 2022).

Branching Degree: High branch density enhances solubility, stabilizes three-dimensional conformations, and exposes multiple functional residues, facilitating multivalent interactions with neuronal, glial, endothelial, and immune receptors. Branched architectures are associated with anti-inflammatory activity, antioxidant effects, protection against Aβ-induced apoptosis, and promotion of neurite outgrowth. For example, ATP50-3, featuring branches at the C-3 and C-6 positions of the backbone, potently suppresses neuroinflammation (Zhong et al., 2020). ARP-1 from *Aster tataricus* L.f., containing →3,6)-β-D-Galp-(1→ branched residues, demonstrates significant antioxidant activity at 0.2–2.0 mg/mL by reducing reactive oxygen species and increasing SOD levels (Zhu et al., 2022). The functional relevance of branching is further exemplified by acetylated JCS1 (YJCS1), which carries branches at the C-6 position of (1→4)-linked α-Glcp and, as a result, induces neurite outgrowth at 5.56 μM, whereas the less-branched native JCS1 is inactive (Jin et al., 2017).

Chemical Modifications: Acetylation, sulfation, phosphorylation, and incorporation of elements such as selenium enhance solubility, conformational stability, enzymatic resistance, and receptor engagement. Acetylation can increase hydrophobicity locally, stabilize bioactive conformations, and facilitate blood-brain barrier penetration. Selenium or carboxyl groups contribute electron-rich centers or ionic interactions, promoting radical scavenging and neuroprotective signaling (Sheng et al., 2017; Hu Z. et al., 2020).

These critical structural features not only determine the bioactivity of polysaccharides but also profoundly influence their pharmacokinetic behavior in reaching and engaging the CNS. Therefore, a comprehensive understanding of their structure–function relationships must contextualize these molecular properties within the framework of their interactions with the blood-brain barrier. Although plant-derived polysaccharides are typically characterized by strong hydrophilicity, accumulating evidence demonstrates that they can modulate CNS function through three interrelated mechanisms operating at the interface of the blood-brain barrier (BBB).

First, restricted direct entry into the CNS may occur for low-molecular-weight polysaccharides or for enzymatic and microbiota-derived fragments via carrier-mediated transport or adsorptive-mediated transcytosis, with involvement of endothelial surface interactions (Han et al., 2014). Pathological BBB perturbation may further permit translocation, as shown in ischemic models in which *Momordica charantia* polysaccharides traverse the BBB (Hu Z. et al., 2020), although such direct routes account for only a minor component of their neuroprotective activity.

Second, polysaccharides exert indirect regulatory effects by modulating exosome biogenesis, cargo loading, and release from neural, immune, and stem cells (Chen et al., 2025; Jones et al., 2021; Zhao Y. et al., 2023; Clos-Sansalvador et al., 2022; Kim et al., 2024; Lee et al., 2021). These exosomes readily cross the BBB and deliver functional cargos, exemplified by Lycium barbarum polysaccharide–conditioned neural stem cell exosomes transferring miR-133a-3p to neurons and enhancing CNS repair (Li et al., 2023).

Third, the gut–brain axis represents the primary pathway. Microbial fermentation of polysaccharides produces neuroactive metabolites, especially short-chain fatty acids and tryptophan derivatives, which cross the BBB to modulate microglial activation, neuroinflammation, and synaptic plasticity. Dendrobium officinale and Corydalis polysaccharides exert effects via SCFA-mediated mechanisms (Sun et al., 2022; Fang et al., 2023), while Astragalus polysaccharides enhance BBB integrity and reduce systemic inflammation, indirectly promoting neuroprotection (Jia et al., 2022).

Collectively, these findings suggest that plant polysaccharides primarily influence CNS function via microbiota-derived metabolites and exosome-mediated signaling, whereas direct translocation across the BBB contributes only marginally.

## 4 Mechanisms of neuroprotection by plant polysaccharides

### 4.1 Blockade of neuroinflammation

**FIGURE 2:**
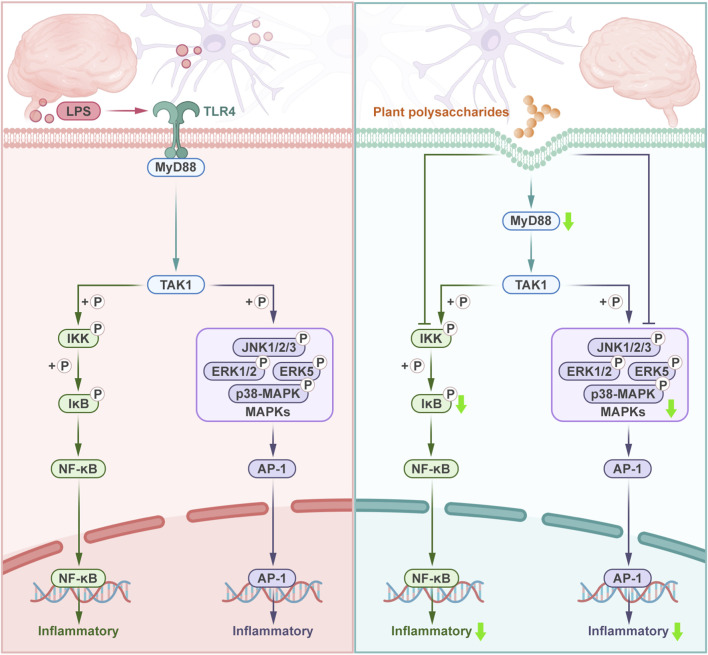
Plant polysaccharides alleviate the process of neuroinflammation through NF-κB and MAPK signaling pathway. Under pathological conditions, NF-κB and MAPK signaling pathway are activated, leading to the upregulation of neuronal inflammation. After administration of plant polysaccharides, the signaling pathway was inhibited.

Neuroinflammation is a multifaceted biological response that serves both protective and detrimental functions in the pathophysiology of the CNS. While transient neuroinflammatory processes exert a positive effect on maintaining cerebral homeostasis and initiating repair mechanisms, persistent or excessive activation of these pathways can result in neuronal dysfunction and structural damage (Sochocka et al., 2017). This inflammatory response is implicated in the pathogenesis of CNS disorders, including AD, epilepsy, high-altitude cerebral edema, and cerebral ischemic injury. Consequently, the regulation of neuroinflammation has emerged as an effective therapeutic strategy for CNS disorders. Mechanistic research further indicates that various plant polysaccharides exert anti-neuroinflammatory effects by multitarget modulation of key signaling pathways, such as NF-κB and mitogen-activated protein kinase (MAPK). Figure 2 shows the process by which plant polysaccharides alleviate neuroinflammation through these two signaling pathways, while Table 1 systematically summarizes the anti-neuroinflammatory efficacy of diverse plant polysaccharides across experimental models.

NF-κB plays a pivotal regulatory role in the pathogenesis of neuroinflammation. Under physiological conditions, NF-κB is retained in the cytoplasm in an inactive state through binding to its inhibitory protein inhibitor of kappa B (IκB) (Napetschnig and Wu, 2013). During inflammatory responses, NF-κB is activated by upstream signaling molecules. Activation of pattern recognition receptors, such as TLR4 which recruits myeloid differentiation primary response 88 (MyD88), ultimately activates the IκB kinase (IKK) complex (Napetschnig and Wu, 2013). The IKK complex phosphorylates IκB, leading to its ubiquitination and proteasomal degradation, thereby enabling the NF-κB to be translocated to the nucleus in the absence of IκB (Anilkumar and Wright-Jin, 2024). Within the nucleus, NF-κB binds to κB consensus sequences in the promoter regions of proinflammatory genes, driving the expression of mediators, such as tumor necrosis factor α (TNF-α), interleukin-6 (IL-6), and interleukin-1β (IL-1β) (Zhao B. et al., 2021). These mediators directly damage neural tissues and amplify inflammatory signaling by activating their corresponding receptors. This process creates a self-reinforcing feedback loop that perpetuates NF-κB activation, thereby sustaining chronic neuroinflammation and aggravating disease progression.

By regulating the NF-κB pathway, plant polysaccharides affect the inflammatory response and exert a neuroprotective effect. *Polygala tenuifolia* Willd polysaccharide modulated the mRNA levels of TLR4 and its adapter molecule MyD88, and suppressed the activation of NF-κB signaling pathway. This led to downregulation of the expression of proinflammatory mediators, such as TNF-α, IL-6, IL-1β, cyclooxygenase-2 (COX-2), and inducible nitric oxide synthase (iNOS) in lipopolysaccharide (LPS)-damaged BV2 microglial cells and neuroinflammation-injured primary cortical neurons (Chen H. et al., 2022). *Acorus tatarinowii* Schott polysaccharide exerts significant anti-neuroinflammatory effects by inactivating the TLR4-mediated MyD88/NF-κB and phosphatidylinositol 3 kinase/protein kinase B (PI3K/AKT) signaling pathways through decreasing the protein levels of TLR4 and MyD88, blocking NF-κB activation cascade phosphorylation and p65 nuclear translocation, and partially mediating the PI3K/AKT pathway to suppress LPS-induced neuroinflammation in BV2 cells. These effects inhibit inflammatory mediators, such as TNF-α, IL-6, IL-1β, COX-2, iNOS, and reduce microglial overactivation (Zhong et al., 2020). Treatment with *Astragalus* polysaccharides effectively suppressed the activity of the key molecule TLR4, as well as reduced the expression levels of NF-κB p65 and phosphorylated IκBα (p-IκBα) proteins, thereby ameliorating neuroinflammation, epileptogenesis, and cognitive impairment in a pentylenetetrazole-induced kindling mouse model (Lu et al., 2024). *Aronia melanocarpa* (Michx.) Elliott polysaccharide attenuated NF-κB overexpression by suppressing the upregulation of its upstream regulator, p-IκBα, which ameliorated D-galactose-induced aging symptoms in CNS through anti-inflammatory mechanisms (Zhao Y. et al., 2021). Sea buckthorn (*Hippophae rhamnoides* L.) polysaccharide can reverse NF-κB phosphorylation and the increase in the expression of iNOS and ionized calcium binding adaptor molecule 1 (IBA-1), a marker of microglia. In addition, the mRNA levels of IL-1β, IL-6, and COX-2 were inhibited in the brain of high-fat diet-fed mice, and the cognitive dysfunction of the mice was alleviated (Lan et al., 2023). Hypoxia-inducible factor-1α (HIF-1α) is an important signaling molecule that mediates the cellular response to hypoxia by inducing the expression of adaptive gene products, and its activation is an important signal for the hypoxic response in tissues (Zou et al., 2022). *Potentilla anserina* L. polysaccharide blocks the activation of NF-κB and HIF-1α signaling pathways. Moreover, it inhibits the production of downstream proinflammatory cytokines such as IL-1β, IL-6, TNF-α, and vascular endothelial growth factor (VEGF), which is significant for the treatment of high-altitude cerebral edema (Shi et al., 2020).

MAPK signaling is a conserved tertiary enzymatic cascade that mediates the transduction of signals from the cell surface to the nucleus through phosphorylation events (Guo et al., 2024). Upon TLR activation, the downstream kinase TGF-beta activated kinase 1 (TAK1) is phosphorylated, serving as a critical node that simultaneously initiates both NF-κB and MAPK signaling pathways (Guo et al., 2024). In addition, MAPKs are comprised of four distinct cascades: extracellular signal-regulated kinase 1/2 (ERK1/2); c-Jun N-terminal kinase (JNK1/2/3); p38-MAPK; and ERK5 (Sun et al., 2015). The MAPK signaling pathway subsequently activates the downstream transcription factor, i.e., activator protein 1 (AP-1), thereby enhancing the release of proinflammatory cytokines including TNF-α, IL-1β, and IL-6 (Kim SY et al., 2025). Previous studies have shown that many macromolecules of biological origin inhibit neuroinflammation and achieve neuroprotective effects through the MAPK pathway (Wang L. et al., 2024; Jeong et al., 2019).

Plant polysaccharides can also inhibit CNS inflammation through the MAPK pathway. SNP2-A, a *Scrophularia ningpoensis* Hemsl. polysaccharide, can reverse the obvious increase of JNK and p38-MAPK protein expression, as well as the obvious decrease of ERK protein expression in cerebral ischemia-reperfusion injury rats. Furthermore, it can inhibit the excessive production of inflammatory cytokines, such as TNF-α and IL-1β, by ameliorating the MAPK pathway (Ma et al., 2019). In mice with focal cerebral ischemic injury induced by middle cerebral artery occlusion, *L. barbarum* polysaccharide inhibited the expression and activation of p38-MAPK and NF-κB in the hippocampus, which exerted functional recovery of memory and motor coordination deficits and neuroprotective effect against cerebral ischemic injury (Zhao et al., 2017). By decreasing p38-MAPK, JNK, ERK phosphorylation, and NF-κB nuclear displacement, *Schisandra chinensis* polysaccharide could downregulate the expression of proinflammatory cytokines (e.g., TNF-α, IL-1β, and IL-6) and the activation of glial cells in the hippocampus, thus improving the cognition of AD mice and showing its therapeutic effect of AD (Xu et al., 2019). Recent studies have shown that pretreatment with *Astragalus mongholicus* Bunge polysaccharide significantly attenuated the phosphorylation of key kinases of the MAPK and NF-κB signaling pathways (e.g., JNK1/2, ERK1/2, p38-MAPK, IκBα, and NF-κB), as well as reduced the levels of proinflammatory factors (e.g., IL-1β, TNF-α, and IL-6) in the hippocampus and cortex of mice (Liu D. et al., 2025).

Furthermore, in the CNS, under different conditions, microglia can differentiate into M1 type with proinflammatory activity and M2 type with anti-inflammatory activity (Tang and Le, 2016). Following the activation of microglia, the M1 type cells trigger the synthesis and release of a cascade of proinflammatory mediators, which ultimately leads to neuronal morphological changes and functional damage (Isik et al., 2023; Liu et al., 2020). Moreover, the M2 type cells showed anti-inflammatory effects by secreting anti-inflammatory factors and other mediators (Isik et al., 2023; Liu et al., 2020). In recent research, plant polysaccharides have emerged as particularly promising candidates for such immunomodulatory interventions. For instance, *Astragalus* polysaccharides demonstrate significant cerebroprotective effects in acute ischemic stroke rats through selective enhancement of M2 polarization via regulation of the ATP/P2X7R signaling axis, which is an important signaling mechanism for neuron-glial communications (Jia et al., 2022). *Schisandra chinensis* (Turcz.) Baill. is often used in traditional and ethnic medicine for amnesia and aging. In BV2 cells, *S. chinensis* polysaccharide suppressed M1 polarization to decrease neuroinflammation by inhibiting the activation of the overactive NF-κB and JNK pathways (Xu et al., 2020). In addition to their role in modulating microglial polarization, plant polysaccharides also show therapeutic potential in the repair of optic nerve injury. In the context of visual prostheses, where electrical stimulation can induce microglial damage, *L. barbarum* polysaccharide confers neuroprotection against bipolar pulse current induced injury. This protective effect is mediated through the enhancement of autophagic activity and the modulation of the MAPK signaling pathway, thereby alleviating neuroinflammation, oxidative stress, and apoptosis (Bie et al., 2015).

Plant polysaccharides mitigate neuroinflammation by modulating NF-κB and MAPK signaling and reducing microglial overactivation, thereby preserving neuronal function *in vitro* and *in vivo*. However, many studies rely on single inflammatory models and do not identify upstream receptors, which limits mechanistic interpretation. Therefore, future research should validate these anti-inflammatory effects across multiple models, employ causal manipulations of upstream pathways, and examine how structural features of polysaccharides relate to their bioactivity.

### 4.2 Inhibition of oxidative stress

Oxidative stress, defined as an imbalance between the production of reactive oxygen species (ROS) and the capacity of the antioxidant defense system, is a critical contributor to cellular and tissue damage (Raguraman et al., 2019). Including oxygen free radicals, such as superoxide anion radicals and hydroxyl radicals, together with hydrogen peroxide (H_2_O_2_), ROS are inevitably generated as byproducts during mitochondrial respiration in the human body (Amoroso et al., 2023). With a dual role in biological systems, ROS function as critical signaling molecules in the modulation of various physiological functions at moderate levels, participating in immune reaction (Liu et al., 2023). However, excessive ROS accumulation disrupts cellular homeostasis by oxidizing key biomolecules (e.g., lipids, proteins, and nucleic acids), ultimately compromising cellular function and viability (Valko et al., 2007). One of the primary consequences of ROS-induced oxidative stress is lipid peroxidation, a chain reaction that targets polyunsaturated fatty acids in cellular membranes. This process generates toxic byproducts, including malondialdehyde (MDA), which serves as a critical biomarker of oxidative damage (Liao et al., 2022). MDA is highly reactive and can form adducts with proteins and DNA, leading to structural modifications, impaired function, and exacerbation of cellular dysfunction (Haro Girón et al., 2023). The progressive accumulation of MDA is correlated to the pathogenesis of various oxidative stress-related diseases, including neurodegeneration (Sultana et al., 2013), cardiovascular disorders (Rehman et al., 2022), and cancer (Kaba et al., 2024). Oxidative stress promotes lipid peroxidation and leads to the compromise of cell membrane integrity. As a cytoplasmic enzyme, lactate dehydrogenase (LDH) is typically confined within the cell under normal conditions. However, oxidative damage to the plasma membrane results in LDH leakage into the extracellular space; thus, it is an established marker of cell membrane injury and cell death (Liao et al., 2022). Elevated LDH levels often correlate with increased ROS accumulation and MDA production, reinforcing the intricate relationship between ROS-induced lipid peroxidation and cellular damage.

The negative effects of oxidative stress on cells and tissues, which is considered to be one of the key factors in the progression of many diseases, have attracted considerable attention in recent years (Zhuang et al., 2018; Zhang F. et al., 2021; Yang S. et al., 2021). Neuronal cells are particularly vulnerable to oxidative damage due to their high oxygen consumption, weak antioxidant defenses, and high levels of polyunsaturated fatty acids in their membranes (Franzoni et al., 2021). Oxidative stress is also a common pathological feature of numerous neurological diseases (Carvalho et al., 2017). It causes lipid oxidation, thereby promoting the production of amyloid-beta peptide (Aβ), which is closely associated with the pathogenesis of AD (Theiss et al., 2022). In ischemic stroke and cerebral ischemia-reperfusion injury, the transient interruption and subsequent restoration of local cerebral arterial blood flow lead to an acute surge of ROS that exceeds the antioxidant capacity of the body. This process initiates DNA damage, lipid peroxidation, BBB disruption, neuron apoptosis, autophagy, and irreversible neurological dysfunction (Li Y. et al., 2025; Akhtar et al., 2025; Wang J. et al., 2025). Additionally, the sudden surge of ROS disrupts cellular homeostasis and activates inflammatory cytokines, thereby amplifying tissue damage, exacerbating the permeability of the BBB, and further intensifying brain injury (Akhtar et al., 2025). Exposure to hypobaric hypoxia significantly increases ROS production and disrupts the cellular antioxidant defense system, compromising both endogenous enzymatic components, including superoxide dismutase (SOD), catalase (CAT), and glutathione peroxidase (GSH-Px), and non-enzymatic antioxidants such as glutathione (GSH). These effects trigger lipid peroxidation, protein oxidation, and DNA damage, ultimately leading to cellular injury and high-altitude cerebral edema (Jing et al., 2022). Therefore, oxidative stress contributes to the pathogenesis of neurological disorders.

**FIGURE 3:**
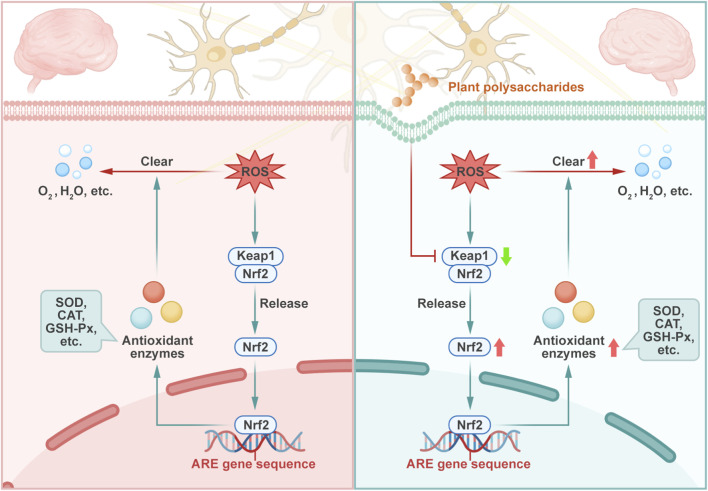
Plant polysaccharides reduce neuronal oxidative stress through Nrf2 signaling pathway. Under pathological conditions, there is an imbalance between the production of reactive oxygen species (ROS) and the defense capacity of antioxidant enzymes. Plant polysaccharides promote the generation of antioxidant enzymes within neuronal cells by facilitating the Nrf2 signaling pathway.

Plant polysaccharides have exhibited antioxidant properties with significant therapeutic potential across diverse diseases (Yang S. et al., 2021; Mu et al., 2023). Emerging evidence indicates that their neuroprotective effects are mediated through inhibition of oxidative stress. Polysaccharide from *A. tataricus* L.f. exhibit significant antioxidant activities, including the scavenging and reducing capacities of 2,2′-azino-bis(3-ethylbenzothiazoline-6-sulfonic acid) (ABTS), hydroxyl, and 2,2-diphenyl-1-picrylhydrazyl (DPPH) free radicals. These are used as an evaluation index for assessing the scavenging activity of natural antioxidants. Moreover, they mitigate ROS and MDA production, thereby protecting PC12 cells from H_2_O_2_-induced oxidative stress (Zhu et al., 2022). *Caenorhabditis elegans* (*C. elegans*) is an important animal model for fundamental biological mechanisms including aging, oxidative stress, and neural function. In the *C. elegans* polyglutamine model, *Epimedium brevicornu* Maxim. polysaccharides effectively scavenge hydroxyl radical, DPPH, and other free radicals, thus reducing ROS levels, decreasing lipid peroxidation, and improving antioxidant enzyme activity. These effects contribute to reduced oxidative stress and the attenuation of polyglutamine-induced neurotoxicity (Xiang et al., 2017). Antioxidant enzymes maintain intracellular homeostasis through a cascade of catalytic reactions. The activities of these enzymes directly determine the ROS scavenging efficiency, and their functional defects will lead to ROS accumulation and trigger oxidative stress events, such as lipid peroxidation and DNA damage. Among antioxidant enzymes, SOD serves as a primary defense mechanism by removing superoxide anion free radicals and converting superoxide anion free radicals into H_2_O_2_ via a reduction reaction (Zheng et al., 2023). Subsequently, CAT and GSH-Px catalyze the decomposition of H_2_O_2_ into water and molecular oxygen, effectively neutralizing ROS and preventing oxidative damage. The coordinated interaction among SOD, CAT, and GSH-Px ensures the rapid elimination of ROS, thereby disrupting the chain propagation of free radical-mediated cellular damage (Shen et al., 2023; Liao et al., 2022). This antioxidant enzyme system is transcriptionally regulated by the nuclear factor-erythroid factor 2-related factor 2 (Nrf2) signaling pathway, which coordinates stress-induced antioxidant defenses. Under physiological conditions, the transcription factor Nrf2 is held in the cytoplasm by Kelch-like ECH-associated protein 1 (Keap1). Upon oxidative stress, Keap1 undergoes conformational changes, leading to the release and nuclear translocation of Nrf2 (Yang P. M. et al., 2021). Once in the nucleus, Nrf2 binds to the antioxidant response element within the promoter regions of various target genes, thereby inducing their transcriptional activation. These genes encode key antioxidant enzymes including SOD, heme oxygenase 1 (HO-1), NAD(P)H:quinone oxidoreductase-1 (NQO1), GSH reductase (GR), etc., all of which play critical roles in cellular defense against oxidative damage (Yang P. M. et al., 2021; Ahmed et al., 2017; de Vries et al., 2008). Through catalytic activity, these enzymes facilitate the breakdown of heme into carbon monoxide, bilirubin, and free iron, contributing to redox homeostasis. Figure 3 shows the process by which plant polysaccharides enhance the antioxidant capacity of neurons by up-regulating the Nrf2 signaling pathway, while Table 2 systematically summarizes the inhibitory effects of diverse plant polysaccharides on oxidative stress across experimental models.

Plant polysaccharides have been shown to regulate antioxidant enzyme activity, thereby enhancing the cellular defense against oxidative stress. It has been reported that *Platycodon grandiflorus* (Jacq.) A. DC. polysaccharide prevents cell membrane damage by enhancing the activity of SOD following H_2_O_2_ exposure, while inhibiting the release of LDH. Additionally, these polysaccharides mitigate intracellular ROS accumulation, reduce MDA levels, and suppress lipid peroxidation (Sheng et al., 2017). A novel neutral polysaccharide from *L. barbarum* L. can reverse and restore the activities of antioxidant enzymes SOD, CAT, and GSH-Px. This is achieved by eliminating ROS and reducing the expression levels of HIF-1α and VEGF genes to inhibit hypoxia-induced oxidative stress injury and protect PC12 cells from hypoxia-induced apoptosis, which shows great potential in the treatment of ischaemic stroke (Li Y. et al., 2025). And in oxygen glucose deprivation/re-oxygenation (OGD/R) injured PC12 cells, *Cistanche deserticola* Ma polysaccharides reduce intracellular ROS levels while enhancing GSH-Px, CAT, and total antioxidant capacity, by suppressing oxidative stress and modulating the pathway of Parkinson’s disease-associated protein DJ-1, a neuroprotective protein associated with antioxidant properties and mitochondrial translocation, thus also demonstrating neuroprotective efficacy and therapeutic potential for protecting neurons in ischemic stroke (Liu et al., 2018). Intervention with *Polygonatum sibiricum* Redouté polysaccharide extract reduced the ROS levels and increased the SOD and CAT activities, which attenuated behavior deficits and neuronal damage in *C. elegans*, relieving fumonisin B1-induced neurotoxicity (Zhang X. et al., 2022). In the oxidative stress and heat stress *C. elegans* models induced by H_2_O_2_ and paraquat, *Cyclocarya paliurus* (Batalin) Iljinsk. polysaccharide inhibited ROS, MDA, nonesterified fatty acid, and oxidized GSH, and promoted the activities of SOD, CAT, GSH-Px by activating SKiNhead-1 (SKN-1) and heat shock factor-1 (HSF-1), which may co-activate downstream oxidative stress and heat-induced genes (Lin et al., 2020). Polysaccharide extracted from *P. anserina* L. significantly reduce the levels of MDA and nitric oxide, enhance the activity of SOD and GSH levels, and reduce brain water content, thus alleviating acute hypobaric hypoxia-induced brain impairment in BALB/C mice (Shi et al., 2020). Additionally, in H_2_O_2_-induced SH-SY5Y cell and aging mice induced by d-Galactose, maca (*Lepidium meyenii* Walp.) polysaccharide demonstrated neuroprotective effects by increasing GSH-Px activity, decreasing MDA content, minimizing LDH leakage, and reversing H_2_O_2_-induced cell morphological damage (Zhou et al., 2022). Administration of *Astragalus* polysaccharides or their nanoparticles in Sprague-Dawley rats elevated SOD and CAT activity, whereas it decreased the levels of neuron-specific enolase and MDA (Sun et al., 2020). Furthermore, these polysaccharides improve blood rheology and coagulation function in cerebral thrombosis models (Sun et al., 2020).

By affecting the Nrf2 signaling pathways, plant polysaccharides regulate the activity of antioxidant enzymes to exert antioxidant effects. *Momordica charantia* polysaccharide facilitated the nuclear translocation of Nrf2 and β-catenin, which are key indicators of antioxidant signaling pathway activation, thereby increasing antioxidant capacity by reducing MDA and increasing SOD and GSH levels (Yue et al., 2023). *Hippophae rhamnoides* L. polysaccharide exerted antioxidative effects by suppressing the expression of Keap1 and increasing those of Nrf2 and antioxidant enzymes SOD and GSH-Px in AD mice (Zhao H. et al., 2023). Lycium barbarum polysaccharide contributed to the reversal of light-induced neurotoxicity by activating the Nrf2/HO-1 pathway and enhancing the antioxidant capacity in mouse hippocampal neurons and HT-22 cells. It effectively reduced the levels of MDA and increased the activity of SOD and GSH-Px, thereby protecting against cell apoptosis and mitochondrial damage (Yang et al., 2023). In single prolonged stress-modeled mice, polysaccharides derived from *Polygonatum cyrtonema* Hua significantly increased the levels of SOD and decreased those of MDA in the hippocampus through the Nrf2/HO-1 pathway. These effects ameliorated synaptic damage and post-traumatic stress disorder of the mice (Xie et al., 2024).

Plant polysaccharides enhance antioxidant defenses by activating Nrf2/HO-1 signaling, reducing ROS and MDA levels, and mitigating lipid peroxidation across neuronal and organismal models. However, most studies assess only downstream oxidative markers without directly confirming causality through pathway-specific interventions, which limits mechanistic interpretation. Therefore, future research should include systematic dose–response studies, employ genetic or pharmacological modulation of redox pathways, and assess bioavailability to substantiate therapeutic relevance.

### 4.3 Inhibition of apoptosis

**FIGURE 4:**
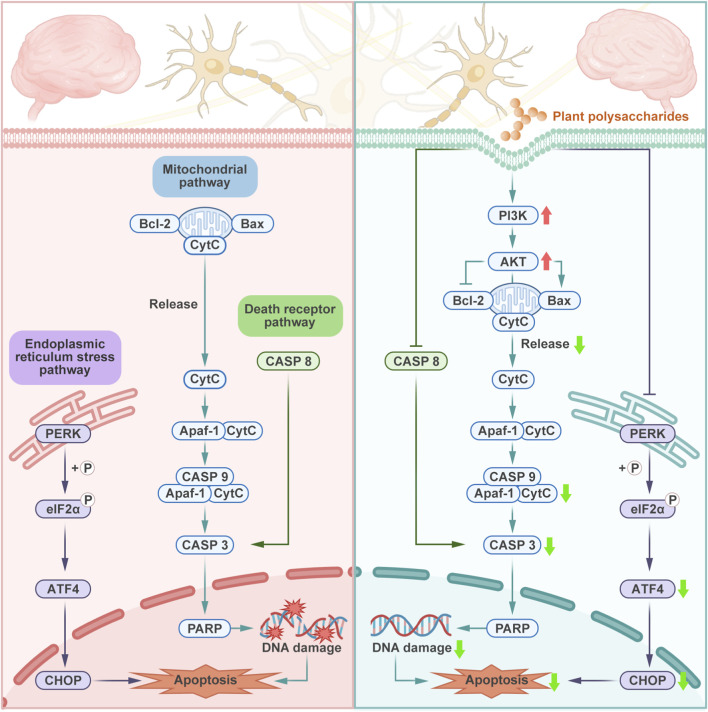
Plant polysaccharides inhibit neuronal apoptosis through the mitochondrial pathway, the death receptor pathway and the endoplasmic reticulum pathway. Plant polysaccharides inhibit apoptosis-related proteins through the PI3K/AKT signaling pathway, maintain the integrity of the mitochondrial membrane, and suppress the release of CytC and the activation of CASP in mitochondria, thereby inhibiting apoptosis. Plant polysaccharides also inhibit apoptosis through the death receptor pathway and by suppressing endoplasmic reticulum stress.

Apoptosis refers to the active death process triggered by the intracellular death cascade induced through specific signals (Sun et al., 2020). In the CNS, the loss of neurons caused by apoptosis affects the normal structure and physiological function. This loss is closely related to CNS disorders, such as PD (Prasertsuksri et al., 2023), ischemic stroke (Xu Z. et al., 2024), and spinal cord injury (Xie et al., 2018). Controlling neuronal apoptosis can be an important strategy for the treatment of neurodegenerative diseases and neuronal loss (Prasertsuksri et al., 2023; Xie et al., 2018; Yang et al., 2022). Figure 4 systematically summarizes the multiple pathways through which plant polysaccharides inhibit neuronal cell apoptosis.

The endogenous pathway, also known as mitochondrial pathway, is a critical mechanism underlying apoptosis, which is triggered by the release of cytochrome-C (CytC) from mitochondria and tightly governed by upstream regulation through the PI3K/AKT signaling cascade. The PI3K/AKT signaling pathway is a highly conserved intracellular cascade that functionally affects key cellular processes, including protein biosynthesis, proliferation, neuronal activation, and morphological changes in different cell types by inhibiting apoptosis and regulating effector molecules, such as apoptosis-related proteins and cell cycle regulators (Sánchez-Alegría et al., 2018; Han et al., 2024; Chang et al., 2018). In the mitochondrial pathway of apoptosis, phosphorylation of PI3K/AKT leads to upregulation of mitochondrial transcription factors and CytC, which plays an important role in mitochondrial energy metabolism (Feng et al., 2020). In the early stages of apoptosis, internal stress causes changes in mitochondrial membrane permeability, and CytC is released from the mitochondrial membrane to initiate the process of cell apoptosis (Luo et al., 2024). After being released into the cytoplasm, CytC binds to apoptosis activation factor 1 (Apaf-1) and spontaneously activates caspase-9 (CASP9) to form the CytC/Apaf-1/CASP9 complex, and activates CASP3 (Feng et al., 2020). CASP3 activates a variety of key substrate proteins, including the poly (ADP-ribose) polymerase, ultimately leading to severe DNA damage and apoptosis (Xu et al., 2023). The regulation of mitochondrial membrane integrity, the release of CytC from mitochondria, and caspase activation are important components of the apoptosis process, highly controlled by the B-cell lymphoma-2 (Bcl-2) family proteins (He et al., 2020). Its members could be classified into two major functional subgroups, namely, prosurvival proteins typified by Bcl-2 and proapoptotic proteins including Bcl-2 antagonist/killer (Bak) and Bcl-2 associated X (Bax) (Czabotar et al., 2014). Upon activation, the proapoptotic proteins undergo conformational changes leading to the formation of mitochondrial outer membrane pores, a pivotal event that results in the release of CytC and other apoptogenic factors from mitochondria (Dudko et al., 2020). Conversely, Bcl-2 protein can inhibit apoptosis by binding to Bcl-2-interacting domain death agonist (Bid), Bcl-2 interacting mediator of cell death (Bim), or Bcl-2-associated death promoter (Bad), and the Bcl-2/Bax ratio determines whether cells survive after receiving apoptotic signals (Cao et al., 2021).

Extensive research confirms that plant polysaccharides regulate key apoptotic mediators of the mitochondrial pathway (e.g., CASP3, Bax, Bcl-2, and p53) through the PI3K/AKT signaling pathway, effectively attenuating neuronal apoptosis. *Angelica* polysaccharide downregulated COX-1, thereby activating the PI3K/AKT signaling pathway and alleviating apoptosis in PC12 cells. This effect was evidenced by a decreased apoptotic cell rate, increased Bcl-2 levels, decreased levels of Bax, cleaved caspase-3, and cleaved caspase-9, suggesting its potential as an effective treatment for spinal cord injury (Xie et al., 2018). Furthermore, *Angelica sinensis* (Oliv.) Diels polysaccharide activated the PI3K/AKT pathway, upregulated the expression of Bcl-2 protein in activated cells, and inhibited the expression of cleaved-CASP3 and Bax. It can effectively improve nerve function and nerve cell apoptosis in cerebral ischemia-reperfusion injury rats (Xu H. et al., 2021). By activating the PI3K/AKT/HIF-1α signaling pathway, *Brassica rapa* L. polysaccharide reduced the expression of CASP3 and Bax proteins and increased the levels of Bcl-2, thereby inhibiting cell apoptosis caused by hypoxia. In addition, polysaccharide also reversed the disorder of pyruvate and glucose metabolism, both of which were clearly protective against acute high-altitude hypoxia-induced brain injury (Zou et al., 2022). Analysis showed that *L. barbarum* polysaccharide downregulated CASP3, as well as upregulated the expression of Bcl-2/Bax and p62 through the PI3K/AKT/mechanistic target of rapamycin kinase (PI3K/AKT/mTOR) signaling pathway, thereby alleviating OGD/R-induced neuronal apoptosis and autophagic cell death in primary hippocampal neurons (Yu et al., 2018). Both mouse and cell experiments showed that *Potentilla anserine* L. polysaccharide can attenuate cadmium chloride-induced neuronal apoptosis. This polysaccharide increased mitochondrial membrane potential, inhibited the release of CytC, and prevented the cleavage of CASP3 and Poly (ADP-ribose) polymerases (PARP), which inhibited the mitochondrial apoptotic pathway, as well as restrained Ca2+/calmodulin-dependent protein kinase II-dependent (Ca2+/CaMKII-dependent) activation of the AKT/mTOR pathway (Cheng et al., 2022). In addition, *L. meyenii* Walp. (maca) polysaccharide relieved cell cycle arrest and downregulated the expression of cleaved CASP3 and p53 protein, key regulators of apoptosis via Bcl-2 family interaction, thus potentially serving as a neuroprotective agent against H_2_O_2_-induced apoptosis (Zhou et al., 2022; Deng et al., 2006).

Besides the PI3K/AKT signaling pathway, plant polysaccharides also regulate other pathways and mechanisms to inhibit apoptosis, including the MAPK signaling pathway, brain derived neurotrophic factor/tropomyosin receptor kinase B/cAMP responsive element binding protein (BDNF/TrkB/CREB) signaling pathway, DNA epigenetics, cell cycle re-entry, and glutamate receptors. JNK and ERK are key components of the MAPK family. The MAPK signaling pathway activates the downstream transcription factor AP-1 to enhance anti-inflammatory responses and regulates diverse cellular processes, including apoptosis (Bu et al., 2021). The neuroprotective effect of *Coptis* polysaccharide is related to JNK-dependent apoptosis. Polysaccharide pretreatment significantly inhibited JNK phosphorylation, LDH release, nuclear fragmentation, mitochondrial dysfunction, and CytC release in PC12 cells induced by Aβ_25-35_; moreover, it reversed the increase of Bax and lysed CASP3, and decreased the expression of Bcl-2 protein, might offer novel approaches for AD prevention and treatment (Li et al., 2019). In Aβ_25-35_-induced HT22 cells, the combination of salidroside and Hedysari Radix polysaccharide, which may be a potential drug for AD, decreased the protein expression levels of Bax, CytC, and cleaved CASP3, whereas it increased those of protein kinase C-β (PKCβ), Bcl-2, and p-ERK1/2 (Yang et al., 2022). Previous studies have shown that CREB could regulate the expression of several protective proteins, including antiapoptotic protein Bcl-2 (Zuo et al., 2016). As an endogenous neurotrophin primarily synthesized in the brain and expressed in the hippocampus and cerebral cortex, BDNF could promote neuronal survival and memory by binding its receptor TrkB, thereby activating BDNF/TrkB signaling to phosphorylate the transcription factor CREB. Through activating the BDNF/TrkB/CREB pathway, *Angelica sinensis* (Oliv.) Diels polysaccharide exerts therapeutic effects on memory impairment in AD rats. It significantly reduced the expression of CASP3 and Bax and increased that of Bcl-2 and the ratio of Bcl-2/Bax, inhibiting the apoptosis of neurons induced by Aβ_25-35_ (Du et al., 2020). DNA epigenetics plays an important regulatory role in synaptic plasticity and cognitive function. *Dendrobium officinale* Kimura and Migo polysaccharide regulates ten eleven translocation dioxygenase 2 (TET2) function by improving the phosphorylation of AMP-activated protein kinase (AMPK) and mitochondrial function, promoting DNA demethylation and changing the epigenetic state of DNA, thus having a preventive effect on diabetes-induced neuronal apoptosis (Chen et al., 2023). As the neurotoxic component in senile plaques of AD, the Aβ toxicity is mediated by the induction of sonic hedgehog (SHH) to trigger cell cycle re-entry (CCR) and apoptosis in post-mitotic neurons (Hou et al., 2024). Polysaccharides from *Basella alba* L. exert protective actions against Aβ neurotoxicity via downregulation of SHH expression to suppress neuronal CCR and subsequent apoptosis. Furthermore, polysaccharides are also capable of directly inhibiting neuronal CCR triggered by the exogenous N-terminal fragment of sonic hedgehog (Hou et al., 2024). The excessive release of glutamate from the presynaptic membrane induced by ischemia leads to excessive activation of glutamate receptors; synaptic NR2A activation stimulates survival pathways, whereas extrasynaptic NR2B upregulation triggers apoptosis (Shi et al., 2017). In OGD-treated cortical neurons, *L. barbarum* polysaccharide prevented the downregulation in the NR2A pathway, including NR2A, p-AKT, and p-CREB, and antagonized the increased expression of major proteins in the NR2B pathway, including NR2B, neuronal nitric oxide synthase (nNOS), Bad, CytC, and cleaved CASP3 (Shi et al., 2017). The dual role of LBP in activating NR2A and inhibiting NR2B signaling indicates that LBP may be a therapeutic candidate for the treatment of ischemic stroke.

While plant polysaccharides mainly regulate apoptosis via the mitochondrial pathway, certain types also mitigate neuronal apoptosis by targeting the exogenous pathway which is also known as death receptor pathway. In the exogenous pathway, CASP8, as the key apoptosis promoter of this pathway in the caspase family, activates the apoptosis effector CASP3 downstream (Tao et al., 2022). Plant polysaccharides could play a neuroprotective role against apoptosis of nerve cells through this pathway. CASP8 is a key enzyme in apoptosis, and its activated form (cleaved CASP8) marks the activation of the death receptor pathway. In Aβ_25-35_-induced PC12 cells, *Corydalis yanhusuo* polysaccharide significantly reversed the expression of Bax/Bcl-2 and significantly decreased the levels of cleaved CASP8, cleaved CASP9, and cleaved CASP3 proteins (He et al., 2020). At least in part, polysaccharides achieve cell protection through the complex regulation of the mitochondrial apoptosis pathway and death receptor pathway.

In addition to the two classical apoptosis pathways mentioned above, the endoplasmic reticulum stress (ERS) pathway represents a recently identified apoptotic mechanism. Disruption of endoplasmic reticulum homeostasis leads to accumulation of misfolded and unfolded proteins, a condition referred to as ERS that compromises cellular homeostasis. As a result, the cell initiates its adaptive mechanism termed the unfolded protein response (UPR) (Xu and Wang, 2024). However, when severe stress exceeds the cellular self-protective capacity through UPR activation, this process ultimately triggers cell apoptosis. Prior studies suggested that inhibiting neuronal apoptosis via the regulation of ERS is a therapeutic target for neuroprotection (Chen Y. et al., 2022). During ERS, protein kinase R-like endoplasmic reticulum kinase (PERK) activates transcription factor activating transcription factor 4 (ATF4) via phosphorylation of eukaryotic translation initiation factor 2α (eIF2α), thereby inducing proapoptotic factor C/EBP homologous protein (CHOP) expression to mediate apoptotic signaling (Yi et al., 2019). Via the PERK/ATF4/CHOP signaling pathway, *Codonopsis pilosula* (Franch.) Nannf. polysaccharides suppress ERS-triggered neuronal apoptosis in APP/PS1 mice. This effect was accompanied by downregulation of glucose-regulated protein 78 (GRP78), PERK, ATF4, CHOP, and Bax expression, and upregulation of Bcl-2 expression (Cai et al., 2025). One hallmark of PD is the accumulation of misfolded proteins in dopaminergic neurons, which induces persistent ERS and prolongs activation of the UPR, ultimately leading to dopaminergic neuron apoptosis. However, in PD mice, *P. cyrtonema* Hua polysaccharide significantly reversed the upregulation of ATF6, binding immunoglobulin protein (BIP), CHOP, phosphorylated inositol-requiring enzyme 1α (p-IRE1α), phosphorylated eukaryotic initiation factor 2α (p-EIF2α), and X-box binding protein 1 spliced (XBP-1s), and by inhibiting ERS, it reduced the expression levels of Bax and cleaved CASP3, while increasing Bcl-2 expression, thereby exerting neuroprotective effects by alleviating apoptosis (Li Q. M. et al., 2025).

Plant polysaccharides inhibit neuronal apoptosis via mitochondrial and death receptor pathways, as well as MAPK and BDNF/TrkB/CREB signaling, targeting key mediators including CASP3, CASP8, Bax, Bcl-2, and p53. Collectively, these multi-pathway anti-apoptotic mechanisms are summarized in Table 3. However, mechanistic evidence is limited due to heterogeneity in apoptotic stimuli and the lack of direct causal verification through pathway-specific interventions. Therefore, future studies should integrate multi-model apoptotic assays, conduct structure–activity analyses, and employ targeted mechanistic interventions to confirm causal pathways.

### 4.4 Regulation of autophagy

Autophagy is an intracellular mechanism that maintains cell homeostasis, and activation of autophagy exerts a protective effect in certain chronic neurodegenerative diseases (Huang et al., 2016; Poels et al., 2012). However, excessive or dysregulated autophagy has also been implicated in neuronal death, particularly in acute neurological diseases such as stroke and hypoxic/ischemic injury (Poels et al., 2012; Puyal et al., 2012). The mTOR serves as a central regulator of autophagy, with its activity being modulated by the upstream PI3K/AKT pathway, which plays a pivotal role in neuronal survival and homeostasis (Heras-Sandoval et al., 2014). By inhibiting the activity of autophagy-related proteins such as beclin 1 (BECN1), mTOR can inhibit autophagy. In contrast, when mTOR is inhibited, the activity of autophagy-related proteins increases and the autophagic process is activated. BECN1, a key component of the autophagy-initiation complex, is recognized as a crucial positive regulator of autophagosome formation (Fekadu and Rami, 2016). Consequently, BECN1 is commonly evaluated in conjunction with other autophagy-related proteins as a key biomarker for assessing autophagic activity and regulatory dynamics. In neuronal autophagy, LC3-I functions as the cytosolic protein form. Upon lipidation, it is converted into LC3-II, which then localizes as a membrane-bound protein to autophagosomes. Both forms are essential for monitoring autophagic activity, with LC3-II specifically reflecting the degradation of cellular components during this process (Pezzini et al., 2023; Tanida et al., 2008).

**FIGURE 5:**
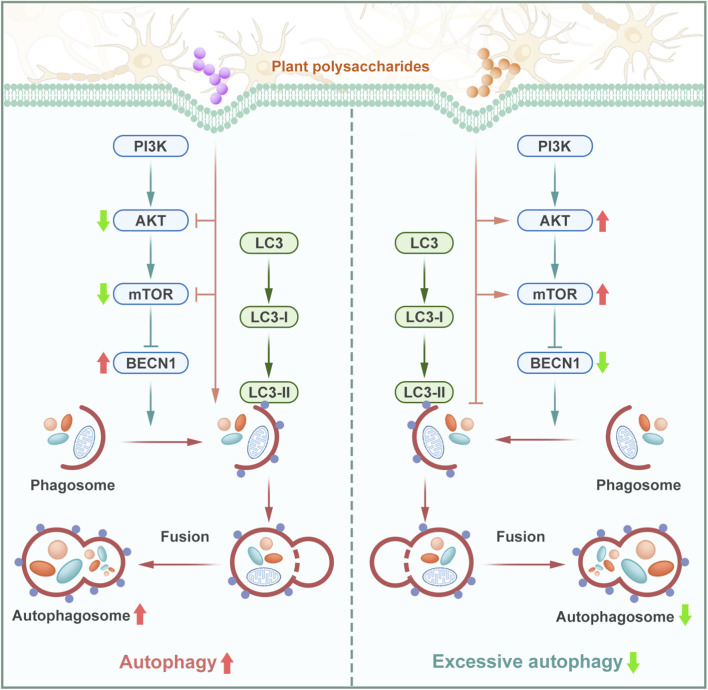
Plant polysaccharides regulate neuronal autophagy through the PI3K/AKT/mTOR signaling pathway. Plant polysaccharides utilize autophagy and alleviate abnormal autophagy through the autophagy central regulatory factor mTOR and the upstream PI3K/AKT signaling pathway, thereby providing neuroprotection.

Therefore, harnessing the protective effect of autophagy while mitigating abnormal autophagy and detrimental effects constitutes a crucial aspect of the neuroprotective mechanisms of plant polysaccharides. Both effects are achieved through the central regulator of autophagy, mTOR, and the upstream PI3K/AKT pathway, within the framework of neuroprotection as shown in Figure 5. It has been shown that plant polysaccharides improve cell viability and autophagy by reducing mTOR activation through the PI3K/AKT pathway: In PC12 cell models, *Astragalus* polysaccharide inhibited the phosphorylation of AKT and its downstream target protein mTOR, and upregulated the expression of the negative regulatory protein PTEN, which facilitates the formation of the autophagosome, and promotes the transformation of LC3-I to LC3-II, the hallmark of autophagy activation (Tan et al., 2020). Similarly, *Lonicera japonica* polysaccharide mitigated neuron loss and disorganized cell arrangement in mice, while significantly alleviating LPS-induced cognitive deficits. These neuroprotective effects may be attributed to the upregulation of autophagy-related proteins, including BECN1, autophagy related 5 (ATG5), vacuolar protein sorting 34 homolog (Vps34), and LC3-II (Wang et al., 2021).

As previously mentioned, plant polysaccharides promote cellular autophagy to exert protective effects. However, they also play a critical regulatory function in mitigating excessive autophagy to prevent autophagy-associated neurotoxicity and apoptosis. It is known that exposure to cadmium induces autophagy. In N2a cells, primary neurons, and the brain of BALB/c mice exposed to cadmium, *P. anserina* L. polysaccharide exerted neuroprotective effects by attenuating cadmium-induced autophagic cell death. This protective mechanism is mediated through the inhibition of the PI3K Class III/BECN1 signaling pathway, highlighting its potential role in modulating autophagy-related neurotoxicity (Cheng et al., 2021). Additionally, it has been shown that *Rehmannia glutinosa* (Gaertn.) Libosch. ex Fisch.and C.A.Mey. polysaccharide activates the AKT/mTOR pathway, thereby reversing light-induced oxidative stress and autophagy in both mice and hippocampal neurons (Yang et al., 2024). Furthermore, *Angelica* polysaccharide alleviated hypoxia-induced apoptosis and autophagy in rat neural stem cell (NSC) by downregulating the expression of Bcl-2 interacting protein 3 (BNIP3) and activating the mTOR and Notch signaling cascades (Xue et al., 2019). Moreover, *L. barbarum* polysaccharide has been particularly noteworthy in mitigating excessive autophagy. *Lycium barbarum* polysaccharide prevents primary hippocampal neuron injury induced by OGD/R through the PI3K/AKT/mTOR pathway. This protective effect is mediated by the downregulation of cleaved CASP3/CASP3, LC3-II/LC3-I and BECN1, coupled with the upregulation of Bcl-2/Bax and p62 (Yu et al., 2018). *Lycium barbarum* polysaccharide has also demonstrated neuroprotective effects in PD models by inhibiting phosphatase and tensin homolog (PTEN), thereby activating the AKT/mTOR pathway, downregulating LC3-II and BECN expression, and reducing excessive autophagy in dopaminergic neurons (Wang et al., 2018). Neural stem cell-derived extracellular vesicles (NSC-EVs) have been recognized as key mediators of post-stroke recovery. Additionally, *L. barbarum* polysaccharide has been shown to enhance the therapeutic potential of NSC-EVs by increasing the enrichment and transfer of miR-133a-3p in NSC-EVs, thereby activating the AMPK/mTOR signaling pathway and inhibiting stroke-induced autophagic activity (Li et al., 2023). These findings further underscore the multifaceted role of plant polysaccharides in regulating autophagy to exert neuroprotective effects, emphasizing their therapeutic potential in both central and peripheral neurodegenerative conditions.

Plant polysaccharides modulate neuronal autophagy primarily through the PI3K/AKT/mTOR pathway, regulating key proteins such as BECN1, LC3-I/II, ATG5, Vps34, BNIP3, and p62 to promote protective autophagy while restraining excessive autophagy. However, current evidence lacks real-time autophagic flux measurements, limiting the interpretation of dynamic autophagy regulation. Therefore, future studies should employ autophagy flux assays, validate findings across multiple models, and apply pathway-specific manipulations to clarify the role of autophagy in neuroprotection.

### 4.5 Regulation of gut microbiota

**FIGURE 6:**
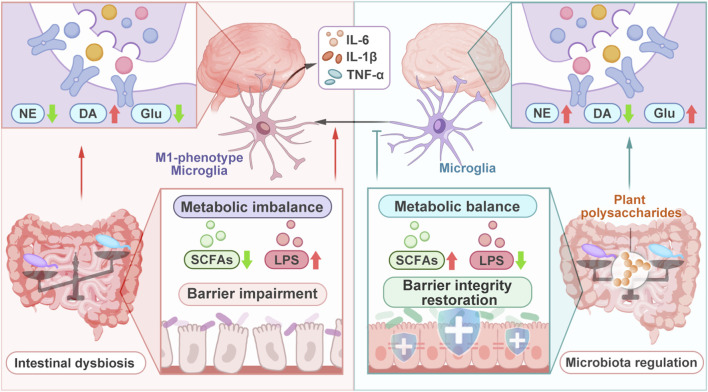
Plant polysaccharides alleviate neuroinflammation and regulate neurotransmitters by regulating the gut microbiota. An imbalance of the gut microbiota can damage the intestinal barrier and promote the entry of toxins into the circulation. And, it significantly alters metabolites, leading to neuroinflammation and neuronal damage. In addition, metabolic imbalance also affect the levels of neurotransmitters. Plant polysaccharides can regulate the composition of the gut microbiota and microbial metabolic products, thereby maintaining the integrity of the barrier and regulating neurotransmitters, exerting neuroprotective effects.

In recent years, accumulating evidence has supported the role of the gut microbiota in modulating brain function and maintaining mammalian health (Lai et al., 2022). The gut microbiota communicates bidirectionally with the CNS through multiple pathways, including immune, endocrine, neural, and metabolic (Qian et al., 2023). However, under pathological conditions, imbalance of gut microbiota may exacerbate neurological pathologies through mechanisms. It disrupts intestinal barrier permeability, allowing toxic substances to translocate into the bloodstream, thereby inducing neuroinflammation. Alterations in gut microbial composition triggered LPS biosynthesis, which subsequently led to mucosal edema and apical villus epithelial cell necrosis (Sun et al., 2022). Gut dysbiosis also downregulates the expression of intestinal epithelial tight junction proteins and reduces mucin secretion. These pathological changes resulted in elevated intestinal lining permeability, ultimately compromising the integrity of the intestinal barrier. This increased permeability facilitates the translocation of endotoxins such as LPS into systemic circulation (Sun et al., 2022). LPS in systemic circulation can activate microglia and astrocytes in the brain and induce M1 polarization of microglia, leading to the upregulation of proinflammatory cytokines, such as TNF-α and IL-6 (Liu D. et al., 2025). This neuroinflammation results in neuronal damage. Moreover, the imbalance significantly alters metabolite profiles, leading to insufficient synthesis of short-chain fatty acids (SCFAs), such as butyrate and propionate. Certain microbial metabolites, including SCFAs, are allowed to cross the BBB, the protective barrier of the CNS, which could lead to inhibition of neuroinflammatory responses in the CNS (Liu D. et al., 2025). In addition, the suppression of neuroinflammation is diminished when synthesis is insufficient. Furthermore, dysbiosis-induced metabolic imbalance may also reduce the absorption of neurotransmitter precursors or alter their metabolism, thereby affecting the levels of central neurotransmitters which are critical for neuronal communication and behavioral regulation (Song Z. et al., 2022). Accordingly, the development of therapeutic strategies aimed at alleviating neuroinflammation through targeted modulation of the gut microbiota has become a pivotal focus in contemporary research. Among these strategies, plant polysaccharides have been reported to modulate gut microbiota composition and microbial metabolites, which may help maintain intestinal barrier integrity and contribute to neuroprotective effects (Figure 6).

By improving intestinal barrier integrity, the systemic influx of harmful toxins was limited, thereby attenuating neuroinflammation. This process represents a critical pathway by which plant polysaccharide-mediated gut microbiota modulation confers neuroprotection. Emerging evidence indicates that *D. officinale* Kimura and Migo polysaccharide may help restore gut microbiota homeostasis disrupted by circadian rhythm disruption in murine models. Specifically, administration of *D. officinale* polysaccharide increased the abundance of beneficial bacterial genera such as *Akkermansia* and *Alistipes* while reducing *Clostridia* levels, which correlated with improvements in intestinal barrier dysfunction through upregulation of tight junction proteins. These changes were linked to lower systemic and CNS LPS levels, reduced NF-κB activation and inflammatory cell infiltration, and mitigated hippocampal neuronal alterations, as well as decreased Aβ deposition (Sun et al., 2022).

Similarly, administration of *S. chinensis* polysaccharides in AD rat models enhanced intestinal barrier function through upregulation of tight junction proteins, mitigated neuroinflammation via suppression of microglial activation, and reversed Aβ_25-35_-induced perturbations in 19 metabolites, predominantly involving linoleic acid, arachidonic acid, and α-linolenic acid metabolism, as well as the tricarboxylic acid cycle, which were correlated with elevating oxyglutamic and succinic acid levels, enhancing energy metabolism, and improving cognitive function (Fu et al., 2023).

The polysaccharide PSP-1 from *P. sibiricum* Redouté restructured the dysregulated gut microbiota in 5xFAD mice by reducing *Helicobacter* and promoting *Akkermansia muciniphila*, which enhanced goblet cell function and tight junction protein expression, decreased intestinal permeability and reduced colonic Aβ deposition. PSP-1 treatment was also linked to increased microglial plaque phagocytosis, which correlated with reductions in cerebral Aβ accumulation and synaptic loss, as well as improvements in memory performance (Luo et al., 2022).

The polysaccharide from sea buckthorn (*H. rhamnoides* L.) restored intestinal homeostasis by reversing the decline of *Ileibacterium* and suppressing the overgrowth of several genera including *Lactobacillus*, *Dubosiella*, *Olsenella*, *Helicobacter*, and *Ruminiclostridium 9.* These changes were associated with upregulation of tight junction proteins OCLN, CLDN1, and ZO-1, reinforcing the intestinal barrier and limit systemic LPS translocation, which in turn attenuated neuroinflammation via inhibition of NF-κB signaling and restored synaptic plasticity through activation of the CREB/BDNF/TrkB pathway (Lan et al., 2023).

Collectively, these findings highlight the potential of plant-derived polysaccharides as promising therapeutic agents for neuroinflammation-related disorders via gut microbiota-mediated mechanisms.

By modulating gut microbiota dysbiosis, these polysaccharides restore metabolic homeostasis, including SCFAs production, which play a crucial role in maintaining neuronal survival. Studies have demonstrated that buckwheat (*Fagopyrum esculentum* Moench) polysaccharides elevate SCFAs levels, particularly acetic acid, propionic acid, and butyric acid, while enriching SCFA-producing taxa, such as *Eubacterium xylanophilum* group, *Lachnospiraceae NK4A136* group, and *Lactobacillus*, which shifts consequently attenuate CNS neuroinflammation and oxidative stress, enhance autophagy, reduce AD-related pathological markers, and ultimately improve cognitive deficits in aluminum trichloride-treated rats (Liu et al., 2024).

Similarly, in PD mice, *G. elata* Blume polysaccharide counteracts gut dysbiosis by modulating key genera including *Akkermansia*, *Lactobacillus*, *Bacteroides*, *Prevotella*, and *Faecalibacterium*. These effects elevate the colonic SCFA content and mitigate mitochondrial apoptosis-driven dopaminergic neuron loss; furthermore, this polysaccharide strengthens intestinal barrier integrity via OCLN upregulation, which correlated with suppression of TLR4/NF-κB pathway in the PD brain and alleviation of neuroinflammation (Gan et al., 2024).

Parallel mechanisms are observed with *A. mongholicus* Bunge polysaccharides, which ameliorate LPS-induced neuroinflammation in mice by modulating gut microbiota composition, such as *Romboutsia*, *Rikenella*, *Dubosiella*, and *Odoribacter*, which restores neuronal homeostasis through five key metabolic pathways (taurine and hypotaurine metabolism, phenylalanine metabolism, pantothenate and coenzyme A biosynthesis, citrate cycle, and propanoate metabolism), inhibiting neuroinflammation (Liu D. et al., 2025).

Gut microbial metabolites, particularly SCFAs, influence neurotransmitter production, a mechanism through which plant polysaccharides modulate neurotransmitter levels to exert neuroprotective effects. Previous studies have found a significant correlation between differential bacterial taxa, SCFAs, and neurotransmitters, which could be key targets in plant polysaccharide-mediated therapies for neurological disorders (Shen et al., 2021; Wu M. et al., 2020; Fang et al., 2023).

For example, *C. yanhusuo* polysaccharides have a major impact on the CNS by influencing the action of SCFAs on the tryptophan-metabolizing enzymes tryptophan hydroxylase 1 (TPH-1) and TPH-2 to affect the formation of 5-hydroxytryptamine. In addition, polysaccharides may also increase the function of norepinephrine and dopamine by influencing SCFAs to regulate the reuptake of norepinephrine and dopamine, and increase BDNF levels to improve the depressive state (Fang et al., 2023).

Moreover, *S. chinensis* polysaccharide reverses imbalances in AD-associated neurotransmitters (e.g., γ-aminobutyric acid, dopamine, and glutamate) by regulating endogenous metabolites (Liu et al., 2019). Among these, *L. barbarum* polysaccharide demonstrates exceptional neurotransmitter-modulating capacity, counteracting environmental pollutant-induced oxidative stress and restoring 5-hydroxytryptamine/cholinergic transmitter balance (Xu Z. et al., 2024), as well as improving dopamine levels and motor function in PD models (Song J. et al., 2022).

Similarly, *Glycyrrhiza uralensis* Fisch. polysaccharide, a key metabolite of antiepileptic botanical drugs, downregulates excitatory neurotransmission by suppressing N-methyl-D-aspartate (NMDA) receptor expression (Yi et al., 2023; Du et al., 2024). The modulation of neurotransmitters by these plant polysaccharides may be due to their effects on gut microbiota and their metabolites. Single-vesicle electrochemical studies on *Glochidion eriocarpum* Champ. ex Benth. polysaccharides have revealed that alternative mechanisms exist, such as direct modulation of calcium influx, membrane lipid remodeling, and exocytosis kinetics to enhance vesicular dopamine release (Cao et al., 2024).

Nevertheless, despite promising findings, current studies on plant polysaccharide mediated neuroprotection present several methodological and mechanistic limitations. Many investigations, including research on Polygonatum sibiricum PSP-1 (Luo et al., 2022) and sea buckthorn SBP (Lan et al., 2023), rely on single animal models and fixed dosing regimens, which limits generalizability and reduces translational relevance. Similarly, studies such as those examining Corydalis yanhusuo CYP (Fang et al., 2023) and Schisandra chinensis SCP (Liu et al., 2019) depend largely on correlative omics analyses without incorporating direct causal experiments, leaving uncertainty regarding whether the observed microbial or metabolite alterations play a necessary or sufficient role in neuroprotection. Even when partial dose-ranging is included, as reported for Astragalus membranaceus APS (Liu D. et al., 2025) and Gastrodia elata GEP (Gan et al., 2024), the proposed microbiota-related mechanisms remain mostly associative. Moreover, structural characterization is often incomplete, particularly with respect to glycosidic linkages, branching patterns, and three-dimensional conformations, which hampers efforts to establish precise structure–function relationships. To address these limitations, future research would benefit from incorporating multi-model validation, systematic dose–response analyses, and experimental approaches capable of directly testing causal links in microbiota-mediated neuroprotection.

### 4.6 Others

In addition to the abovementioned mechanisms, plant polysaccharides exhibit a broad spectrum of neuroprotective effects mediated through multiple molecular pathways. These include the suppression of pyroptosis and ferroptosis, regulation of cell differentiation, enhancement of energy metabolism, and promotion of neural growth. The multifaceted nature of these mechanisms reflects the intricate biological activities of plant polysaccharides. Moreover, The potential efficacy of plant polysaccharides against brain tumors has also been documented. This expanded scope highlights their substantial therapeutic potential and promising prospects for application in the prevention and treatment of neurological disorders.

As a recently discovered programmed cell death pathway, pyroptosis is an inflammatory form of cell death triggered by activated inflammasomes (Li et al., 2021). The NOD-like receptor protein 3 (NLRP3) inflammasome activates and subsequently cleaves Pro-CASP1 into active cleaved CASP1, which processes pro-IL-1β into mature IL-1β, while pore-forming proteins termed gasdermins form cell transmembrane pores to release proinflammatory cytokines and cause lytic cell death (Hu J. J. et al., 2020; Gao et al., 2020). Therefore, controlling the activation of the inflammasome could be a therapeutic target for neurological diseases. Through restraining NLRP3 inflammasome activation and reducing CASP1 expression, *Morinda officinalis* F.C.How polysaccharide can alleviate neuronal death caused by pyroptosis and improve motor dysfunction of PD (Dong et al., 2025).

Ferroptosis, a recently identified iron-dependent, non-apoptotic cell death type, has emerged as a potential key contributor to multiple neurological disorders (Weiland et al., 2019). Characterized by excessive iron accumulation and the lethal production of iron-dependent lipid ROS, ferroptosis initiates a cascade of oxidative stress and lipid peroxidation that damages cellular membranes and disrupts neuronal homeostasis (Nakamura et al., 2019; Chai et al., 2024). Unlike apoptosis, necrosis, or autophagy, ferroptosis exhibits distinct morphological, biochemical, and genetic profiles, positioning it as a unique therapeutic target for nervous system injuries (Chai et al., 2024). Beyond its role in oxidative stress, ferroptosis is increasingly linked to pathological processes such as neuroinflammation and synaptic structural degeneration, both of which are critical drivers of neurodegenerative and cerebrovascular diseases. The ability to modulate ferroptotic pathways offers a promising strategy for mitigating neuronal damage by addressing iron-induced oxidative stress, as well as downstream events, such as lipid peroxidation-mediated membrane damage and inflammatory signaling dysregulation. Polysaccharides have demonstrated significant anti-ferroptotic and neuroprotective properties across various models of neurological injury. Polysaccharide isolated from *Salvia miltiorrhiza* Bunge attenuates OGD/R-induced ferroptosis and lipid peroxidation by activating the Nrf2/HO-1 signaling pathway in PC12 cells, indicating that polysaccharide could inhibit ferroptosis to alleviate oxidative stress injury (Meng et al., 2022). Similarly, a neutral polysaccharide derived from *G. elata* Blume mitigated cerebral ischemia-reperfusion injury by suppressing ferroptosis-mediated neuroinflammation (Zhang et al., 2024) both in an ischemic stroke mouse model and OGD/R-induced HT22 cells. Moreover, in a vascular dementia rat model, which is closely associated with ferroptosis, *Dendrobium nobile* Lindl. polysaccharides preserved synaptic structural integrity and enhanced neuronal function by suppressing ferroptotic processes (Ming et al., 2023). These findings collectively underscore the pivotal role of plant-derived polysaccharides in modulating ferroptosis-related neuropathologies and highlight their therapeutic potential in CNS disorders.

Emerging studies have demonstrated that plant polysaccharides possess the capacity to regulate NSC fate determination, especially promote oligodendrocyte differentiation. Oligodendrocytes, responsible for the formation and maintenance of the myelin sheath, are essential for rapid axonal conduction and neuronal survival in the CNS (Bouchard et al., 2024). *Astragalus* polysaccharides have exhibited notable neuroprotective and remyelination-promoting properties in models of CNS demyelination. Mechanistically, *Astragalus* polysaccharides facilitate NSC lineage specification by suppressing stemness maintenance and astrocytic differentiation, while promoting oligodendrocytic and neuronal differentiation both *in vivo* and *in vitro*. These effects are mediated, at least in part, through the activation of the SHH signaling pathway, thereby enhancing oligodendrogenesis and contributing to myelin repair (Ye et al., 2021). Additionally, *Astragalus* polysaccharides attenuate neuroinflammation, reduce CD8+T cell infiltration into the CNS parenchyma, and promote oligodendrocyte maturation, collectively contributing to their neuroprotective efficacy (Zhao et al., 2024). Moreover, *M. charantia*-derived polysaccharides reprogram NSC differentiation under pathological conditions by shifting cell fate from gliogenic to neurogenic lineages. This process is mediated by the upregulation of sirtuin-1 (SIRT1), which promotes the deacetylation and subsequent nuclear translocation of β-catenin, thereby enhancing neuronal lineage specification (Hu Z. et al., 2020). Collectively, these findings highlight the multifaceted roles of plant polysaccharides in modulating NSC plasticity and promoting remyelination, positioning them as promising therapeutic candidates for repair after neural injury.

Recent studies have further revealed their critical roles of plant polysaccharides in enhancing energy metabolism and supporting neuronal growth and survival. Plant-derived polysaccharides facilitate the repair and regeneration of nerve cells following injury through multiple signaling pathways associated with cellular bioenergetics and neurotrophic support. In a Aβ1-40-induced PC12 cell model, *C. pilosula* (Franch.) Nannf. polysaccharides significantly elevated the intracellular levels of NAD^+^ and the NAD^+^/NADH ratio, as well as upregulated the expression of NAD^+^-dependent deacetylases SIRT1 and SIRT3, along with their downstream effector peroxisome proliferator-activated receptor γ coactivator 1-α (PGC-1α). These observations indicated that *C. pilosula* polysaccharides restore metabolic homeostasis via NAD^+^-related signaling pathways (Hu et al., 2021). *Lycium barbarum* polysaccharide ameliorates behavioral dysfunction and reverses the suppression of the BDNF/TrκB/ERK signaling cascade induced by continuous light exposure in mice (Yang et al., 2023). In addition, a pectin polysaccharide isolated from roots of *P. tenuifolia* Willd promoted neurite outgrowth in PC12 cells and primary cortex neurons via activation of the AKT/ERK/CREB pathway (Zeng et al., 2020).

Brain tumors are characterized by uncontrolled proliferation, angiogenesis, immune evasion, and a complex tumor microenvironment. Despite these challenges, accumulating evidence indicates that plant polysaccharides possess considerable potential to modulate key oncogenic processes, although their high molecular weight may restrict direct interactions with intracellular targets. For instance, in a C6 glioma rat model, *Astragalus* polysaccharide effectively suppressed tumor proliferation and enhanced the therapeutic efficacy of temozolomide. This effect was mediated through the downregulation of proliferative markers such as PCNA and VEGF, upregulation of the differentiation-associated marker GFAP, and improvement of immune function via reduction of immunosuppressive cytokines TGF-β1 and IL-10 (Zhu et al., 2017). In addition to their direct antitumor activity, plant polysaccharides also exhibit radioprotective properties that may help mitigate treatment related complications. Radiation-induced brain injury, which is commonly driven by microglial hyperactivation and neuroinflammation, can be ameliorated by *L. barbarum* polysaccharides. These metabolites suppress the IKKβ/IκBα/NF-κB pathway, thereby promoting microglial polarization toward the anti-inflammatory M2 phenotype and significantly attenuating the production of pro-inflammatory mediators including NO, IL-1β, and TNF-α. These findings illustrate the multifaceted role of plant polysaccharides in simultaneously targeting tumor progression and alleviating the adverse effects associated with conventional therapies, thereby highlighting their potential as complementary agents in neuro-oncology (Zhang et al., 2025).

Plant polysaccharides exhibit broad-spectrum neuroprotective activities by regulating pyroptosis, ferroptosis, neural stem cell differentiation, energy metabolism, and neuronal growth, and additionally demonstrate preliminary anti-tumor effects. These actions are associated with modulation of key molecular mediators, including CASP1, GPX4, SIRT1, PGC-1α, and BDNF. However, the underlying anti-tumor mechanisms of plant polysaccharides remain poorly understood, potentially constrained by their high molecular weight and limited intracellular uptake. Therefore, future research should integrate multi-model validation, structure–activity analyses, targeted mechanistic experiments, and evaluations of pharmacokinetics and CNS bioavailability to clarify their therapeutic potential.

Although the current body of literature provides substantial descriptive evidence supporting the multifaceted neuroprotective effects of plant polysaccharides, these studies are frequently limited by several constraints. Specifically, most investigations rely predominantly on single cell lines or single animal models, often employ fixed or non-optimized dosing regimens, and lack comprehensive structural characterization of the polysaccharides, which hampers the ability to correlate molecular features with bioactivity. Furthermore, the evaluation of pharmacokinetics, CNS bioavailability, and blood–brain barrier permeability is often incomplete or absent, limiting the translational relevance of the findings. Experimental heterogeneity, including variability in disease models, intervention timing, and readout selection, coupled with the sparse use of genetic or pharmacological manipulations, further restricts the capacity to draw robust causal inferences regarding the underlying mechanisms. To address these limitations, future research should integrate multi-model validation, systematic dose–response studies, and detailed structure–activity analyses, as well as implement targeted mechanistic interventions across relevant signaling pathways. In addition, comprehensive pharmacokinetic, CNS bioavailability, and translational assessments should be incorporated to enable a rigorous establishment of causality and to support the therapeutic potential of plant polysaccharides in clinical applications.

## 5 Drug delivery systems of plant polysaccharides

The CNS possesses a highly specialized anatomical structure in which the BBB restricts the entry of nearly all large-molecule drugs and many small-molecule drugs into the brain, presenting a major challenge for the treatment of neurological disorders. As natural biological macromolecules, plant polysaccharides primarily exert most of their CNS related activities through indirect or peripheral pathways. These limitations highlight the urgent need to develop plant polysaccharide based drug delivery systems capable of ecrossing the BBB efficiently and enabling direct pharmacological action within the CNS. In recent years, nanotechnology has emerged as a compelling platform for overcoming the multifaceted diagnostic and therapeutic challenges associated with CNS disorders. Owing to their enhanced capacity for BBB penetration, and versatile surface functionalization, nanocarriers can integrate diverse therapeutic modalities within a single system. These advantages collectively contribute to substantial improvements in drug solubility, stability, and overall pharmacokinetic profiles. *Astragalus* polysaccharide nanoparticles, characterized by their smooth morphology and sustained release profile *in vitro*, have been shown to enhance BBB penetration and drug delivery efficiency, leading to improved therapeutic outcomes in the treatment of cerebral thrombosis in SD rats compared with conventional Astragalus polysaccharides (Sun et al., 2020).

Given this background, natural plant polysaccharides are increasingly being explored as novel nanocarriers. Their functional versatility, strong bioadhesion, and intrinsic biocompatibility make them well suited for CNS targeted applications. A representative example is the NLXT-Nanoparticles (NLXT-NNPs) isolated from the traditional Chinese medicine Naoluo Xintong (NLXT), which are primarily composed of polysaccharides, proteins, and saponins. With a size of approximately 200 nm and a negative surface charge, these nanoparticles carry bioactive metabolites such as Ginsenoside Rg1, Rb1, and Astragaloside IV. These nanoparticles are indispensable to the neuroprotective effects of NLXT, as their removal markedly diminishes its antioxidative stress and anti-apoptotic activities (Zhao et al., 2022). Furthermore, advances in nano-engineering have facilitated the construction of increasingly sophisticated polysaccharide based nanoplatforms. The nanocarrier MAOE@TMP was fabricated by conjugating *Angelica* polysaccharides with ethyl ferulate to form amphiphilic nanoparticles, which were then camouflaged with a macrophage membrane. This design enables effective BBB crossing and ROS-responsive drug release, significantly reducing cerebral infarction volume and improving neurological function in ischemic stroke (Su et al., 2022a). Similarly, the multifunctional bionanoparticle MSAOR@Cur, produced by modifying *Angelica* polysaccharides with sialic acid and followed by macrophage membranes coating, enabled successful delivery of curcumin to ischemic brain regions (Su et al., 2022b). Another biomimetic hybrid nanoplatform termed Exo-Lip, which is formed by fusing neural stem cell derived exosomes with liposomes encapsulating with *Millettia pulchra* (Yulangsan) polysaccharide, alleviates neuroinflammation and restores lipid metabolism in ischemic stroke, thereby reducing infarct volume and promoting functional recovery (Xie et al., 2025).

In addition, across a broad spectrum of CNS disorders, polysaccharide based nanoplatforms exhibit substantial promise in promoting neural repair. For instance, an oriented electrospun polycaprolactone membrane incorporating red ginseng polysaccharides and Fe_3_O_4_ magnetic nanoparticles has been shown to facilitate neural repair by harnessing the intrinsic antioxidative and anti-inflammatory properties of the polysaccharides (Sun et al., 2025). Likewise, nanoparticles self-assembled from *L. barbarum* polysaccharide effectively preserved visual function in a mouse model of retinal ischemia-reperfusion by inhibiting retinal ganglion cell ferroptosis and attenuating neuroinflammation (Ni et al., 2024). This finding is consistent with the superior retinal neuroprotection observed after oral administration of submicron Lycium barbarum particles in rats (Wu I. H. et al., 2020). Moreover, in a mouse model of spinal cord injury, TSIIA/TMP/APS@Se NPs, a novel nanocarrier functionalized with *astragalus* polysaccharide (APS) and loaded with TSIIA and TMP, facilitated functional recovery by suppressing neuronal ferroptosis and promoting microglial polarization towards the beneficial M2 phenotype (Mai et al., 2025).

Simultaneously, recent studies have revealed the existence of natural microchannels connecting the skull bone marrow and the dura mater, representing a novel pathway for brain targeted drug delivery. In a rat model of permanent ischemic stroke, a microporous injection approach delivering neuroprotective agents via the skull bone marrow achieved higher cerebral drug concentrations at lower doses compared with conventional intravenous administration (Liu W. et al., 2025). This strategy highlights the potential of exploiting anatomical microchannels to enhance CNS drug delivery. In the future, integrating these innovative administration routes with advanced plant polysaccharide nanoparticles may further expand therapeutic options for CNS disorders, enabling more precise, efficient, and safe delivery of neuroprotective agents.

## 6 Clinical translation: current evidence and challenges

Despite encouraging preclinical findings, the clinical translation of plant polysaccharides remains at an early stage. A systematic search of major clinical trial registries, including ClinicalTrials.gov, WHO ICTRP, ChiCTR, and the EU Clinical Trials Register, indicates that no large-scale Phase III trials have yet been completed evaluating plant polysaccharide preparations as standalone therapeutic agents for CNS disorders. Notably, a prospective registered clinical trial investigating standardized *L. barbarum* polysaccharides for optic nerve protection exemplifies a feasible translational strategy, providing preliminary human evidence regarding both safety and potential efficacy, while simultaneously targeting an accessible component of the CNS with a well-documented history of traditional medicinal use. Nevertheless, the clinical translation of plant polysaccharides remains constrained by their structural complexity, batch-to-batch variability, and inherent multi-target pharmacological profiles, all of which impact bioavailability, pharmacokinetics, and the identification of mechanism-based biomarkers and clinically relevant endpoints. Their high molecular weight and hydrophilic nature limit gastrointestinal absorption and penetration across the blood–brain barrier, while current preclinical models provide only limited predictive value for human outcomes. Overcoming these challenges is critical to facilitate the development of reliable, effective, and translatable polysaccharide-based therapeutics.

## 7 Conclusion and perspectives

Neurological injuries and disorders impose profound socioeconomic and public health burdens worldwide, which highlights the urgent need to develop neuroprotective agents that are both effective and safe. Plant polysaccharides have garnered substantial scientific interest in recent years owing to their broad accessibility, favorable safety profiles, and intrinsic ability to modulate multiple pathological pathways in an integrated manner. In this review, we conducted a systematic analysis of 72 studies on plant polysaccharides to comprehensively evaluate their neuroprotective effects. Among these, 13 studies employed combined *in vivo* and *in vitro* experimental approaches, 40 studies focused exclusively on *in vivo* models, and 19 studies were confined to *in vitro* experiments. Across these diverse experimental systems, plant polysaccharides consistently demonstrated therapeutic efficacy, exemplified by the anti-inflammatory activity of *Astragalus* polysaccharides and the antioxidant and anti-apoptotic effects of *L. barbarum* polysaccharides. Taken together, these convergent findings provide mechanistic evidence supporting the potential clinical translation of plant polysaccharides for the treatment of CNS disorders.

## 8 Limitations of current research

Through a detailed examination of “Summary of Polysaccharide Sources, Extraction, Purity, and Structural Characterization” in Supplementary Table 2 and “Pharmacological Research Conditions” in Supplementary Table 3, it becomes evident that despite the expanding body of evidence supporting the neuroprotective potential of plant polysaccharides, several fundamental scientific and methodological limitations continue to constrain current progress and impede clinical translation.

First, a major limitation lies in the insufficient structural characterization of plant polysaccharides. Although the neuroprotective potential of these macromolecules is increasingly recognized, many studies still provide only fragmentary information on their fine structures. Critical parameters such as monosaccharide composition, linkage patterns, molecular weight distribution, branching architecture, and higher-order conformations are often incompletely defined. The absence of rigorous structural elucidation hinders the establishment of reliable structure–activity relationships and contributes to pronounced batch-to-batch variability caused by differences in plant origin, seasonal fluctuations, and extraction or purification procedures. Consequently, these inconsistencies significantly weaken cross-study reproducibility and complicate mechanistic interpretation.

Second, meaningful progress is limited by several weaknesses in current pharmacological study design. Many studies rely on single high-dose regimens without establishing dose–response profiles or defining therapeutic windows, which restricts accurate evaluation of potency and safety. Mechanistic claims are frequently based on parallel changes in signaling molecules rather than on targeted validation through genetic knockdown, receptor antagonism, or pathway-specific inhibition. Moreover, an overreliance on reductionist *in vitro* systems or simplified animal models restricts the translational relevance of findings to human neuropathology. This limitation is particularly pronounced in studies of polysaccharide-mediated neuroprotection, where *in vitro* models circumvent critical processes such as blood–brain barrier penetration and fail to capture the full complexity and dynamic characteristics of *in vivo* responses.

Third, substantial uncertainty persists regarding the bioavailability and CNS exposure of polysaccharides. Because these molecules are typically hydrophilic and possess large molecular sizes, they often exhibit minimal gastrointestinal absorption and extremely limited penetration across the blood–brain barrier. Direct evidence demonstrating that intact polysaccharides or biologically active metabolites reach the CNS at meaningful concentrations remains scarce. This knowledge gap makes it difficult to determine whether the reported neuroprotective effects arise from direct central actions or are predominantly mediated through peripheral routes, including modulation of gut microbiota, immune regulation, or systemic metabolic effects. Human pharmacokinetic and biodistribution data remain critically inadequate.

Finally, clinical translation remains limited owing to the scarcity of high-quality human evidence. Although extensive preclinical work consistently demonstrates antioxidant, anti-inflammatory, and anti-apoptotic effects, no large-scale Phase III trials have been completed to evaluate polysaccharides as independent therapeutic agents for neurological disorders. Early-phase clinical studies, such as those investigating standardized Lycium barbarum polysaccharides for optic nerve protection, provide preliminary proof-of-concept but remain insufficient to establish clinical efficacy. In addition, the intrinsically multi-target nature of polysaccharides complicates the identification of mechanistic biomarkers and the selection of clinically meaningful endpoints, which further increases the difficulty of designing robust clinical trials.

## 9 Future perspectives

Advancing plant polysaccharides toward clinically viable neuroprotective agents will require coordinated progress in structural standardization, mechanistic elucidation, translational modeling, and clinical evaluation. Several areas of future research are expected to play decisive roles in enhancing reproducibility, clarifying mechanisms, and improving clinical readiness.

First, addressing the limitation of insufficient structural characterization requires enhanced standardization and analytical rigor. Future investigations should adopt consistent procedures for plant sourcing, extraction, and purification, combined with bioactivity-guided fractionation to isolate structurally well-defined polysaccharide preparations suitable for mechanistic analysis. High-resolution analytical techniques, including HPSEC–MALLS, GC–MS, NMR, FT–IR, and emerging mass spectrometry-based conformational methods, are essential for establishing robust and reproducible structure–function relationships.

Second, overcoming weaknesses in pharmacological study design requires the implementation of rigorous dose-response frameworks and improved experimental validation. Future studies should examine multiple dosage levels, define therapeutic windows, and employ genetic or pharmacological interventions to confirm mechanistic pathways. Simultaneously, integrating *in vivo* and *in vitro* models that more closely recapitulate human neuropathology will enhance translational relevance.

Third, the unresolved limitation of limited bioavailability and central nervous system exposure underscores the need for strategies to enhance delivery and clarify pharmacokinetic profiles. Research should explore advanced delivery approaches, including ligand-modified nanoparticles, intranasal administration, and stimuli-responsive carriers, to overcome blood–brain barrier constraints. Concurrently, the role of gut microbiota in metabolizing polysaccharides and generating bioactive metabolites should be systematically investigated.

Finally, enhancing translational research and clinical pipelines is essential for bridging the gap between preclinical findings and human applications. Advanced experimental models, including humanized systems, organoids, microfluidic blood–brain barrier chips, and multicellular co-culture platforms, provide physiologically relevant contexts for evaluating the behavior of large hydrophilic macromolecules and predicting human-relevant pharmacokinetics. At the same time, early-phase clinical trials are needed to systematically assess safety, pharmacokinetics, and mechanistic biomarkers. Successful translation will depend on coordinated interdisciplinary efforts among natural product chemists, neurobiologists, clinicians, materials scientists, and systems pharmacologists.
