# Supplementary material for: Plant polysaccharides in neuroprotection: mechanistic insights across central nervous system disorders
Source: Front Pharmacol. 2026 Feb 11;16:1727705. doi: 10.3389/fphar.2025.1727705 (PMC12932451; doi:10.3389/fphar.2025.1727705)
Supplement: Supplementary file 1 [file Table1.pdf]

Supplementary Table 1 Molecular weight, monosaccharide composition, and structural characteristics of plant polysaccharides

Fraction	Source	Mw(kDa)	Monosaccharide composition	Structural features	Mechanism	Biological activities	ref
RGP	*Rehmannia glutinosa*	145.821 kDa	Fuc, Rha, Ara, Gal, Glc, Xyl, Man (Molar ratio:1.05:18.98:28.12:30.54:12.60:3.26:5.49)	-	RGP significantly reduced the neuroprotective effects on photo-induced oxidative stress and autophagy in HT-22 cells by inhibiting the AKT/mTOR pathway and increasing autophagy-related protein levels.	mitigate excessive autophagy	Yang et al., (2024)
NPGE	*Gastrodia elata*	~12 kDa	-	-	Downregulating IL-1β, IL-6, TNF-α, NLRP3, and HMGB1 levels to suppress ferroptosis-mediated neuroinflammation, while upregulating NRF2 and HO-1 expression to facilitate NRF2 nuclear translocation.	suppression of ferroptosis	Zhang et al., (2024)
LBP	*Lycium barbarum*	22.355 kDa	Rha, Ara, Gal, Glc, Man, Rib, Glc-UA (Molar ratio: 2.04:9.50:19.43:1.37:1.27:64.76:1.63)	-	Activating the Nrf2/HO-1 signaling pathway to rescue light-induced neurotoxicity in both mouse and HT-22 cells	inhibition of oxidative stress	Yang et al., (2023)
MCPs	*Momordica charantia*	-	GalA (93.7%)，Rha, Xy, Man, Glc, Gal (Molar ratio: 0.06% : 0.18% : 0.03% : 0.8% : 0.28%)	-	promote neurogenesis after cerebral ischemia-reperfusion injury through the SIRT1-mediated deacetylation and nuclear translocation of β-catenin, which shifts neural stem cell differentiation from gliogenesis to neurogenesis.	regulate NSC fate determination	Hu et al., (2020)

Fraction	Source	Mw(kDa)	Monosaccharide composition	Structural features	Mechanism	Biological activities	ref
SCP2-1	*Schisandra chinensis (Turcz.)* Baill	-	Glc (87%),Gal(13%)	-	Enhancing LRP-1 expression inhibits NF-κB and MAPK (particularly JNK) signaling pathways, thereby reversing the M1/M2 polarization of microglia.	blockade of neuroinflammation	Xu et al., (2020)
LBP	*Lycium barbarum*	-	Rha, Ara, Xyl, Gal, Man, GalA	-	Inhibits the NR2B signaling pathway (NR2B, nNOS, BAD, CytC, cleaved CASP3), thereby reducing ROS, calcium influx, and mitochondrial permeability	inhibition of apoptosis	Shi et al., (2017)
APS	*Astragalus* membranaceus	-	Man, Glc, Xyl, Ara, GlcA, Rha	-	facilitate NSC lineage specification by suppressing stemness maintenance and astrocytic differentiation	regulate NSC fate determination	Ye et al., (2021)
DOP	*Dendrobium officinale*	746.52 kDa	Man, Glc，Molar ratio 2.55:1.00	-	By significantly upregulating Akkermansia and downregulating Clostridium to reshape the gut microbiota, this approach reduces intestinal permeability, inhibits LPS-stimulated NF-κB activation and inflammatory cell infiltration, and decreases Aβ deposition.	regulation of gut microbiota	Sun et al., (2022)
DOP	*Dendrobium officinale*	-	Glc,Man	It contains a pyranose ring structure and features β-glycosidic bonds (β-glucoside bonds).	The study demonstrated that increasing Lactobacillus and decreasing Pseudomonas, combined with upregulation of tight junction proteins, enhanced intestinal barrier function and reversed Aβ25-35-induced disturbances in 19 metabolites. These effects	regulation of gut microbiota	Fu et al., (2025)

Fraction	Source	Mw(kDa)	Monosaccharide composition	Structural features	Mechanism	Biological activities	ref
					involved linoleic acid, arachidonic acid, and alpha-linolenic acid metabolism, as well as the tricarboxylic acid cycle, thereby improving energy metabolism and cognitive function.		
PSP-1	*Polygonatum sibiricum*	18.796 kDa	-	-	This approach reduces Helicobacter pylori and promotes the growth of Akmanella mucinolytica, thereby decreasing intestinal permeability and Aβ deposition in the colon. Simultaneously, it enhances microglia's phagocytic activity against plaques, reducing Aβ accumulation and synaptic loss in the brain, ultimately improving memory deficits.	regulation of gut microbiota	Luo et al., (2022)
SBP	*Hippophae rhamnoides* L.	58.78 kDa	Ara:Glc:Gal:Rha:Xyl:Fuc:GalA (5.34:0.65:0.61:0.60:0.26:0.06:2.48)	3,4-β-L-Rhap and 1,4-α-D-GalAp	reversed the decline of Ileibacterium while suppressing the overgrowth of genera such as Lactobacillus, Dubosiella, Olsenella, Helicobacter, and Ruminiclostridium 9, enhanced tight junction proteins, attenuated neuroinflammation by inhibiting NF-κB signaling, and restored synaptic plasticity via the CREB/BDNF/TrkB pathway.	regulation of gut microbiota	Lan et al., (2023)
FEP	*Fagopyrum esculentum*	160 kDa	Rha:Glc:Gal (1:67.4:1.9)	-	increase SCFAs (acetic, propionic, butyric acids) and enrich SCFA-producing bacteria (Eubacterium xylanophilum group, Lachnospiraceae NK4A136 group, Lactobacillus),	regulation of gut microbiota	Liu et al., (2024)

Fraction	Source	Mw(kDa)	Monosaccharide composition	Structural features	Mechanism	Biological activities	ref
					thereby reducing neuroinflammation, decreasing AD-related pathology, and improving cognitive deficits.		
GEP	*Gastrodia elata*	42.58 × 10^4 Da	Glc, Gal, GalA	The structure consists of α-glucopyranose units, featuring a glucan backbone comprising α-1,4-, α-1,6-, α-1,3,4-, and β-1,6-linked residues.	counteract gut dysbiosis by modulating key genera including Akkermansia, Lactobacillus, Bacteroides, Prevotella, and Faecalibacterium, elevate the colonic SCFA content and mitigate mitochondrial apoptosis-driven dopaminergic neuron loss; strengthens intestinal barrier integrity, thereby suppressing TLR4/NF-κB pathway activation and alleviate neuroinflammation	regulation of gut microbiota	Gan et al., (2024)
APS	*Astragalus* membrana ceus	7827-12856 Da	Glc (92.5%), Gal (2.9%), Ara (1.5%), Xyl (0.9%), Man (0.8%), GalA (0.8%), Rha (0.6%)	-	modulate gut microbiota composition, such as Romboutsia, Rikenella, Dubosiella, and Odoribacter, restores neuronal homeostasis and inhibits neuroinflammation through taurine and hypotaurine metabolism, phenylalanine metabolism, pantothenate and coenzyme A biosynthesis, citrate cycle, and propanoate metabolism	regulation of gut microbiota	Liu et al., (2025)
CYP	*Corydalis yanhusuo*	-	-	-	Polysaccharides modulate the gut microbiota to increase SCFAs, which subsequently elevate key neurotransmitters including dopamine, norepinephrine, and 5-HT (by influencing TPH-1/2 enzymes and neurotransmitter reuptake), as well as	regulation of gut microbiota	Fang et al., (2023)

Fraction	Source	Mw(kDa)	Monosaccharide composition	Structural features	Mechanism	Biological activities	ref
					boost BDNF levels, thereby ameliorating neuronal damage and depressive states.		
SCP	*Schisandra chinensis (Turcz.) Baill*	-	Ara, Glu, Gal, Man, Rib, Xyl, GlcA, Rha, Fuc	-	alleviates Alzheimer's disease pathology by regulating gut microbial metabolism and energy metabolism, which in turn reduces Aβ deposition and p-tau phosphorylation, mitigates oxidative stress, and normalizes the imbalance of key neurotransmitters such as γ-aminobutyric acid, dopamine, and glutamate	regulation of gut microbiota	Liu et al., (2019)
AMP	*Aronia melanocarpa*	-	Fuc (0.14%), Rha (0.73%), Ara (7.14%), Gal (10.61%), Glc (76.16%), Xyl (2.31%), Man (1.25%), Gal-UA (1.43%), Glc-UA (0.16%), Man-UA (0.07%)	-	Block IκB-α phosphorylation, suppress NF-κB overexpression, and alleviate D-Gal-induced inflammation	blockade of neuroinflammation	Zhao et al., (2021)
SNP2-A	*Scrophularia ningpoensis*	24.0 kDa	Man, Rha, Glu A, Gal A, Glu, Gal, Xyl, Ara	-	Enhance ERK, JNK, and p38 protein expression (MAPK pathway)	blockade of neuroinflammation	Ma et al., (2019)
SCP	*Schisandra Chinensis Fructus*	-	Man, Rha, GlcA, Glc, Gal, Ara	-	Reduce Aβ deposition, activate NF-κB/MAPK pathway, control p-38, JNK, and ERK phosphorylation, and decrease inflammatory factor release	blockade of neuroinflammation	Xu et al., (2019)

Fraction	Source	Mw(kDa)	Monosaccharide composition	Structural features	Mechanism	Biological activities	ref
EbPS-A1	*Epimedium brevicornum*	-	GalA (56.7%), Gal (19.4%), Rha (16.1%), Ara (5.9%), GlcA (2.0%)	-	Eliminate free radicals, reduce ROS, enhance SOD activity, and decrease MDA	inhibition of oxidative stress	Xiang et al., (2017)
PSPE	*Polygonatum sibiricum*	252.5 kDa (44.97%), 1.277 kDa (55.03%)	Gal (67.28%), Man (14.38%), GalA (9.11%), Ara (4.76%), Glc (4.46%)	-	Enhance ROS levels, boost SOD and CAT activity, and increase antioxidant gene expression	inhibition of oxidative stress	Zhang et al., (2022)
CPP	*Cyclocarya paliums*	84,061.23 Da to 320.36 Da	-	-	Reduce ROS and peroxidation products (MDA, NEFA, GSSG) while enhancing antioxidant enzymes (SOD, CAT, GSH-Px) and GSH levels	inhibition of oxidative stress	Lin et al., (2020)
HRPI	*Hippophae rhamnoides L.*	19,138 Da	Man:Rha:Glc:Gal:Ara = 14:35:30.00:24.91:14.77:15.97	-	Inhibit Keap1 to enhance the expression of Nrf2 and antioxidant enzymes SOD and GSH-Px	inhibition of oxidative stress	Zhao et al., (2023)
PSP	*Polygonatum cyrtonema Hua*	6–14 kDa	-	-	Enhance Nrf2 and HO-1 expression and SOD activity in the hippocampus, while reducing MDA levels	inhibition of oxidative stress	Xie et al., (2024)
ASP	*Angelica sinensis*	Molecular weight not specified (HPLC indicated a homogeneous	Xyl, Gal, Glc, Ara, Fru, GlcA	-	Activate the PI3K/AKT pathway, upregulate Bcl-2, and inhibit cleaved-CASP3 and Bax	inhibition of apoptosis	Xu et al., (2021)

Fraction	Source	Mw(kDa)	Monosaccharide composition	Structural features	Mechanism	Biological activities	ref
		polysaccharide, dialysis cutoff: 3.5 kDa)					
BRP	*Angelica sinensis*	-	Gal, Rha, GalA, Glc	-	Enhance PI3K and Akt phosphorylation, upregulate HIF-1α, downregulate CASP3 and Bax, and upregulate Bcl-2	inhibition of apoptosis	Zou et al., (2022)
DP	*Dendrobium officinale*	312 kDa	Man 16.68%, Rib 0.24%, Rha 0.60%, GlcA 0.62%, GalA 1.29%, Glu 56.24%, Gal 3.67%, Xyl 0.64%, Ara 0.77%, Fuc0.07%	-	Enhance AMPK phosphorylation and mitochondrial function, promote DNA demethylation, inhibit Bax, Bak, and cleaved CASP3, and increase Bcl-2	inhibition of apoptosis	Chen et al., (2023)
CPPs	*Codonopsis pilosula*	-	Rha, Ara, Gal, Glc	-	Promote endoplasmic reticulum morphological recovery, reduce GRP78, ATF4, and CHOP expression, and lower the p-PERK/PERK ratio	inhibition of apoptosis	Cai et al., (2025)
PCP	*Polygonatum cyrtonema*	8.5 kDa	-	→6)-β-D-Fruf-(2→, →1,6)-β-D-Fruf-(2→, →1)-β-D-Fruf-(2→, β-D-Fruf-(2→, →6)-α-D-Galp-(1→	Reverse the upregulation of endoplasmic reticulum stress-related proteins (ATF6, BIP, CHOP, etc.), reduce Bax and cleaved CASP3, and enhance Bcl-2	inhibition of apoptosis	Li et al., (2025)
APS	*Astragalus*	1.7×10^6 Da	Rha, Glc, Gal	-	APS enhances cellular viability and autophagy levels by improving autophagosome formation and promoting the conversion of LC3-I to LC3-II. It also downregulates pAKT and mTOR expression while	regulation of autophagy	Tan et al., (2020)

Fraction	Source	Mw(kDa)	Monosaccharide composition	Structural features	Mechanism	Biological activities	ref
					upregulating PTEN expression.		
SMP1	*Salvia miltiorrhiza Bunge*	6087 Da	Glc, Gal, Fru（Molar ratio:1:1.67:1.12）	-	By activating the Nrf2/HO-1 pathway in PC12 cells, it protects against ferroptosis and lipid peroxidation induced by OGD/R.	suppression of ferroptosis	Meng et al., (2022)
CPPs	*Codonopsis pilosula*	-	Man, Glc, Ara (1.76%, 97.38%, 0.76%)	-	elevated the intracellular levels of NAD^+ and the NAD^+/NADH ratio, as well as upregulated the expression of NAD^+-dependent deacetylases SIRT1 and SIRT3	enhancing energy metabolism and supporting neuronal growth and survival	Hu et al., (2021)
RP01-1	*Polygala tenuifolia Willd.*	79.1 kDa	Rha (7.3%), GalA (8.2%), Ara (65.6%), Gal (18.9%)	The main chain is composed of alternating 1,2,4-linked α-Rhap and 1,4-linked α-GalpA residues.	Enhance BDNF expression and promote the phosphorylation of AKT, ERK, and CREB to induce neurotrophic growth in PC12 cells	enhancing energy metabolism and supporting neuronal growth and survival	Zeng et al., (2020)
ATP50-3	*Acorus tatarinowii*	87.3 kDa	Ara, Rha, Xyl, GlcA, Gal, GalA, Glc, Man	The main chain is primarily composed of β-D-galactopyranose (β-D-Galp) residues with a β-configuration. The glycosidic linkage types include:<br><br>Linear linkages: 1→3 and 1→6 glycosidic bonds (e.g., →3)-β-D-Galp-(1→ and →6)-β-D-Galp-(1→).<br><br>Branched linkages: Branch	Inhibiting neuroinflammation through TLR4/MyD88/NF-κB and PI3K/Akt signaling pathways	blockade of neuroinflammation	Zhong et al., (2020)

Fraction	Source	Mw(kDa)	Monosaccharide composition	Structural features	Mechanism	Biological activities	ref
				points exist along the main chain, involving multi-site linkages such as 1→3,6 and 1→3,4,6 (e.g., →3,6)-β-D-Galp-(1→ and →3,4,6)-β-D-Galp-(1→).			
ARP-1	*Asteris Radix et Rhizoma (Aster tataricus L. f.)*	214 kDa	Fuc:Ara:Gal:Glc:Man = 0.40:14.25:10.22:1.06:0.41（Molar ratio）	→3,6)-β-D-Galp-(1→ and →6)-β-D-Galp-(1→ residues	Eliminate ABTS, hydroxyl, and DPPH free radicals, reduce ROS and MDA, and enhance SOD activity	inhibition of oxidative stress	Zhu et al., (2022)
PGP1	*Platycodon grandiflorum*	5.9 kDa	Glc:Gal:-Man = 7.1:1.1:0.6（Molar ratio）	-	Enhance SOD activity to reduce intracellular ROS, MDA, and LDH	inhibition of oxidative stress	Sheng et al., (2017)
LICP009-3F-1a	*Lycium barbarum L.*	10,780 Da	arabinose, galactose, glucose, xylose, mannose in a ratio of 36:4:239.1:80.8:24.1:9.7	→4)-β-D-Glcp-(1→6)-β-D-Galp-(1→	Activate SOD, CAT, and GPx genes to boost antioxidant enzyme activity and reduce ROS	inhibition of oxidative stress	Li et al., (2025)
CCP	*Coptis chinensis*	3.96 kDa	Glc	-	Inhibits JNK phosphorylation, enhances mitochondrial function, and regulates Bax/Bcl-2 and CASP3	inhibition of apoptosis	Li et al., (2019)
PPV-6	*Basella alba*	Mw 134.14 ± 0.02 kDa	Glc (40.20 ± 0.27%), Gal (30.76 ± 0.41%), Ara (24.40 ± 0.09%), Rha (3.54 ± 0.04%), Gal-UA (0.73 ± 0.18%), Glc-UA (0.38 ± 0.01%)	-	Downregulating SHH expression inhibits neuronal CCR and subsequent apoptosis	inhibition of apoptosis	Hou et al., (2024)

Fraction	Source	Mw(kDa)	Monosaccharide composition	Structural features	Mechanism	Biological activities	ref
CYP	*Corydalis yanhusuo*	75.5 kDa	Glc	(1→4,6)-linked-Glc and (1→)-linked-Glc（ratio:1:1）	Reduce the Bax/Bcl2 ratio and decrease cleaved CASP8, CASP9, and CASP3	inhibition of apoptosis	He et al., (2020)

Supplementary Table 2 Summary of Polysaccharide Sources, Extraction, Purity, and Structural Characterization.

Polysaccharide Name (Abbreviation)	Source	Extraction Method	Purity	Structural Elucidation Status	Reference
Potentilla anserina L. Polysaccharide (PAP)	Potentilla anserina L. (from Yushu City, Qinghai Province, China)	Water extraction and alcohol precipitation (ethanol-insoluble fraction)	Not specified	No. (Monosaccharide composition, molecular weight, linkage types not provided)	Cheng et al., 2021
Rehmannia glutinosa Polysaccharide (RGP)	Rehmannia glutinosa (Scrophulariaceae)	Water extraction, alcohol precipitation, deproteinized using Sevag method (chloroform/n-butanol, 4:1, v/v), lyophilized	Purity value not specified; purification steps confirmed	Yes. (Monosaccharide composition, molecular weight, FT-IR, SEM provided)	Yang et al., 2024
Lycium	Lycium barbarum (Goji	Provided by Shanghai Institute of Organic Chemistry, Chinese	> 60%	No. (Monosaccharide composition, molecular	Li et al., 2023

Polysaccharide Name (Abbreviation)	Source	Extraction Method	Purity	Structural Elucidation Status	Reference
barbarum Polysaccharide (LBP)	berry)	Academy of Sciences (method not detailed)		weight, linkage types not provided)	
Gastrodia elata Neutral Polysaccharide (NPGE)	Gastrodia elata	Provided by Department of Pharmacy, Renmin Hospital of Wuhan University (method not detailed)	Purity value not specified	No. (Detailed structural information like monosaccharide composition, backbone linkages, branching not provided; only molecular weight ~12 kDa mentioned)	Zhang et al., 2024
Glycyrrhiza Polysaccharide (GP)	Glycyrrhiza (Licorice)	Water extraction, defatted with ethyl acetate, purified using AB-8 macroporous resin, lyophilized	95% (determined by phenol-sulfuric acid method)	No. (Monosaccharide composition, molecular weight, backbone/branching structure not provided)	Du et al., 2024
Lycium barbarum Polysaccharide (LBP)	Lycium barbarum (Solanaceae)	Water extraction, alcohol precipitation, deproteinized using Sevag method (chloroform/n-butanol, 4:1, v/v), lyophilized	Purity value not specified; purification steps confirmed	Yes. (Monosaccharide composition, molecular weight, FT-IR, SEM provided)	Yang et al., 2023

Polysaccharide Name<br><br>(Abbreviation)	Source	Extraction Method	Purity	Structural Elucidation Status	Reference
Momordica charantia Polysaccharides (MCPs)	Momordica charantia (Bitter melon)	Water extraction, alcohol precipitation, removal of proteins and starch	≥ 99% (characterized by HPLC-MS/MS)	Yes. (Detailed monosaccharide composition and molar ratios provided; primarily galacturonic acid, 93.7%)	Hu et al., 2020
Astragalus Polysaccharide (APS)	Astragalus (Milkvetch)	Commercially purchased (Beijing Solarbio Science & Technology Co., Ltd.)	Not specified	No. (Structural information not provided)	Jia et al., 2022
SCP2-1	Schisandra chinensis (Turcz.) Baill	Water extraction, alcohol precipitation, purified sequentially by DEAE-Sepharose Fast Flow and Sepharose CL-6B chromatography	Purity value not specified; purification steps confirmed; protein content 0.75%	Partial. (Monosaccharide composition and ratios provided; molecular weight, detailed linkage information not provided)	Xu et al., 2020
Potentilla anserina L. Polysaccharide (PAP)	Potentilla anserina L. (from Yushu City, Qinghai Province, China)	Water extraction and alcohol precipitation (ethanol-insoluble fraction)	Purity value not specified; purification steps confirmed	No. (Monosaccharide composition, molecular weight, linkage types not provided)	Cheng et al., 2022
Lepidium	Lepidium meyenii Walp.	Water extraction, alcohol precipitation, deproteinized using	Polysaccharide content 71.54%	No. (Monosaccharide composition, backbone	Zhou et al.,

Polysaccharide Name (Abbreviation)	Source	Extraction Method	Purity	Structural Elucidation Status	Reference
meyenii Polysaccharide (MP)	(Maca)	Sevag method, vacuum dried	(phenol-sulfuric acid method)	linkages, branching structure not provided)	2022
Lycium barbarum Polysaccharide (LBP)	Lycium barbarum (Goji berry)	Water extraction (specific steps not detailed)	Not specified	No. (LBP mentioned as a mixture containing Rha, Ara, Xyl, Gal, Man, GalA, but detailed structural information not provided)	Shi et al., 2017
Lonicera japonica Polysaccharide (LJP)	Lonicera japonica flowers (Henan Province, China)	Defatted with ethanol reflux, hot water extraction, deproteinized with trichloroacetic acid, alcohol precipitation, vacuum dried	Yield 5.1% (w/w); purity not specified	No. (Monosaccharide composition, sequence, linkage types not provided)	Wang et al., 2021
Lycium barbarum Polysaccharide (LBP)	Lycium barbarum	Not specified	Not specified	No. (No structural characterization data provided)	Wang et al., 2018
Morinda officinalis Polysaccharide (MOP)	Morinda officinalis	Not specified (Purchased from Vicki Biotechnology Co., Ltd.)	Not specified	No. (Structural details not provided)	Dong et al., 2025

Polysaccharide Name (Abbreviation)	Source	Extraction Method	Purity	Structural Elucidation Status	Reference
Dendrobium nobile Polysaccharides (DNP)	Dendrobium nobile (Orchidaceae)	Water extraction, alcohol precipitation, purified by DEAE Sepharose Fast Flow column chromatography	98.1% (determined by phenol-sulfuric acid method)	No. (Only purity reported; no structural details)	Ming et al., 2023
Astragalus Polysaccharides (APS)	Astragalus membranaceus (Milkvetch)	Not specified (Purchased from Yuanye Biotech, Shanghai, China)	≥98%	Partial. (Monosaccharide composition provided; lacks details on backbone configuration, glycosidic linkages, molecular weight)	Ye et al., 2021
Astragalus Polysaccharides (APS)	Astragalus membranaceus	APS purchased from Yuanye Bio-Technology (extraction method not described)	≥98% UV	No. (Monosaccharide composition, linkage types, branching structure not provided)	Zhao et al., 2024
Dendrobium officinale Polysaccharide (DOP)	Dendrobium officinale (Orchidaceae)	Water extraction, alcohol precipitation, deproteinized using Sevag method (chloroform:n-butanol=4:1), dialysis, lyophilized	80.57% ± 0.49% (determined by phenol-sulfuric acid method)	Yes. (Monosaccharide composition, molecular weight and distribution, UV spectrum confirming absence of protein/nucleic acid provided)	Sun et al., 2022

Polysaccharide Name (Abbreviation)	Source	Extraction Method	Purity	Structural Elucidation Status	Reference
Dendrobium officinale Polysaccharide (DOP)	Stems of Dendrobium officinale (Orchidaceae)	Water extraction, alcohol precipitation (4 vol ethanol), defatted with petroleum ether, deproteinized using Sevag method, dialysis (MWCO 2000 Da), lyophilized	Specific purity value stated	Yes. (Monosaccharide composition, IR spectrum, molecular weight distribution, SEM provided; pyranose ring and β-glycosidic linkage confirmed by NMR, citing previous study [24] and Fig. S1)	Fu et al., 2025
PSP-1	Polygonatum sibiricum	Purified by DEAE-52 cellulose column chromatography and Sephacryl-200 gel filtration	PDI=1.15, indicating high homogeneity	No. (Monosaccharide composition and chain structure not resolved)	Luo et al., 2022
Sea Buckthorn Polysaccharide (SBP)	Berries of Hippophae rhamnoides L.	Decoction in water followed by alcohol precipitation	Carbohydrates 65.68%, Protein 1.63%, Uronic acid 27.80%	Yes. (Monosaccharide composition, molecular weight, information on backbone and branching provided)	Lan et al., 2023
Fagopyrum esculentum Polysaccharide (FEP)	Common Buckwheat (Fagopyrum esculentum)	Hot water extraction, deproteinized with trichloroacetic acid, alcohol precipitation,	Purity value not specified; purification steps	Yes. (Comprehensive characterization: Monosaccharide composition, molecular	Liu et al., 2024

Polysaccharide Name (Abbreviation)	Source	Extraction Method	Purity	Structural Elucidation Status	Reference
		dialysis, lyophilized	confirmed	weight, FT-IR, XRD, DSC, TGA, SEM provided)	
Gastrodia elata Polysaccharide (GEP)	Gastrodia elata	Water extraction, alcohol precipitation, deproteinized using Sevag method, lyophilized	Total sugar content 89.26%, Protein 1.75%	Yes. (Monosaccharide composition, molecular weight, FT-IR, glycosidic linkage types provided)	Gan et al., 2024
Astragalus membranaceus Bunge Polysaccharides (APS)	Astragalus membranaceus Bunge	Commercially purchased (Yuanye Biotechnology Co., Ltd.)	Purity >98%	Yes. (Monosaccharide composition, molecular weight, FT-IR spectrum provided)	Liu et al., 2025
Corydalis yanhusuo Polysaccharide (CYP)	Corydalis yanhusuo W.T. Wang (Papaveraceae)	Commercially purchased (Sichuan Welkedj Biotechnology Co., Ltd., Product No.: WKQ-0008441); Supplementary Material S1 describes extraction via water extraction and alcohol precipitation	Purity value not specified; described as crude polysaccharide	No. (Lack of structural characterization explicitly stated; molecular weight, monosaccharide composition require further study)	Fang et al., 2023
Schisandra	Schisandra	Water extraction, ethanol	Total sugar 58.65%, Protein	Partial. (Monosaccharide composition provided; lacks	Liu et al., 2019

Polysaccharide Name (Abbreviation)	Source	Extraction Method	Purity	Structural Elucidation Status	Reference
chinensis Polysaccharide (SCP)	chinensis (Turcz.) Baill	precipitation (70% ethanol)	2.65%; Purities of fractions ranged 26.94%-79.60%	linkage types, branching, or detailed structure)	
Lycium barbarum Polysaccharide (LBP)	Lycium barbarum	Not specified	Not specified	No. (No structural features reported)	Song et al., 2022
Aronia melanocarpa Polysaccharide (AMP)	Fruit of Aronia melanocarpa	Water extraction, alcohol precipitation, preliminary purification with D101 macroporous resin, purified by DEAE-52 cellulose column chromatography	Purity value not specified; purification steps confirmed	Partial. (Precise monosaccharide composition with molar percentages and FT-IR spectrum provided)	Zhao et al., 2021
Potentilla anserina L. Polysaccharide (PAP)	Root of Potentilla anserina L.	Water extraction, alcohol precipitation, defatted with petroleum ether, deproteinized by enzymatic and Sevag methods, lyophilized	Total sugar content 86.92% (phenol-sulfuric acid method)	No. (Only total sugar content and extraction method provided; monosaccharide composition, molecular weight, linkage types not detailed)	Shi et al., 2020
Scrophularia ningpoensis Polysaccharide	Root of Scrophularia	Water extraction, alcohol precipitation, Sevag	Neutral sugar content 93.5%,	Yes. (Monosaccharide composition and molar	Ma et al., 2019

**Polysaccharide Name (Abbreviation)**	**Source**	**Extraction Method**	**Purity**	**Structural Elucidation Status**	**Reference**
(SNP2-A)	ningpoensis	deproteinization, dialysis (50 kDa MWCO), purified by DEAE-52 cellulose and Sephadex G-100 gel filtration chromatography	Protein 2.4%, Uronic acid 1.6%	ratios, molecular weight, FT-IR, UV spectrum, SEM provided)	
Lycium barbarum Polysaccharide (LBP)	Lycium barbarum (Goji berry)	Provided by Ningxia Academy of Agriculture and Forestry Sciences (method not detailed)	Not specified	No. (Monosaccharide composition, molecular weight, linkage types not provided)	Zhao et al., 2017
Schisandra Chinensis Fructus Polysaccharide (SCP)	Schisandra Chinensis Fructus	Water extraction, alcohol precipitation, deproteinized using Sevag method, dialysis	Purity value not specified; purification steps confirmed	Partial. (Monosaccharide composition provided; lacks molecular weight and linkage details)	Xu et al., 2019
Epimedium brevicornum Polysaccharide (EbPS-A1)	Epimedium brevicornum	Hot water extraction, ethanol precipitation, purified by ion-exchange chromatography	Not specified	Partial. (Monosaccharide composition provided; lacks molecular weight and linkage details)	Xiang et al., 2017
Polygonatum sibiricum Polysaccharide	Polygonatum sibiricum	Water extraction, alcohol precipitation, deproteinized using	Total sugar 75.05%, Uronic acid 16.68%,	Yes. (Monosaccharide composition, molecular weight, FT-IR spectrum	Zhang et al., 2022

Polysaccharide Name (Abbreviation)	Source	Extraction Method	Purity	Structural Elucidation Status	Reference
Extract (PSPE)		triple-phase method, dialysis	virtually no protein	provided)	
Cyclocarya paliurus Polysaccharide (CPP)	Leaves of Cyclocarya paliurus (Batal.) Iljinskaja	Water extraction, filtration, concentration, lyophilization	Not specified	No. (Monosaccharide composition, linkage types not provided)	Lin et al., 2020
Astragalus Polysaccharide (APS)	Astragalus (Milkvetch)	APS nanoparticles prepared by PEG-PLA double emulsion method	99% purity (APS raw material)	No. (Polysaccharide structure information not provided)	Sun et al., 2020
Momordica charantia Polysaccharide (MCP)	Momordica charantia (Bitter melon)	Not specified	Not specified	No. (Structural information not provided)	Yue et al., 2023
Hippophae rhamnoides L. Polysaccharide I (HRPI)	Hippophae rhamnoides L. (Sea Buckthorn)	Water extraction, alcohol precipitation, deproteinization, decolorization, membrane separation	Carbohydrate content 68.59%, Protein 0.2%	Yes. (Monosaccharide composition, molecular weight, FT-IR, XRD, SEM provided)	Zhao et al., 2023
Polysaccharides from Polygonatum	Polygonatum cyrtonema Hua	Water extraction, alcohol precipitation (defatted with petroleum ether, water reflux,	Purity value not specified	Partial. (FT-IR provided; UPLC qualitative analysis indicated presence of six	Xie et al., 2024

Polysaccharide Name (Abbreviation)	Source	Extraction Method	Purity	Structural Elucidation Status	Reference
cyrtonema Hua (PSP)		deproteinized by Sevag method, dialysis)		monosaccharides/uronic acids)	
Angelica sinensis Polysaccharide (ASP)	Angelica sinensis	Water extraction, alcohol precipitation (water reflux, deproteinization by repeated freeze-thawing, ultrafiltration, dialysis, gel chromatography)	Homogeneous polysaccharide (single peak by HPLC)	Partial. (Monosaccharide composition and HPLC profile provided)	Xu et al., 2021
Brassica rapa Polysaccharide (BRP)	Brassica rapa L.	Water extraction, alcohol precipitation (defatted with 80% ethanol, water extraction, ethanol precipitation, deproteinized with chloroform-n-butanol, dialysis, lyophilized)	Purity value not specified	Partial. (Monosaccharide composition provided)	Zou et al., 2022
Angelica sinensis Polysaccharides (ASP)	Angelica sinensis	Extraction method not detailed	Not specified	No. (Monosaccharide composition, molecular weight, linkage types not provided)	Du et al., 2020
Dendrobium	Dendrobium officinale	Water extraction, alcohol precipitation, deproteinized using	Purity value not specified;	Yes. (Monosaccharide composition and molecular	Chen et al.,

Polysaccharide Name (Abbreviation)	Source	Extraction Method	Purity	Structural Elucidation Status	Reference
officinale Polysaccharide (DP)		Sevag method, dialysis, lyophilized	purification steps confirmed	weight provided)	2023
Codonopsis pilosula Polysaccharides (CPPs)	Codonopsis pilosula	CPPs purchased from Shanghai Shifeng Biotechnology Co., Ltd. (extraction method not detailed)	UV concentration 98%; polysaccharide purity not clearly defined	No. (Detailed structural information like monosaccharide molar ratios, molecular weight, linkage types missing)	Cai et al., 2025
Polygonatum cyrtonema Polysaccharide (PCP)	Polygonatum cyrtonema	Water extraction, alcohol precipitation, Sevag deproteinization, dialysis, purified by DEAE and Sephadex G-100 column chromatography	≥98%	Partial. (Molecular weight and glycosidic bond types provided)	Li et al., 2025
Astragalus Polysaccharide (APS)	Astragalus	APS purchased from Kamai Shu Biotechnology (extraction method not described)	0.985	Yes. (Monosaccharide composition and molecular weight provided)	Tan et al., 2020
Angelica Polysaccharide (AP)	Angelica sinensis	Extraction method not described	95% purity	No. (Monosaccharide composition, molecular weight, linkage types not	Xue et al., 2019

Polysaccharide Name (Abbreviation)	Source	Extraction Method	Purity	Structural Elucidation Status	Reference
				provided)	
Salvia miltiorrhiza Polysaccharide (SMP1)	Salvia miltiorrhiza Bunge	Microwave-assisted extraction, purified by DEAE Sepharose Fast Flow and Sephadex G-100 chromatography	Purity not explicitly stated; purification steps confirmed	Yes. (Monosaccharide composition, molecular weight, FT-IR, NMR provided)	Meng et al., 2022
Lycium barbarum Polysaccharides (LBP)	Lycium barbarum	Extraction method not described	Not specified	No. (Monosaccharide composition, molecular weight, linkage types not provided)	Xu et al., 2024
Glochidion eriocarpum Polysaccharide (GPS)	Glochidion eriocarpum Champ.	Extraction method not detailed	Not specified	No. (Detailed structural information like monosaccharide composition, linkage types, molecular weight not provided in main text)	Cao et al., 2024
Codonopsis pilosula Polysaccharides (CPPs)	Codonopsis pilosula	Extraction method not detailed	Not specified	Partial. (Monosaccharide composition provided; lacks molecular weight, linkage	Hu et al., 2021

Polysaccharide Name (Abbreviation)	Source	Extraction Method	Purity	Structural Elucidation Status	Reference
				details)	
RP01-1	Roots of Polygala tenuifolia Willd.	Water extraction, alcohol precipitation, purified by DEAE Sepharose FF and Sephacryl S-200 column chromatography	Homogeneous polysaccharide (single peak by HPGPC); purity value not specified	Yes. (Monosaccharide composition, molecular weight, methylation analysis, NMR provided)	Zeng et al., 2020
PTP70-2	Polygala tenuifolia	Extraction method not detailed	Not specified	No. (Monosaccharide composition, linkage types, molecular weight not provided)	Chen et al., 2022
ATP50-3	Acorus tatarinowii	Water extraction (80°C), 50% ethanol precipitation, purified by DEAE-52 cellulose column (eluted with 0.1 M NaCl) and Sephadex G-75 gel filtration	Homogeneous polysaccharide (single symmetrical peak by HPGPC)	Yes. (Comprehensive structural characterization via monosaccharide composition, FT-IR, GC-MS, NMR)	Zhong et al., 2020
ARP-1	Aster tataricus L. f. (Asteris Radix et Rhizoma)	Water extraction (100°C), 80% ethanol precipitation, Sevag deproteinization, dialysis (MWCO 10 kDa), purified by DEAE-52	Carbohydrate content 97.9%; UV spectrum showed no	Yes. (Comprehensive characterization: Monosaccharide composition, molecular	Zhu et al., 2022

Polysaccharide Name (Abbreviation)	Source	Extraction Method	Purity	Structural Elucidation Status	Reference
		cellulose (water elution) and Sephadex G-100 columns	protein/nucleic acid absorption	weight, FT-IR, methylation analysis, NMR)	
PGP1	Platycodon grandiflorum	Water extraction (boiling), ethanol precipitation, Sevag deproteinization, dialysis (MWCO 3500 Da), purified by DEAE-cellulose column (water elution) and Sephacryl S-400/HR columns	Polysaccharide content 95.6%; protein negative (Bradford assay and UV spectrum)	Partial. (Monosaccharide composition and molecular weight provided; lacks linkage types and detailed configuration)	Sheng et al., 2017
LICP009-3F-1a	Fruits of Lycium barbarum L.	High-speed shear-assisted extraction and cascade membrane separation, deproteinization, decolorization, ion-exchange resin separation, purified by Sephadex G-50 column	Sugar content 98.4%; no protein or uronic acid detected	Yes. (Detailed characterization: Monosaccharide composition, FT-IR, XRD, TGA, SEM, methylation analysis, NMR)	Li et al., 2025
Cistanche deserticola Polysaccharides (CDP)	Cistanche deserticola	Extraction method not detailed; purchased from Yuanye Biological Technology Co.	>98% purity	No. (Monosaccharide composition, molecular weight, linkage types not provided)	Liu et al., 2018

**Polysaccharide Name (Abbreviation)**	**Source**	**Extraction Method**	**Purity**	**Structural Elucidation Status**	**Reference**
Chinese Angelica Polysaccharid e (CAP)	Angelica sinensis	Extraction method not detailed; purchased from Yongye Bio-engineering Co., Ltd.	98% purity	No. (Monosaccharide composition, molecular weight, linkage types not provided)	Xie et al., 2018
Lycium barbarum Polysaccharide (LBP)	Lycium barbarum	Extraction method not detailed; purchased from Beijing Solarbio Science & Technology Co., Ltd.	>50% purity	No. (Monosaccharide composition, molecular weight, linkage types not provided)	Yu et al., 2018
Coptis chinensis Polysaccharide (CCP)	Dried rhizomes of Coptis chinensis (Chinese Goldthread)	Detailed steps: Defatting, water extraction, alcohol precipitation, deproteinization, column chromatography	Sugar content 94.9%; no protein or nucleic acid detected; purity value not specified	Partial. (Molecular weight and monosaccharide composition provided; linkage types not specified)	Li et al., 2019
Hedysari Radix Polysaccharide (HRP)	Hedysarum polybotrys Hand-Mazz	HRP purchased from Pythonbio (wkq20041508)	Purity not specified	No. (Monosaccharide composition, molecular weight, linkage types not provided)	Yang et al., 2022
Polysaccharides from Basella	Basella alba (Plant identified by Wen-Hwa	Water extraction (90°C, 2 h), ethanol precipitation (95%, 3:1	Purity value not specified;	Partial. (Monosaccharide composition and molecular	Hou et al.,

Polysaccharide Name (Abbreviation)	Source	Extraction Method	Purity	Structural Elucidation Status	Reference
alba (PPV-6)	Lin)	ratio), lyophilization	purification steps confirmed	weight provided; lacks details on linkage types, branching configuration)	2024
Corydalis yanhusuo Polysaccharide (CYP)	Dried roots of Corydalis yanhusuo	Detailed steps: Defatting, water extraction, alcohol precipitation, deproteinization, column chromatography	Sugar content 96.85%; no protein or nucleic acid detected; purity value not specified	Yes. (Molecular weight, monosaccharide composition, linkage types provided; confirmed by methylation analysis and NMR)	He et al., 2020
JCS1 (Natural Polysaccharide)	Stems of Dendrobium nobile Lindl (Orchidaceae)	Hot water extraction, ethanol precipitation, purified by anion-exchange chromatography (Q Sepharose Fast Flow column)	Homogeneous by HPGPC; contains 6.6% protein (Lowry method); no uronic acid detected	Yes. (Detailed characterization by methylation analysis, FTIR, NMR (HSQC, HMBC), partial acid hydrolysis, etc.)	Jin et al., 2017

Supplementary Table 3 Pharmacological Research Conditions.

Polysaccharide Name（ref）	Experimental Model	Dose Range	Minimum Effective Concentration	Control Group Setup	Treatment Duration
Potentilla anserine L. polysaccharide (PAP) (Cheng et al., 2021)	N2a cells, primary neurons, BALB/c mice	In vitro: 6.25, 25 mg/L; In vivo: 1.5 mg/kg	In vitro: 6.25 mg/L (some parameters); 25 mg/L (significant effects)	Control group, Cd group, PAP group, PAP+Cd group, LY/3-MA inhibitor groups	In vitro: 24 h; In vivo: 28 days (Cd exposure), PAP pretreatment for 2 days, total 30 days
Rehmannia glutinosa polysaccharide (RGP) (Yang et al., 2024)	C57BL/6 mice (in vivo), HT-22 hippocampal neuronal cells (in vitro)	In vivo: 100, 200, 400 mg/kg (oral, every other day); In vitro: 62.5, 125, 250 µg/mL	In vitro: 62.5 µg/mL (some parameters); 250 µg/mL (significant effects); In vivo: 400 mg/kg (most effective)	Control group, CCL/Light group, different RGP dose treatment groups, AKT inhibitor (Perifosine) group	In vivo: 4 weeks (CCL exposure), RGP administered every other day; In vitro: 24 h
Lycium barbarum polysaccharide (LBP) (Li et al., 2023)	C57BL/6J mouse MCAO model; In vitro primary neuron oxygen-glucose deprivation/reperfusion (OGD/R) model	In vitro NSC pretreatment: 50, 100, 500 µg/mL; In vivo therapy: intravenous injection of EVs (dose based on particle count: ~1-2.17 × 10^9 particles/mL)	In vitro: 50 µg/mL (some parameters); 500 µg/mL (most effective)	Sham group, MCAO/PBS group, NSC-EV group, different LBP-pretreated NSC-EV groups, GW4869 (EV generation inhibitor) group, anti-miR-133a-3p oligonucleotide group	In vitro NSC pretreatment: 72 h; In vivo: EVs injected starting 6h after reperfusion, twice daily for 3 days; behavioral assessments up to 28 days
Gastrodia elata	C57BL/6J mouse MCAO	In vivo: 0.5, 1, 2	In vivo: 1 mg/kg	In vivo: Sham, Vehicle, different	In vivo: Administration for

Polysaccharide Name（ref）	Experimental Model	Dose Range	Minimum Effective Concentration	Control Group Setup	Treatment Duration
Neutral Polysaccharide (NPGE) (Zhang et al., 2024)	model; HT22 cell OGD/R model	mg/kg (i.p.); In vitro: 100, 250, 500 µg/mL	(significant effect); In vitro: 250 µg/mL (some parameters), 500 µg/mL (most effective)	NPGE dose groups (0.5, 1, 2 mg/kg), NPGE (1 mg/kg) + Brusatol (NRF2 inhibitor) group; In vitro: Control, Vehicle, different NPGE dose groups (100, 250, 500 µg/mL), NPGE (500 µg/mL) + Brusatol group, Erastin (ferroptosis inducer) group, Erastin + NPGE group	3 days post-MCAO surgery; In vitro: Treatment for 24 h after OGD/R
Glycyrrhiza Polysaccharide (GP) (Du et al., 2024)	In vitro: Human recombinant CYP46A1 enzyme; h-CYP46A1-Neuro-2a cells; In vivo: Wistar rats	In vitro: 0.1-5 mg/mL (inhibitory activity screening), 0.3-1.5 mg/mL (kinetic analysis), 0.3-0.9 mg/mL (cell experiments); In vivo: 50 mg/kg (i.p.)	In vitro: IC_{50} = 0.6621 mg/mL; K_i = 0.7003 mg/mL; In vivo: 50 mg/kg (significantly reduced plasma and brain 24S-HC levels after 30 days)	In vitro: Solvent control, positive inhibitor control (Soticlestat), different GP concentration groups; In vivo: Saline control group, GP administration group (50 mg/kg)	In vitro: Enzyme reaction 10 min; Cell treatment 24 h; In vivo: Once daily for 30 days
Lycium barbarum polysaccharide (LBP) (Yang et al., 2023)	In vivo: C57BL/6 mouse LAN model; In vitro: HT-22 hippocampal neuronal cell blue light LED model	In vivo: 50, 100, 200 mg/kg (oral); In vitro: 7.81, 15.62, 31.25, 62.5, 125, 250, 500 µg/mL	In vitro: 15.62 µg/mL (some parameters); 125 µg/mL (significant effects); In vivo: 200 mg/kg (most effective)	In vivo: Control, LAN, LAN + 50 mg/kg LBP, LAN + 100 mg/kg LBP, LAN + 200 mg/kg LBP groups; In vitro: Control, Light, Light + different LBP concentrations, Light + LBP + ML385 (Nrf2 antagonist)	In vivo: 4 weeks; In vitro: 24 h

Polysaccharide Name（ref）	Experimental Model	Dose Range	Minimum Effective Concentration	Control Group Setup	Treatment Duration
				groups	
Momordica charantia polysaccharides (MCPs) (Hu et al., 2020)	In vivo: SD rat MCAO model; In vitro: C17.2 neural stem cell line, primary rat cortical neural stem cells (E16-NSC), glutamate stimulation to mimic ischemia/reperfusion injury	In vivo: 200 mg/kg (oral gavage); In vitro: 1.0, 2.0, 3.0, 5.0, 10.0, 20.0 μg/mL (screening under physiological conditions), 2.0, 5.0, 10.0 μg/mL (mechanistic studies under pathological conditions)	In vitro: 2.0 μg/mL (showed promoting effect on neuronal differentiation in glutamate injury model); In vivo: 200 mg/kg (effective after 14 days)	In vivo: Sham, MCAO, MCAO+MCPs groups; In vitro: Control, Glutamate injury, different MCPs treatment, SIRT1 activator (Resveratrol), SIRT1 inhibitor (Nicotinamide), SIRT1 siRNA transfection groups	In vivo: Daily administration for 14 days post-MCAO; In vitro: Glutamate stimulation for 30 min, then drug-containing medium for 24 h (differentiation markers) or longer (mechanistic studies)
Astragalus polysaccharide (APS) (Jia et al., 2022)	In vitro: HAPI cells (rat microglial cell line) and OGD model; In vivo: SD rat MCAO model	In vitro: APS 100 mg/L; In vivo: APS 22.5 mg/kg and 45 mg/kg	In vitro: 100 mg/L; In vivo: 45 mg/kg (more significant effect)	Control, MCAO, MCAO+normal saline, MCAO+APS low dose, MCAO+APS high dose groups	In vitro: APS treatment for 48 h (after ATP or OGD); In vivo: i.p. injection of APS, once daily for 1, 3, or 5 days
SCP2-1 (Xu et al., 2020)	In vitro: BV2 microglial cells; In vivo: KM mice intracerebroventricular LPS	In vitro: 6.25, 12.5 μg/mL; In vivo: 28, 56 mg/kg (oral)	In vitro: 6.25 μg/mL (some parameters); 12.5 μg/mL (significant effects); In	In vitro: Control, LPS, LPS+SCP2-1 (6.25, 12.5 μg/mL), LPS+SCP2-1+siLRP-1 groups; In vivo: Control, LPS, LPS+SCP2-1	In vitro: Pretreatment 2h, LPS stimulation 24h; In vivo: 3 days recovery post-LPS injection, then 3

Polysaccharide Name（ref）	Experimental Model	Dose Range	Minimum Effective Concentration	Control Group Setup	Treatment Duration
	injection model		vivo: 28 mg/kg (some parameters), 56 mg/kg (more effective)	low dose, LPS+SCP2-1 high dose, LPS+donepezil groups	weeks administration
Potentilla anserine L. polysaccharide (PAP) (Cheng et al., 2022)	In vitro: N2a cells, SH-SY5Y cells; In vivo: BALB/c mice	In vitro: 6.25, 25 mg/L; In vivo: 1.5 mg/kg (i.p.)	In vitro: 6.25 mg/L (some parameters); 25 mg/L (significant effects); In vivo: 1.5 mg/kg (effective)	In vitro: Control, Cd, PAP (6.25 mg/L) + Cd, PAP (25 mg/L) + Cd groups; In vivo: Control, PAP, Cd, PAP+Cd groups	In vitro: Pretreatment 1h, $CdCl_2$ treatment 24h; In vivo: PAP injection 30 days, $CdCl_2$ injection 28 days
Maca polysaccharide (MP) (Zhou et al., 2022)	In vivo: ICR mouse D-gal-induced aging model; In vitro: SH-SY5Y cell H_2O_2-induced oxidative damage model	In vivo: 75, 150, 300 mg/kg (oral); In vitro: 25, 50, 100 µg/mL	In vivo: 75 mg/kg (some parameters), 300 mg/kg (most effective); In vitro: 25 µg/mL (some parameters), 100 µg/mL (most effective)	In vivo: Control, D-gal, D-gal + MP (75, 150, 300 mg/kg) groups; In vitro: Control, H_2O_2, H_2O_2 + MP (25, 50, 100 µg/mL) groups	In vivo: 8 weeks; In vitro: MP pretreatment 24 h, H_2O_2 treatment 6 h
Lycium barbarum polysaccharide (LBP) (Shi et al., 2017)	In vivo: Wistar rat four-vessel occlusion (4-VO) global cerebral ischemia model; In vitro: Primary rat cortical neuron OGD model	In vivo: 20 mg/kg (oral gavage); In vitro: 100 mg/L	In vivo: 20 mg/kg; In vitro: 100 mg/L	In vivo: Sham, Vehicle, Pre-LBP, Post-LBP, LBP-LBP groups; In vitro: Control, OGD, OGD+LBP, OGD+LBP+D-serine, OGD+LBP+NVP-AAM077 groups	In vivo: LBP pretreatment 1 week and/or post-treatment 1 week; In vitro: OGD 4 h, reperfusion 24 h

Polysaccharide Name（ref）	Experimental Model	Dose Range	Minimum Effective Concentration	Control Group Setup	Treatment Duration
Lonicera japonica polysaccharide (LJP) (Wang et al., 2021)	In vivo: Male Kunming mouse LPS-induced cognitive impairment model	30, 100 mg/kg (oral gavage)	30 mg/kg (some parameters); 100 mg/kg (more significant effect)	Control, LPS model, LPS + LJP 30 mg/kg, LPS + LJP 100 mg/kg groups	7 days
Lycium barbarum polysaccharide (LBP) (Wang et al., 2018)	In vivo: Male C57BL/6 mouse MPTP-induced Parkinson's disease model	100, 200 mg/kg (i.p.)	100 mg/kg (some parameters); 200 mg/kg (more significant effect)	Control, PD model (MPTP), PD + LBP-100 mg/kg, PD + LBP-200 mg/kg groups	16 days (starting from day 5 after the last MPTP injection, until day 21)
Morinda officinalis polysaccharide (MOP) (Dong et al., 2025)	In vitro: SH-SY5Y cell MPP^+ model; In vivo: C57BL/6 mouse MPTP model	In vitro: 10, 20, 40 µg/mL; In vivo: 10, 20, 40 mg/kg	In vitro: 10 µg/mL (some parameters); In vivo: 10 mg/kg (some parameters)	In vitro: Control, MPP^+, MPP^+ + 10 µg/mL MOP, MPP^+ + 20 µg/mL MOP, MPP^+ + 40 µg/mL MOP groups; In vivo: Control, MPTP, MPTP + 10 mg/kg MOP, MPTP + 20 mg/kg MOP, MPTP + 40 mg/kg MOP groups	In vitro: MOP pretreatment 24 h, MPP^+ treatment 48 h; In vivo: MOP injection 15 days, MPTP injection last 5 days
Dendrobium Nobile Polysaccharides (DNP) (Ming et	In vivo: SD rat permanent bilateral common carotid artery occlusion (VD) model	100 mg/kg/d (oral gavage)	100 mg/kg/d (effective)	Sham, VD model, VD + DNP (100 mg/kg) groups	3 months

Polysaccharide Name（ref）	Experimental Model	Dose Range	Minimum Effective Concentration	Control Group Setup	Treatment Duration
al., 2023)					
Astragalus polysaccharides (APS) (Ye et al., 2021)	In vivo: C57BL/6 mouse cuprizone (CPZ)-induced demyelination model; In vitro: C17.2 neural stem cell (NSC) differentiation model	In vivo: 500 mg/(kg·day) (oral gavage); In vitro: 0.1, 1, 10, 100 µg/mL, 1 mg/mL	In vivo: 500 mg/(kg·day) (effective); In vitro: 0.1 µg/mL (some parameters), higher concentrations more effective	In vivo: Normal (normal diet), CPZ (CPZ diet + saline), APS (CPZ diet + APS) groups; In vitro: Control (differentiation medium + PBS), APS concentration groups (differentiation medium + different APS concentrations)	In vivo: APS treatment for 3 weeks (weeks 5 to 7); In vitro: APS intervention for 4 days
Astragalus polysaccharides (APS) (Zhao et al., 2024)	In vivo: EAE mouse model (C57BL/6 female); In vitro: C17.2 neural stem cell line co-cultured with $CD8^+$ T cells	In vivo: APS 500 mg/kg/d (oral); In vitro: APS not used directly, studied indirectly via $CD8^+$ T cell co-culture	In vivo: 500 mg/kg/d (significant effect); In vitro: No direct APS concentration provided	In vivo: Naïve, EAE + saline, EAE + APS groups; In vitro: Various co-culture groups (e.g., $CD8^+$ T cells + NSCs, neutralizing antibody groups)	In vivo: From immunization day to day 18 (acute phase); In vitro: $CD8^+$ T cells co-cultured with NSCs for 72 h
Dendrobium officinale polysaccharide (DOP) (Sun et al., 2022)	In vivo: Male C57BL/6J mice, constant darkness (CD)-induced circadian rhythm disruption (CRD) model	In vivo: 200 mg/kg/d (oral gavage)	In vivo: 200 mg/kg/d (significantly improved cognition, microbiota, inflammation, etc.)	CT (normal light/dark cycle + saline), CD (constant darkness + saline), DOP (constant darkness + 200 mg/kg/d DOP) groups	4 weeks

Polysaccharide Name（ref）	Experimental Model	Dose Range	Minimum Effective Concentration	Control Group Setup	Treatment Duration
Dendrobium officinale polysaccharide (DOP) (Fu et al., 2025)	In vivo: Male APP/PS1 transgenic AD mice (6 months old); Normal control: C57BL/6J mice	In vivo: 400 mg/kg/d (oral gavage)	In vivo: 400 mg/kg/d (significantly improved cognition, neuroinflammation, metabolomic and proteomic indicators)	NC (C57BL/6J + saline), AD (APP/PS1 + saline), DOP (APP/PS1 + 400 mg/kg/d DOP) groups	6 weeks
PSP-1 (Luo et al., 2022)	In vivo: SxFAD transgenic AD mouse model	30 mg/kg (oral)	Not specified	WT control, SxFAD+Vehicle, SxFAD+PSP-1 groups	3 months
Sea buckthorn polysaccharide (SBP) (Lan et al., 2023)	In vivo: Male C57BL/6 mouse high-fat diet-induced cognitive dysfunction model	0.1% SBP (w/w) incorporated into feed	Not specified	RC (regular chow), HFD (high-fat diet), HFD+SBP (high-fat diet + 0.1% SBP) groups	12 weeks
Fagopyrum esculentum polysaccharide (FEP) (Liu et al., 2024)	In vivo: SD rat $AlCl_3$-induced AD model	200 mg/kg (FEPL), 400 mg/kg (FEPH)	200 mg/kg (effective for some parameters)	Control, $AlCl_3$, $AlCl_3$+FEPL, $AlCl_3$+FEPH groups	49 days
Gastrodia elata	C57BL/6 mice,	200, 400, 600	200 mg/kg (partially	Con, GEP, MPTP, MPTP+L-DOPA,	3 weeks (preventive

**Polysaccharide Name（ref）**	**Experimental Model**	**Dose Range**	**Minimum Effective Concentration**	**Control Group Setup**	**Treatment Duration**
polysaccharide (GEP) (Gan et al., 2024)	MPTP-induced PD model	mg/kg/day (oral)	effective), 400-600 mg/kg (significant effects)	MPTP+GEP-L/M/H groups	administration 1 week + 2 weeks post-MPTP)
Astragalus membranaceus Bunge polysaccharides (APS) (Liu et al., 2025)	In vivo: C57BL/6 mice, LPS-induced neuroinflammation model	APS: 200 mg/kg/day (oral, 30 days); LPS: 7.5 mg/kg (i.p., last day)	Minimum effective concentration not specified, but 200 mg/kg was effective	Control groups: CK (no treatment), LPS (LPS only), APS (APS + LPS)	APS pretreatment 30 days, LPS injected on last day
Corydalis yanhusuo Polysaccharide (CYP) (Fang et al., 2023)	In vivo: C57BL/6 mice, chronic unpredictable mild stress (CUMS)-induced depression model	CYP: 200 mg/kg/day (oral gavage, 4 weeks); Positive control: Fluoxetine 20 mg/kg/day	200 mg/kg/day (effective in behavioral and biochemical indicators)	Control (no stress + vehicle), Model (CUMS + vehicle), CYP-treated (CUMS + 200 mg/kg CYP), Fluoxetine-treated (CUMS + 20 mg/kg Fluoxetine) groups	CUMS modeling 8 weeks, drug intervention during the last 4 weeks
Schisandra polysaccharide (SCP) (Liu et al., 2019)	In vivo: Aβ_{25-35}-induced AD rat model	In vivo: 38.15 mg/kg/day (equivalent to 545 mg/kg/day Schisandra)	In vivo: 38.15 mg/kg/day (effective, but no gradient dose testing)	In vivo: Sham operation (SHO), AD model (ADM), positive drug donepezil (PDD, 0.91 mg/kg), SCP groups	In vivo: 56 days

Polysaccharide Name（ref）	Experimental Model	Dose Range	Minimum Effective Concentration	Control Group Setup	Treatment Duration
Lycium barbarum polysaccharide (LBP) (Song et al., 2022)	In vivo: MPTP-induced silkworm PD model	In vivo: 50, 100, 200 mg/kg (oral, via mulberry leaf application)	In vivo: 50 mg/kg (effective for some behavioral and molecular indicators)	In vivo: Control, MPTP model, MPTP + LBP (50, 100, 200 mg/kg), MPTP + LD-CD (4, 8, 20 mM positive drug) groups	In vivo: Administration from the 5th instar to the 5th day of the 5th instar (specific days not clearly stated)
Aronia melanocarpa polysaccharide (AMP) (Zhao et al., 2021)	In vivo: D-galactose-induced aging mouse model	In vivo: 100, 200 mg/kg (oral gavage)	In vivo: 100 mg/kg (effective for some parameters), 200 mg/kg (more significant effect)	In vivo: Normal, D-Gal model, D-Gal + AMP low dose (100 mg/kg), D-Gal + AMP high dose (200 mg/kg) groups	In vivo: D-Gal induction 12 weeks (i.p. every 3 days), AMP intervention last 6 weeks (oral daily)
Potentilla anserina L polysaccharide (PAP) (Shi et al., 2020)	In vivo: BALB/C mice (normobaric hypoxia, acute hypoxia); Wistar rats (acute hypobaric hypoxia-induced high-altitude cerebral edema model). In vitro: Not performed.	Mice: 150, 300, 500 mg/kg (oral); Rats: 100, 200, 400 mg/kg (oral).	Mice: 150 mg/kg (normobaric hypoxia survival time); Rats: 200 mg/kg (brain water content, oxidative stress and inflammation indicators).	In vivo: Normoxia Control (NC), Hypobaric Hypoxia Model (HHM), PAP low/medium/high dose, Dexamethasone (Dex) positive control groups.	In vivo: Administration for 3 days, followed by hypobaric hypoxia exposure for 72 hours.
Scrophularia ningpoensis	In vivo: SD rat middle cerebral	10, 20, 40 mg/kg	20 mg/kg (showed significant effects on	Sham, MCAO model, Nimodipine positive control, SNP2-A (10, 20, 40	i.v. injection, once daily for

Polysaccharide Name（ref）	Experimental Model	Dose Range	Minimum Effective Concentration	Control Group Setup	Treatment Duration
polysaccharide (SNP2-A) (Ma et al., 2019)	artery occlusion model.	(i.v.)	neurological deficit, cerebral infarction volume, brain water content, oxidative stress and inflammation indicators)	mg/kg) administration groups	7 days (pretreatment)
Lycium barbarum polysaccharide (LBP) (Zhao et al., 2017)	ICR mouse MCAO model	10, 20, 40 mg/kg (oral gavage)	20 mg/kg (some parameters), 40 mg/kg (significant effects)	Sham, Vehicle, LBP different dose, Nimodipine groups	Oral gavage of LBP or Nimodipine once daily for 7 consecutive days, MCAO surgery performed after administration on day 7, multiple tests started 24h after reperfusion, some behavioral tests (e.g., Morris water maze) lasted until day 7 after reperfusion.
Schisandra Chinensis Fructus polysaccharide (SCP) (Xu et al.,	KM mouse $A\beta_{1-42}$-induced AD model	260 mg/kg (oral)	260 mg/kg (improved behavior and biochemical indicators)	Control, Model, SCP, Donepezil groups	28 days (administration after modeling), followed by behavioral tests and various indicator

Polysaccharide Name（ref）	Experimental Model	Dose Range	Minimum Effective Concentration	Control Group Setup	Treatment Duration
2019)					assessments
Epimedium brevicornum polysaccharide (EbPS-A1) (Xiang et al., 2017)	C. elegans HA759 (polyQ neurotoxicity model)	1-2 mg/mL (treatment in S medium)	1 mg/mL (some parameters), 2 mg/mL (significant effects)	Untreated control, Polysaccharide-treated groups	Treatment started from L1 larval stage in medium containing polysaccharide.<br>Chemosensory behavior, ROS levels, antioxidant enzyme activity, MDA content: measured after 3 days treatment.<br>Polyglutamine aggregation and body bending rate: measured at 24, 48, 72 h, and on adult days 1, 3, 5.<br>Lifespan assay: treatment from young adulthood until death.<br>Paraquat resistance assay: pretreatment of young adult worms with polysaccharide for 24 h before paraquat exposure.
Polygonatum sibiricum	C. elegans N2, CL4176, EG1285, GR1366 etc.	20, 100, 200 μg/mL	20 μg/mL (some parameters), 100-200	Control, FBI, PSPE pretreatment	PSPE pretreatment until L3 stage, FB1 exposure for 24

Polysaccharide Name（ref）	Experimental Model	Dose Range	Minimum Effective Concentration	Control Group Setup	Treatment Duration
polysaccharide extract (PSPE) (Zhang et al., 2022)	(FBI-induced neurotoxicity)	(pretreatment)	μg/mL (significant effects)	groups	h
Cyclocarya paliums polysaccharide (CPP) (Lin et al., 2020)	Caenorhabditis elegans (C. elegans)	13.75 μg/mL	13.75 μg/mL	Control, Positive control (APS)	Treatment start point: From egg stage.<br>Treatment duration varied by endpoint:<br>Stress resistance, ROS, biochemical indicators, gene expression, DAF-16 localization, SOD-3::GFP: measured after 3 days treatment (early adulthood).<br>Lifespan, PolyQ paralysis assay: Lifelong treatment from eggs.<br>Aβ-induced paralysis assay: Treatment for 36 h, then temperature upshift to induce expression and continue treatment until paralysis.<br>Age pigment fluorescence, physical ability: Assessed

Polysaccharide Name（ref）	Experimental Model	Dose Range	Minimum Effective Concentration	Control Group Setup	Treatment Duration
					on days 3, 7, 11 post-treatment. Fertility, Growth rate: Treated and assessed during development and reproductive stages.
Astragalus polysaccharide (APS) (Sun et al., 2020)	SD rat cerebral thrombosis model	200 mg/kg (APS and APS-nano)	200 mg/kg (APS-nano more effective than APS)	NC, Model, APS, APS-nano groups	14 days (daily oral gavage)
Momordica charantia polysaccharide (MCP) (Yue et al., 2023)	SD rat D-galactose aging model	100 mg/kg, 300 mg/kg	100 mg/kg (some parameters), 300 mg/kg (more significant effect)	Control, DGAM, Piracetam, MCP two dose groups	Daily subcutaneous D-galactose (250 mg/kg), oral administration 1 hour later, once daily for 6 weeks
Hippophae rhamnoides L. polysaccharide I (HRPI) (Zhao et al., 2023)	BALB/c mouse AD model (D-gal + $AlCl_3$ induced)	13, 26, 52 mg/kg	26 mg/kg (most effective)	Control, Model, Donepezil, HRPI three dose groups	Daily oral $AlCl_3$ (20 mg/kg) and i.p. D-galactose (120 mg/kg) for model induction, concurrent daily oral administration for 60 days; water maze test at the

Polysaccharide Name（ref）	Experimental Model	Dose Range	Minimum Effective Concentration	Control Group Setup	Treatment Duration
					end
Polysaccharides from Polygonatum cyrtonema Hua (PSP) (Xie et al., 2024)	C57BL/6 mouse SPS-induced PTSD model	200, 400, 800 mg/kg (oral gavage)	400 mg/kg (most significant behavioral improvement)	Control, SPS, PSP different dose, PRX positive drug groups	14 days
Angelica sinensis polysaccharide (ASP) (Xu et al., 2021)	SD rat cerebral ischemia-reperfusion injury (CIRI) model	10 mg/kg (i.p.)	10 mg/kg (only dose tested)	Sham, CIRI, ASP, ASP+LY294002 groups	Once daily for 2 consecutive weeks
Brassica rapa polysaccharide (BRP) (Zou et al., 2022)	SD rat acute high-altitude hypoxia (AHH) model	38, 75, 150 mg/kg (oral)	150 mg/kg (most effective)	Control, AHH, Hongjingtian oral liquid, BRP different dose groups	Preventive administration: Daily gavage started 7 days before AHH exposure, last administration 1 h before 24 h AHH exposure
Angelica sinensis polysaccharides (ASP) (Du et al.,	SD rat $A\beta_{25-35}$-induced AD model	50 mg/kg (oral gavage, bid)	50 mg/kg (only dose tested)	Sham, Model, Donepezil, ASP, Model+K252a, ASP+K252a groups	Administration started 3 days after $A\beta_{25-35}$ injection, twice daily for 4 weeks

Polysaccharide Name（ref）	Experimental Model	Dose Range	Minimum Effective Concentration	Control Group Setup	Treatment Duration
2020)					
Dendrobium officinale polysaccharide (DP) (Chen et al., 2023)	C57BL/6J mice HFD-induced diabetic model	200 mg/kg/day (only dose)	200 mg/kg	CON (control), HFD (high-fat diet), MET (metformin, positive control), DP (DP treatment) groups, n=12	Continuous gavage for 8 weeks
Codonopsis pilosula polysaccharides (CPPs) (Cai et al., 2025)	APP/PS1 transgenic mice (Alzheimer's disease model)	75, 150, 300 mg/kg/day (oral)	75 mg/kg, 300 mg/kg most effective	CON (C57BL/6J control), MOD (APP/PS1 model), L-CPPS (75 mg/kg), M-CPPS (150 mg/kg), H-CPPS (300 mg/kg) groups, n=10	Continuous gavage for 28 days
Polygonatum cyrtonema polysaccharide (PCP) (Li et al., 2025)	C57BL/6 mice MPTP-induced Parkinson's disease model	60, 100 mg/kg/day (oral)	60 mg/kg, 100 mg/kg more effective	Control, MPTP, L-DOPA (75 mg/kg, positive control), PCP (60 mg/kg), PCP (100 mg/kg) groups, n=8-10	PCP oral 14 days, MPTP i.p. from day 4 to day 10
Astragalus polysaccharide (APS) (Tan et al.,	PC12 cell 6-OHDA-induced Parkinson's disease model	50, 100, 200 μM	50 μM, 200 μM most effective	Untreated control, 6-OHDA (100 μM), APS (50, 100, 200 μM), PI3K	Co-treatment with 6-OHDA for 24 h

Polysaccharide Name（ref）	Experimental Model	Dose Range	Minimum Effective Concentration	Control Group Setup	Treatment Duration
2020)				knockdown (KD) groups	
Angelica polysaccharide (AP) (Xue et al., 2019)	Rat neural stem cell (NSC) hypoxia model (in vitro)	10, 30, 50, 70, 100 µM (cell viability); 50 µM (main experiments)	50 µM	Control, Hypoxia, Hypoxia+AP groups (lacks positive control)	NSCs pretreated with AP for 24 h, then hypoxia stimulation for 8 h
Salvia miltiorrhiza polysaccharide (SMP1) (Meng et al., 2022)	PC12 cell oxygen-glucose deprivation/reoxygenation (OGD/R) model (in vitro)	5, 10, 20, 40, 60, 80, 100, 120, 160 µg/mL (cell viability); 20, 40, 60 µg/mL (further experiments)	20 µg/mL	Control, Vehicle, ML385, SMP1 dose groups, SMP1+ML385, Eda positive drug groups	Cells pretreated with SMP1, then OGD 4 h, reoxygenation 24 h (with drug); ML385 added 2 h before OGD/R
Lycium barbarum polysaccharides (LBP) (Xu et al., 2024)	PC-12 cell combined NP and OP exposure model (in vitro)	LBP: 62.5, 125, 250 µg/mL; NP+OP: 1, 2, 4, 8 µg/mL	62.5 µg/mL	Control, NP+OP, LBP intervention dose, p38 inhibitor groups (lacks positive control)	LBP pretreatment 24 h, then co-treatment with NP+OP for 24 h; p38 inhibitor pretreatment 2 h
Glochidion eriocarpum polysaccharide (GPS) (Cao et al.,	PC12 cells	20–200 µg/mL (MTT assay), 80 µg/mL (single-cell	10 µg/mL (significant neuroprotection in LDH assay)	Control group, STS group, GPS-treated groups; Positive control not mentioned in single-cell	LDH assay duration not specified; Single-cell experiments: GPS

Polysaccharide Name（ref）	Experimental Model	Dose Range	Minimum Effective Concentration	Control Group Setup	Treatment Duration
2024)		experiments)		experiments	pretreatment 3h
Codonopsis pilosula Polysaccharides (CPPs) (Hu et al., 2021)	PC12 cells, $A\beta_{1-40}$-induced model	25, 50, 100, 200, 300 μg/ml	25 μg/ml	Control, $A\beta_{1-40}$, $A\beta_{1-40}$+CPPs, CPPs, CD38 siRNA, CD38 siRNA+$A\beta_{1-40}$, CD38 siRNA+$A\beta_{1-40}$+CPPs groups	$A\beta_{1-40}$ treatment 24 h; CPPs co-treated with $A\beta_{1-40}$ for 24 h; CD38 siRNA transfection 24 h before treatment
RP01-1 (Zeng et al., 2020)	Aβ1-40-induced PC12 cells	0, 100, 500, 1000 μg/mL	100 μg/mL (some parameters), 500-1000 μg/mL more effective	Control, NGF (25 ng/mL, positive control), RP01-1 (100, 500, 1000 μg/mL)	72 h
PTP70-2 (Chen et al., 2022)	PC12 cells	3, 6, 12 μM	3 μM (some parameters), 6-12 μM more effective	Control, LPS, PTP70-2 (3, 6, 12 μM), TAK242 (TLR4 inhibitor), MINO (minocycline, positive control)	Pretreatment PTP70-2 (2 h) → LPS (1 μg/mL) stimulation (24 h for cytokines, 30 min for NF-κB nuclear translocation)
ATP50-3 (Zhong et al., 2020)	Lipopolysaccharide (LPS)-damaged BV2 microglial cells and neuroinflammation-injured	2.5, 5.0, 10 μM (in vitro)	2.5 μM (significantly inhibited LPS-induced iNOS and COX2	Control, LPS, ATP50-3 pretreatment, TAK242 (TLR4 inhibitor), LY294002 (PI3K inhibitor), MINO (minocycline,	BV2 cells pretreated with ATP50-3 for 2 h, then LPS (1 μg/mL) stimulation for 24 h (cytokines & proteins),

Polysaccharide Name（ref）	Experimental Model	Dose Range	Minimum Effective Concentration	Control Group Setup	Treatment Duration
	primary cortical neurons		protein expression)	positive control) groups	6 h (Akt phosphorylation), or 30 min (NF-κB nuclear translocation); Primary neurons treated with conditioned medium for 24 h
ARP-1 (Zhu et al., 2022)	Lipopolysaccharide (LPS)-induced proinflammatory BV2 cells	0.2, 1.0, 2.0 mg/mL (in vitro)	0.2 mg/mL (significantly increased H_2O_2-induced decreased SOD activity)	Natural (normal culture), Model (H_2O_2-induced), L-ARP-1 (0.2 mg/mL), M-ARP-1 (1.0 mg/mL), H-ARP-1 (2.0 mg/mL) groups; Positive control not mentioned in flow cytometry	PC12 cells pretreated with ARP-1 for 24 h, then 200 μM H_2O_2 stimulation for 4 h (for ROS, MDA, SOD detection); Cell viability detected 24 h after H_2O_2 stimulation
PGP1 (Sheng et al., 2017)	H_2O_2 induced oxidative stress in PC12 cells	50, 100, 200 μg/mL (in vitro)	50 μg/mL (significantly improved H_2O_2-induced decreased cell viability and LDH release)	Normal control (no H_2O_2), H_2O_2 injured group (0.5 mM H_2O_2), PGP1 groups (50, 100, 200 μg/mL); Positive control not mentioned	PC12 cells pretreated with PGP1 for 24 h, then 0.5 mM H_2O_2 stimulation for 12 h (for cell viability, apoptosis, morphology, ROS, LDH, SOD, MDA detection)
LICP009-3F-1a	PC12 cells, $CoCl_2$-induced	0–500 μg/mL (10, 50, 100, 200, 500	10 μg/mL ($p<0.05$)	Control, Hypoxia ($CoCl_2$), Different LICP009-3F-1a concentration	Pretreatment 24 h, then

Polysaccharide Name（ref）	Experimental Model	Dose Range	Minimum Effective Concentration	Control Group Setup	Treatment Duration
(Li et al., 2025)	hypoxia injury	μg/mL)		groups	$CoCl_2$ treatment 24 h
Cistanche deserticola polysaccharides (CDP) (Liu et al., 2018)	PC12 cells, OGD/RP-induced injury	0.05, 0.5, 5 μg/mL	0.05 μg/mL ($p<0.05$)	Control, Vehicle (OGD/RP), Nimo (nimodipine, 5 μg/mL), CDP different concentration groups	CDP added before reperfusion, reperfusion for 24 h
Chinese Angelica Polysaccharide (CAP) (Xie et al., 2018)	PC12 cells, LPS-induced injury	10, 100, 200 μg/mL	100 μg/mL ($p<0.01$)	Control, LPS, CAP different concentration, COX-1 overexpression groups	Pretreatment 24 h, then LPS treatment 12 h
Lycium barbarum polysaccharide (LBP) (Yu et al., 2018)	Primary hippocampal neurons, OGD/R-induced injury	15, 30, 60 μg/mL	15 μg/mL ($p<0.05$)	Control, OGD/R, LBP different concentration, LY294002 (PI3K inhibitor) groups	LBP added at the start of reperfusion, reperfusion for 24 h
Coptis chinensis polysaccharide (CCP) (Li et al., 2019)	PC12 cells, $A\beta_{25-35}$-induced neurotoxicity	5, 25, 50, 100, 200 μg/ml	5 μg/ml (significant protection at the lowest tested concentration in MTT and LDH assays)	Control, $A\beta_{25-35}$, $A\beta_{25-35}$+CCP (5,25,50,100,200 μg/ml), JNK inhibitor SP600125 groups, etc.	Pretreatment CCP 1–48 h (optimal at 24 h), followed by $A\beta_{25-35}$ exposure for 24 h

Polysaccharide Name（ref）	Experimental Model	Dose Range	Minimum Effective Concentration	Control Group Setup	Treatment Duration
Hedysari Radix Polysaccharide (HRP) (Yang et al., 2022)	HT22 cells, $A\beta_{25-35}$-induced model	10, 20, 40, 80, 160 µg/mL	20 µg/mL ($P<0.01$ in CCK-8)	Control, $A\beta_{25-35}$, CSH (40 µM Salidroside + 20 µg/mL HRP) groups	Pretreatment HRP or Salidroside 24 h, then co-treatment with $A\beta_{25-35}$ for 24 h (total 48 h)
Polysaccharides from Basella alba (PPV-6) (Hou et al., 2024)	Differentiated primary rat cortical neurons exposed to $A\beta_{25-35}$ or $A\beta_{1-42}$	PPV-6 50-500 µg/mL (in vitro)	50 µg/mL ($p<0.05$ vs $A\beta_{25-35}$ group in MTT assay)	Control (no treatment), Aβ ($A\beta_{25-35}$ or $A\beta_{1-42}$), Aβ+PPV-6 groups; Some experiments had post-treatment paradigm (e.g., Aβ pretreatment followed by PPV-6)	MTT assay and Hoechst staining 48 h; Mitochondrial function：24 h; Flow cytometry cell cycle analysis 16 h; Western blot: cyclin D1 detected at 8 h, PCNA and p-Histone H3 at 24 h, caspase-3 at 24 h; Post-treatment paradigm: $A\beta_{25-35}$ treatment 2 h then PPV-6 treatment 22 h
Corydalis yanhusuo polysaccharide (CYP) (He et al., 2020)	PC12 cells, $A\beta_{25-35}$-induced neurotoxicity	25, 50, 100 µg/ml	5 µg/ml (MTT showed significant effect starting at this concentration), effects more pronounced above 25µg/ml	Control, $A\beta_{25-35}$, $A\beta_{25-35}$+CYP (25,50,100 µg/ml) groups	Pretreatment CYP 24 h, followed by $A\beta_{25-35}$ exposure for 24 h

Polysaccharide Name（ref）	Experimental Model	Dose Range	Minimum Effective Concentration	Control Group Setup	Treatment Duration
JCS1 (Natural Polysaccharide) (Jin et al., 2017)	PC-12 cells (rat pheochromocytoma cell line)	YJCS1: 5.56, 33.33, 55.56 μM; JCS1 same concentrations ineffective	5.56 μM (lowest tested concentration)	Normal medium (negative control), Nerve Growth Factor (NGF, 25 ng/mL) as positive control	72 h

Reference

Cai, Y., Wang, X., Xiang, Y., Wang, Z., Long, Q., and Zeng, C. (2025). Codonopsis pilosula polysaccharides alleviate neuronal apoptosis induced by endoplasmic reticulum stress-activated PERK-ATF4-CHOP signaling in APP/PS1 mice. J. Alzheimers Dis. 106(1), 317-330. doi:10.1177/13872877251339484

Cao, L., Zhang, J., Li, M., Zhou, J., Liu, Y., Liu, C., et al. (2024). Single-Vesicle Electrochemistry Reveals Polysaccharide from Glochidion eriocarpum Champ. Regulates Vesicular Storage and Exocytotic Release of Dopamine. Anal. Chem. Published online September 11, 2024. doi:10.1021/acs.analchem.4c02493

Chen, H., Zhong, J., Li, J., Zeng, Z., Yu, Q., and Yan, C. (2022). PTP70-2, a novel polysaccharide from Polygala tenuifolia, prevents neuroinflammation and protects neurons by suppressing the TLR4-mediated MyD88/NF-κB signaling pathway. Int. J. Biol. Macromol. 194, 546-555. doi:10.1016/j.ijbiomac.2021.11.097

Chen, L., He, X., Wang, H., Fang, J., Zhang, Z., Zhu, X., et al. (2023). Dendrobium officinale polysaccharide prevents neuronal apoptosis via TET2-dependent DNA demethylation in high-fat diet-induced diabetic mice. Int. J. Biol. Macromol. 233, 123288. doi:10.1016/j.ijbiomac.2023.123288

Cheng, J., Liu, D., Zhao, L., Zhao, Q., Zhang, X., Wang, B., et al. (2021). Potentilla anserine L. polysaccharide inhibits cadmium-induced neurotoxicity by attenuating autophagy. Neurochem. Int. 147, 105045. doi:10.1016/j.neuint.2021.105045

Cheng, J., Zhao, L., Liu, D., Shen, R., and Bai, D. (2022). Potentilla anserine L. polysaccharide protects against cadmium-induced neurotoxicity. Environ. Toxicol. Pharmacol. 90, 103816. doi:10.1016/j.etap.2022.103816

Dong, Y., Wang, F., and Jin, M. (2025). Morinda officinalis polysaccharide exerts anti-Parkinson's disease effect via inhibiting NLRP3 inflammasome and improving pyroptosis of dopaminergic neurons. Mol. Cell. Toxicol.. Advance online publication. doi:10.1007/s13273-025-00541-2

Du, J., Chen, Z., Chen, X., Zhang, J., Wang, Y., Zhao, T., et al. (2024). Inhibition of Glycyrrhiza Polysaccharide on Human Cytochrome P450 46A1 in vitro and in vivo: Implications in Treating Neurological Diseases. Curr. Drug Metab. 25(3), 227-234. doi:10.2174/0113892002305873240520072802

Du, Q., Zhu, X., and Si, J. (2020). Angelica polysaccharide ameliorates memory impairment in Alzheimer's disease rat through activating BDNF/TrkB/CREB pathway. Exp. Biol. Med. 245(1), 1-10. doi:10.1177/1535370219894558

Fang, Y., Li, Y., Liao, X., Deng, J., Wang, Q., Liang, J.,et al. (2023). Corydalis yanhusuo Polysaccharides Ameliorate Chronic Stress-Induced Depression in Mice through Gut Microbiota-Derived Short-Chain Fatty Acid Activation of 5-Hydroxytryptamine Signaling. J. Med. Food 26(12), 890-901. doi:10.1089/jmf.2023.K.0050

Fu, J., Liang, Z., Chen, Z., Chen, W., Zhou, Y., Xiong, F., et al. (2025). Mechanism of Dendrobium officinale polysaccharide in alleviating Alzheimer's disease: Insights from metabolomics, lipidomics, and proteomics analysis. Int. J. Biol. Macromol. 319(Pt 2), 145531. doi:10.1016/j.ijbiomac.2025.145531

Gan, Q. X., Peng, M. Y., Wei, H. B., Chen, L. L., Chen, X. Y., Li, Z. H., et al. (2024). Gastrodia elata polysaccharide alleviates Parkinson's disease via inhibiting apoptotic and inflammatory signaling pathways and modulating the gut microbiota. Food Funct. 15(6), 2920-2938. doi:10.1039/d3fo05169b

He, Y., Xu, W., and Qin, Y. (2020). Structural characterization and neuroprotective effect of a polysaccharide from Corydalis yanhusuo. Int. J. Biol. Macromol. 157, 759-768. doi:10.1016/j.ijbiomac.2020.01.180

Hou, B. Y., Wu, M. H., Hsu, H. Y., Lin, Y. C., and Yang, D. I. (2024). Polysaccharides from Basella alba Protect Post-Mitotic Neurons against Cell Cycle Re-Entry and Apoptosis Induced by the Amyloid-Beta Peptide by Blocking Sonic Hedgehog Expression. Int. J. Mol. Sci. 25(13), 7316. doi:10.3390/ijms25137316

Hu, J. J., Liu, X., Xia, S., Zhang, Z., Zhang, Y., Zhao, J., et al. (2020). FDA-approved disulfiram inhibits pyroptosis by blocking gasdermin D pore formation. Nat. Immunol. 21(7), 736-745. doi:10.1038/s41590-020-0669-6

Hu, Y. R., Xing, S. L., Chen, C., Shen, D. Z., and Chen, J. L. (2021). Codonopsis pilosula Polysaccharides Alleviate Aβ 1-40-Induced PC12 Cells Energy Dysmetabolism via CD38/NAD+ Signaling Pathway. Curr. Alzheimer Res. 18(3), 208-221. doi:10.2174/1567205018666210608103831

Hu, Z., Li, F., Zhou, X., Zhang, F., Huang, L., Gu, B., et al. (2020). Momordica charantia polysaccharides modulate the differentiation of neural stem cells via SIRT1/B-catenin axis in cerebral ischemia/reperfusion. Stem Cell Res. Ther. 11(1), 485. doi:10.1186/s13287-020-02000-2

Jia, X., Xie, L., Liu, Y., Liu, T., Yang, P., Hu, J., et al. (2022). Astragalus polysaccharide (APS) exerts protective effect against acute ischemic stroke (AIS) through enhancing M2 micoglia polarization by regulating adenosine triphosphate (ATP)/ purinergic receptor (P2X7R) axis. Bioengineered 13(2), 4468-4480. doi:10.1080/21655979.2021.1980176

Jin, C., Du, Z., Lin, L., Zhou, L., Li, S., Liu, Q., et al. (2017). Structural characterization of mannoglucan from Dendrobium nobile Lindl and the neuritogenesis-induced effect of its acetylated derivative on PC-12 cells. Polymers 9(9), 399. doi:10.3390/polym9090399

Lan, Y., Ma, Z., Chang, L., Peng, J., Zhang, M., Sun, Q., et al. (2023). Sea buckthorn polysaccharide ameliorates high-fat diet induced mice neuroinflammation and synaptic dysfunction via regulating gut dysbiosis. Int. J. Biol. Macromol. 236, 123797. doi:10.1016/j.ijbiomac.2023.123797

Li, Q. M., Xu, H., Zha, X. Q., Zhang, F. Y., and Luo, J. P. (2025). Polygonatum cyrtonema polysaccharide alleviates dopaminergic neuron apoptosis in Parkinson's disease mouse model via inhibiting oxidative stress and endoplasmic reticulum stress. Int. J. Biol. Macromol. 311(Pt 3), 143986. doi:10.1016/j.ijbiomac.2025.143986

Li, R., Duan, W., Feng, T., Gu, C., Zhang, Q., Long, J., et al. (2023). Lycium barbarum polysaccharide inhibits ischemia-induced autophagy by promoting the biogenesis of neural stem cells-derived extracellular vesicles to enhance the delivery of miR-133a-3p. Chin. Med. 18(1), 117. doi:10.1186/s13020-023-00831-8

Li, Y., Liu, J., Pei, D., and Di, D. (2025). Structural Characterization of, and Protective Effects Against, CoCl2-Induced Hypoxia Injury to a Novel Neutral Polysaccharide from Lycium barbarum L. Foods 14(3), 339. doi:10.3390/foods14030339

Li, Y., Wang, B., Liu, C., Zhu, X., Zhang, P., Yu, H., et al. (2019). Inhibiting c-Jun N-terminal kinase (JNK)-mediated apoptotic signaling pathway in PC12 cells by a polysaccharide (CCP) from Coptis chinensis against Amyloid-β (Aβ)-induced neurotoxicity. Int. J. Biol. Macromol. 134, 565-574. doi:10.1016/j.ijbiomac.2019.05.041

Lin, C., Su, Z., Luo, J., Jiang, L., Shen, S., Zheng, W., et al. (2020). Polysaccharide extracted from the leaves of Cyclocarya paliurus (Batal.) Iljinskaja enhanced stress resistance in Caenorhabditis elegans via skn-1 and hsf-1. Int. J. Biol. Macromol. 143, 243-254. doi:10.1016/j.ijbiomac.2019.12.023

Liu, D., Zhu, Y., Hou, Z., Wang, H., and Li, Q. (2025). Polysaccharides from Astragalus membranaceus Bunge alleviate LPS-induced neuroinflammation in mice by modulating microbe-metabolite-brain axis and MAPK/NF-κB signaling pathway. International Int. J. Biol. Macromol. 304(Pt 1), 140885. doi:10.1016/j.ijbiomac.2025.140885

Liu, W., Yang, M., Wang, N., Liu, X., Wang, C., Shi, K., et al. (2025). Intracalvariosseous injection: an approach for central nervous system drug delivery through skull bone marrow with a preclinical research in stroke. EBioMedicine 112, 105568. doi:10.1016/j.ebiom.2025.105568

Liu, Y. C., Chen, S. Y., Chen, Y. Y., Chang, H. Y., Chiang, I. C., and Yen, G. C. (2024). Polysaccharides extracted from common buckwheat (Fagopyrum esculentum) attenuate cognitive impairment via suppressing RAGE/p38/NF-κB signaling and dysbiosis in AlCl3-treated rats. Int. J. Biol. Macromol. 276(Pt 2), 133898. doi:10.1016/j.ijbiomac.2024.133898

Liu, Y., , Liu, Z., , Wei, M., , Hu, M., , Yue, K., , Bi, R., , et al. (2019). Pharmacodynamic and urinary metabolomics studies on the mechanism of Schisandra polysaccharide in the treatment of Alzheimer's disease. Food Funct. 10(1), 432-447. doi:10.1039/c8fo02067a

Liu, Y., Wang, H., Yang, M., Liu, N., Zhao, Y., Qi, X., et al. (2018). Cistanche deserticola polysaccharides protects PC12 cells against OGD/RP-induced injury. Biomed. Pharmacother. 99, 671-680. doi:10.1016/j.biopha.2018.01.114

Luo, S., Zhang, X., Huang, S., Feng, X., Zhang, X., and Xiang, D. (2022). A monomeric polysaccharide from Polygonatum sibiricum improves cognitive functions in a model of Alzheimer's disease by reshaping the gut microbiota. Int. J. Biol. Macromol. 213, 404-415. doi:10.1016/j.ijbiomac.2022.05.185

Ma, S., Liu, X., Cheng, B., Jia, Z., Hua, H., and Xin, Y. (2019). Chemical characterization of polysaccharides isolated from scrophularia ningpoensis and its protective effect on the cerebral ischemia/reperfusin injury in rat model. Int. J. Biol. Macromol. 139, 955-966. doi:10.1016/j.ijbiomac.2019.08.040

Meng, H., Wu, J., Shen, L., Chen, G., Jin, L., Yan, M., et al. (2022). Microwave assisted extraction, characterization of a polysaccharide from Salvia miltiorrhiza Bunge and its antioxidant effects via ferroptosis-mediated activation of the Nrf2/HO-1 pathway. Int. J. Biol. Macromol. 215, 398-412. doi:10.1016/j.ijbiomac.2022.06.064

Sheng, Y., Liu, G., Wang, M., Lv, Z., and Du, P. (2017). A selenium polysaccharide from Platycodon grandiflorum rescues PC12 cell death caused by H2O2 via inhibiting oxidative stress. Int. J. Biol. Macromol. 104(Pt A), 393-399. doi:10.1016/j.ijbiomac.2017.06.052

Shi, J., Wang, J., Zhang, J., Li, X., Tian, X., Wang, W., et al. (2020). Polysaccharide extracted from Potentilla anserina L ameliorate acute hypobaric hypoxia-induced brain impairment in rats. Phytother. Res. 34(9), 2397-2407. doi:10.1002/ptr.6691

Shi, Z., Zhu, L., Li, T., Tang, X., Xiang, Y., Han, X., et al. (2017). Neuroprotective Mechanisms of Lycium barbarum Polysaccharides Against Ischemic Insults by Regulating NR2B and NR2A Containing NMDA Receptor Signaling Pathways. Front. Cell. Neurosci. 11, 288. doi:10.3389/fncel.2017.00288

Song, J., Liu, L., Li, Z., Mao, T., Zhang, J., Zhou, L., et al. (2022). Lycium barbarum polysaccharide improves dopamine metabolism and symptoms in an MPTP-induced model of Parkinson's disease. BMC Med. 20(1), 412. doi:10.1186/s12916-022-02621-9

Sun, Q., Shi, P., Lin, C., and Ma, J. (2020). Effects of Astragalus Polysaccharides Nanoparticles on Cerebral Thrombosis in SD Rats. Front. Bioeng. Biotechnol. 8, 616759. doi:10.3389/fbioe.2020.616759

Sun, Y., Zeng, X., Liu, Y., Zhan, S., Wu, Z., Zheng, X., et al. (2022). Dendrobium officinale polysaccharide attenuates cognitive impairment in circadian rhythm disruption mice model by modulating gut microbiota. Int. J. Biol. Macromol. 217, 677-688. doi:10.1016/j.ijbiomac.2022.07.090

Tan, Y., Yin, L., Sun, Z., Shao, S., Chen, W., Man, X., et al. (2020). Astragalus polysaccharide exerts anti-Parkinson via activating the PI3K/AKT/mTOR pathway to increase cellular autophagy level in vitro. Int. J. Biol. Macromol. 153, 349-356. doi:10.1016/j.ijbiomac.2020.02.282

Wang, J., Liu, P., Huang, X., and Wu, X. (2021). Validation of the protective effects of Lonicera japonica polysaccharide on lipopolysaccharide-induced learning and memory impairments via regulation of autophagy based on network pharmacology. Ann. Palliat. Med. 10(2), 1089-1100. doi:10.21037/apm-20-357

Wang, X., Pang, L., Zhang, Y., Xu, J., Ding, D., Yang, T.,et al. (2018). Lycium barbarum Polysaccharide Promotes Nigrostriatal Dopamine Function by Modulating PTEN/AKT/mTOR Pathway in a Methyl-4-phenyl-1,2,3,6-tetrahydropyridine (MPTP) Murine Model of Parkinson's Disease. Neurochem. Res. 43(4), 938-947. doi:10.1007/s11064-018-2499-6

Xiang, Y., Zhang, J., Li, H., Wang, Q., Xiao, L., Weng, H., et al. (2017). Epimedium Polysaccharide Alleviates Polyglutamine-Induced Neurotoxicity in Caenorhabditis elegans by Reducing Oxidative Stress. Rejuvenation Res. 20(1), 32-41. doi:10.1089/rej.2016.1830

Xie, P., Chen, L., Wang, J., Wang, X., Yang, S., and Zhu, G. (2024). Polysaccharides from Polygonatum cyrtonema Hua prevent post-traumatic stress disorder behaviors in mice: Mechanisms from the perspective of synaptic injury, oxidative stress, and neuroinflammation. J. Ethnopharmacol. 319(Pt 1), 117165. doi:10.1016/j.jep.2023.117165

Xie, Y., Zhang, H., Zhang, Y., Wang, C., Duan, D., and Wang, Z. (2018). Chinese Angelica Polysaccharide (CAP) Alleviates LPS-Induced Inflammation and Apoptosis by Down-Regulating COX-1 in PC12 Cells. Cell. Physiol. Biochem. 49(4), 1380-1388. doi:10.1159/000493415

Xu, H., Chen, J., Liu, W., Li, H., Yu, Z., and Zeng, C. (2021). The effect of Angelica sinensis polysaccharide on neuronal apoptosis in cerebral ischemia-reperfusion injury via PI3K/AKT pathway. Int. J. Polym. Sci. 2021(1), 7829341. doi:10.1155/2021/7829341

Xu, L., Liu, H., Rang, Y., Zhou, L., Wang, X., Li, Y., et al. (2024). Lycium barbarum polysaccharides attenuate nonylphenol and octylphenol-induced oxidative stress and neurotransmitter disorders in PC-12 cells. Toxicology 505, 153808. doi:10.1016/j.tox.2024.153808

Xu, M., Wang, J., Zhang, X., Yan, T., Wu, B., Bi, K., et al. (2020). Polysaccharide from Schisandra chinensis acts via LRP-1 to reverse microglia activation through suppression of the NF-κB and MAPK signaling. J. Ethnopharmacol. 256, 112798. doi:10.1016/j.jep.2020.112798

Xu, M., Yan, T., Fan, K., Wang, M., Qi, Y., Xiao, F., et al. (2019). Polysaccharide of Schisandra Chinensis Fructus ameliorates cognitive decline in a mouse model of Alzheimer's disease. J. Ethnopharmacol. 237, 354-365. doi:10.1016/j.jep.2019.02.046

Xue, Y., Dongmei Li, Yige Zhang, Hang Gao, and Li, H. (2019). Angelica polysaccharide moderates hypoxia-evoked apoptosis and autophagy in rat neural stem cells by downregulation of BNIP3. Artif. Cells Nanomed. Biotechnol. 47(1), 2492-2499. doi:10.1080/21691401.2019.1623228

Yang, S., Wang, L., Xie, Z., Zeng, Y., Xiong, Q., Pei, T., et al. (2022). The Combination of Salidroside and Hedysari Radix Polysaccharide Inhibits Mitochondrial Damage and Apoptosis via the PKC/ERK Pathway. Evid. Based Complement. Alternat. Med. 2022, 9475703. doi:10.1155/2022/9475703

Yang, Y., Yu, L., Zhu, T., Xu, S., He, J., Mao, N., et al. (2023). Neuroprotective effects of Lycium barbarum polysaccharide on light-induced oxidative stress and mitochondrial damage via the Nrf2/HO-1 pathway in mouse hippocampal neurons. Int. J. Biol. Macromol. 251, 126315. doi:10.1016/j.ijbiomac.2023.126315

Yang, Y., Yu, L., Zhu, T., Xu, S., He, J., Mao, N., et al. (2024). Neuroprotective effects of Rehmannia glutinosa polysaccharide on chronic constant light (CCL)-induced oxidative stress and autophagic cell death via the AKT/mTOR pathway in mouse hippocampus and HT-22 cells. Int. J. Biol. Macromol. 261(Pt 2), 129813. doi:10.1016/j.ijbiomac.2024.129813

Ye, N., Cruz, J., Peng, X., Ma, J., Zhang, A., and Cheng, X. (2021). Remyelination is enhanced by Astragalus polysaccharides through inducing the differentiation of oligodendrocytes from neural stem cells in cuprizone model of demyelination. Brain Res. 1763, 147459. doi:10.1016/j.brainres.2021.147459

Yu, Y., Wu, X., Pu, J., Luo, P., Ma, W., Wang, J., et al. (2018). Lycium barbarum polysaccharide protects against oxygen glucose deprivation/reoxygenation-induced apoptosis and autophagic cell death via the PI3K/Akt/mTOR signaling pathway in primary cultured hippocampal neurons. Biochem. Biophys. Res. Commun. 495(1), 1187-1194. doi:10.1016/j.bbrc.2017.11.165

Yue, J., Guo, P., Jin, Y., Li, M., Hu, X., Wang, W., et al. (2023). Momordica charantia polysaccharide ameliorates D-galactose-induced aging through the Nrf2/β-Catenin signaling pathway. Metab. Brain. Dis. 38(3), 1067-1077. doi:10.1007/s11011-022-01103-4

Zeng, H., Huang, L., Tao, H., Zhang, Y., and Ding, K. (2020). Structural elucidation of a pectin from roots of Polygala tenuifolia and its neuritogenesis inducing activity in PC12 cells. Carbohydr. Polym. 236, 116048. doi:10.1016/j.carbpol.2020.116048

Zhang, G., Huang, J., Hao, S., Zhang, J., and Zhou, N. (2022). Radix Astragalus Polysaccharide Accelerates Angiogenesis by Activating AKT/eNOS to Promote Nerve Regeneration and Functional Recovery. Front Pharmacol 13, 838647. doi:10.3389/fphar.2022.838647

Zhang, X., Ye, Y., Sun, J., Xu, Y., Huang, Y., Wang, J.S., et al. (2022) Polygonatum sibiricum polysaccharide extract relieves FB1-induced neurotoxicity by reducing oxidative stress and mitochondrial damage in Caenorhabditis elegans, Food Biosci. 49, 101939. doi:10.1016/j.fbio.2022.101939

Zhang, Y., Ye, P., Zhu, H., Gu, L., Li, Y., Feng, S., et al. (2024). Neutral polysaccharide from Gastrodia elata alleviates cerebral ischemia-reperfusion injury by inhibiting ferroptosis-mediated neuroinflammation via the NRF2/HO-1 signaling pathway. CNS Neurosci. Ther. 30(3), e14456. doi:10.1111/cns.14456

Zhao, H., Liu, J., Wang, Y., Shao, M., Wang, L., Tang, W., et al. (2023). Polysaccharides from sea buckthorn (Hippophae rhamnoides L.) berries ameliorate cognitive dysfunction in AD mice induced by a combination of d-gal and AlCl3 by suppressing oxidative stress and inflammation reaction. J. Sci. Food Agric. 103(12), 6005-6016. doi:10.1002/jsfa.12673

Zhao, P., Zhou, R., Zhu, X. Y., Liu, G., Zhao, Y. P., Ma, P. S., et al. (2017). Neuroprotective Effects of Lycium barbarum Polysaccharide on Focal Cerebral Ischemic Injury in Mice. Neurochem. Res. 42(10), 2798-2813. doi:10.1007/s11064-017-2293-x

Zhao, Y., Liu, X., Zheng, Y., Liu, W., and Ding, C. (2021). Aronia melanocarpa polysaccharide ameliorates inflammation and aging in mice by modulating the AMPK/SIRT1/NF-κB signaling pathway and gut microbiota. Sci. Rep. 11(1), 20558. doi:10.1038/s41598-021-00071-6

Zhao, Y., Ma, J., Ding, G., Wang, Y., Yu, H., and Cheng, X. (2024). Astragalus polysaccharides promote neural stem cells-derived oligodendrogenesis through attenuating CD8+T cell infiltration in experimental autoimmune encephalomyelitis. Int. Immunopharmacol. 126, 111303. doi:10.1016/j.intimp.2023.111303

Zhong, J., Qiu, X., Yu, Q., Chen, H., and Yan, C. (2020). A novel polysaccharide from Acorus tatarinowii protects against LPS-induced neuroinflammation and neurotoxicity by inhibiting TLR4-mediated MyD88/NF-κB and PI3K/Akt signaling pathways. Int. J. Biol. Macromol. 163, 464-475. doi:10.1016/j.ijbiomac.2020.06.266

Zhou, Y., Zhu, L., Li, H., Xie, W., Liu, J., Zhang, Y., et al. (2022). In vivo and in vitro neuroprotective effects of maca polysaccharide. Front. Biosci. 27(1), 8. doi:10.31083/j.fbl2701008

Zhu, Z., Song, X., Jiang, Y., Yao, J., Jiang, Y., Li, Z., et al. (2022). Chemical structure and antioxidant activity of a neutral polysaccharide from Asteris Radix et Rhizoma. Carbohydr. Polym. 286, 119309. doi:10.1016/j.carbpol.2022.119309

Zou, X., Yang, H., Li, Q., Li, N., Hou, Y., Wang, X., et al. (2022). Protective Effect of Brassica rapa Polysaccharide against Acute High-Altitude Hypoxia-Induced Brain Injury and Its Metabolomics. Oxid. Med. Cell. Longev. 2022, 3063899. doi:10.1155/2022/3063899
